# Some thio­ether-ketones and their related derivatives

**DOI:** 10.1107/S2056989025004037

**Published:** 2025-05-13

**Authors:** Molly A. O’Connor, Anna V. Pavlishchuk, Raymond J. Butcher, Vitaly V. Pavlishchuk, Anthony W. Addison

**Affiliations:** a Department of Chemistry Drexel University, 3141 Chestnut St Philadelphia PA 19104-2816 USA; b L. V. Pisarzhevskii Institute of Physical Chemistry of the National Academy of Sciences of Ukraine Prospect Nauki 31 Kyiv 03028 Ukraine; c Department of Chemistry, Purdue University, 560 Oval Drive, West Lafayette, 47907-2084, IN, USA; d Chemistry Department Howard University, 525 College St NW Washington DC 20059 USA; Texas A & M University, USA

**Keywords:** crystal structure, thio­ether ketones derivatives, intra- and inter­molecular hydrogen bonding

## Abstract

Structural characteristics are reported for two thio­ether–ketones, ***Dtdpe*** and ***Mtdp*** [2-({2-[(2-oxo-2-phenyl­eth­yl)sulfan­yl]eth­yl}sulfan­yl)-1-phenyl­ethan-1-one, C_18_H_18_O_2_S_2_, and 2-[(2-oxo-2-phenyl­eth­yl)sulfan­yl]-1-phenyl­ethan-1-one, C_16_H_14_O_2_S], and for related derivatives, the bis­(pyridyl­hydrazones) ***Dhpk*** and ***Prpsb*** [2-((2*E*)-2-{(2*Z*)-2-phenyl-2-[2-(pyridin-2-yl)hydrazin-1-yl­idene]ethyl­idene}hydrazin-1-yl)pyridine, C_18_H_16_N_6_, and 2-[(2*Z*,12*Z*)-3,12-diphenyl-14-(pyridin-2-yl)-5,10-di­thia-1,2,13,14-tetra­aza­tetra­deca-2,12-dien-1-yl]pyridine, C_30_H_32_N_6_S_2_], as well as for the macrocyclic thio­carbohydrazide derivative ***Ctrsp***[(3*E*,8*Z*)-3,9-dimethyl-1,11-di­thia-4,5,7,8-tetra­aza­cyclo­tetra­deca-3,8-diene-6-thione, C_10_H_18_N_4_S_3_]. Three of the five compounds exhibit conformational enanti­omerism in the solid state. The occurrence of intra- and inter­molecular hydrogen bonding is commented upon through quantum mechanical (DFT) calculations. Weak C—H⋯S inter­actions are noted, while stronger N—H⋯N and N—H⋯S hydrogen bridges are delineated.

## Abstract 2

Structures are reported for two thio­ether-ketones, and for some derived hydrazones, and instances of conformational enanti­omerism are delineated. Various types of hydrogen bonds, such as weak C—H⋯S and stronger N—H⋯N and N—H⋯S hydrogen bridges are noted, and inter­molecular cases examined *via* DFT calculations.

## 1. Chemical context

The rational structural design of coordination compounds is crucial for the accomplishment of the desired properties of such complexes and their further functionality. The main focus in the genesis of targeted coordination compounds is usually concentrated on the creation of a proper donor-atom environment around the central metal atom. Despite the fact that the donor set composition is in most cases determinative for the generation of one or another feature, it has been shown that peculiarities of the spatial organization of coordination units in the solid state may significantly influence such properties (Steed *et al.*, 2007 ▸; Mikhalyova *et al.*, 2015 ▸). Weak inter­actions such as different *intra*- and *inter­molecular* π–π inter­actions and hydrogen bonds between some fragments of ligands are important for the crystal packing of coordination complex mol­ecules. While the occurrence of such inter­actions is commonly reported for complexes, such data for non-coordinated ligands are rare. However, the elucidation of details of the mol­ecular and crystal structures of ligands may contribute a significant insight for the understanding of the structural features of the complexes and give important information for the crystal engineering of metal complexes. For instance, the hydrazone N—H entities act as hydrogen-bond donors to perchlorate and triflate anions in the structures of nickel(II) complexes of directly related pyridyl-hydrazone ligands (Pavlishchuk *et al.*, 2024 ▸).
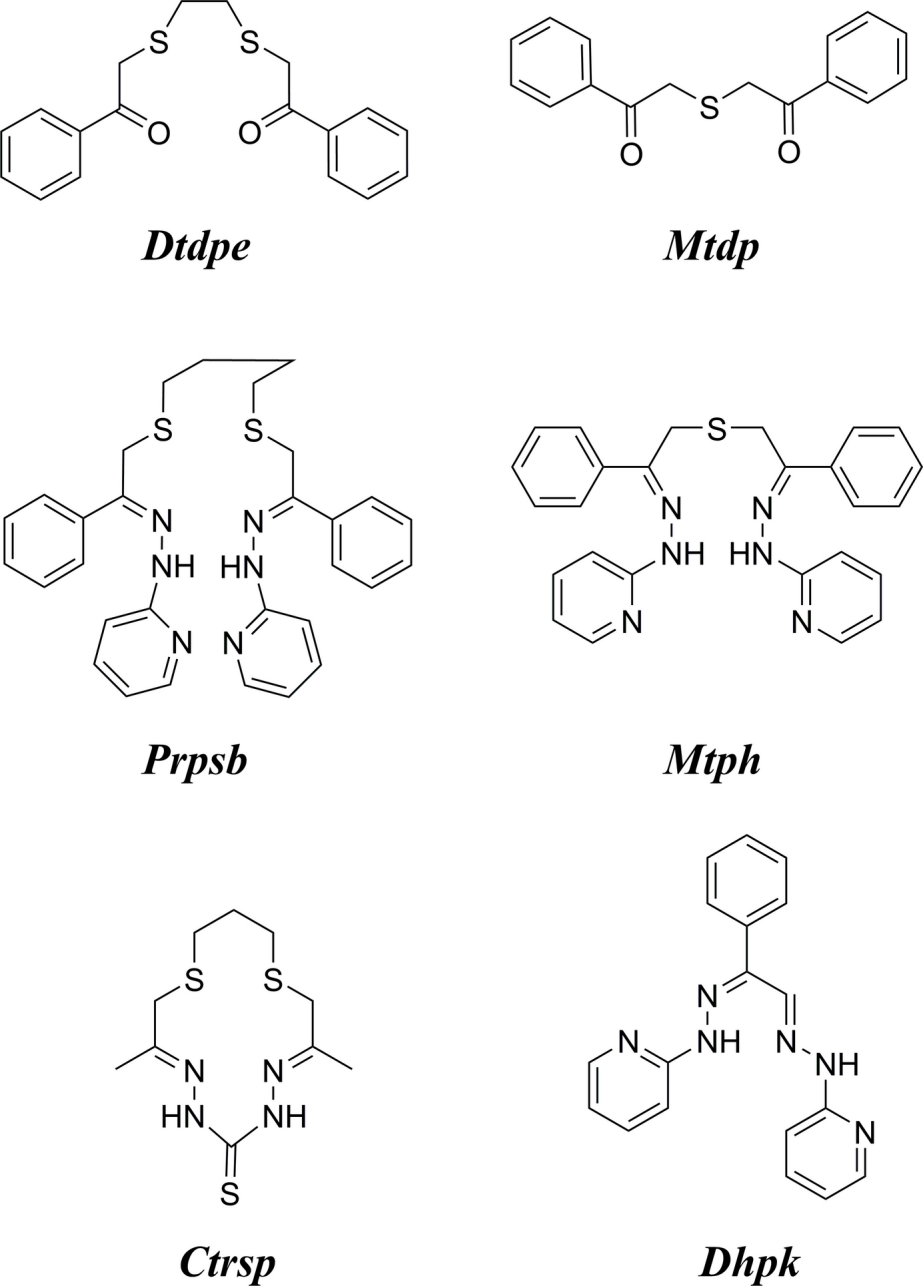


**Figure 1:**
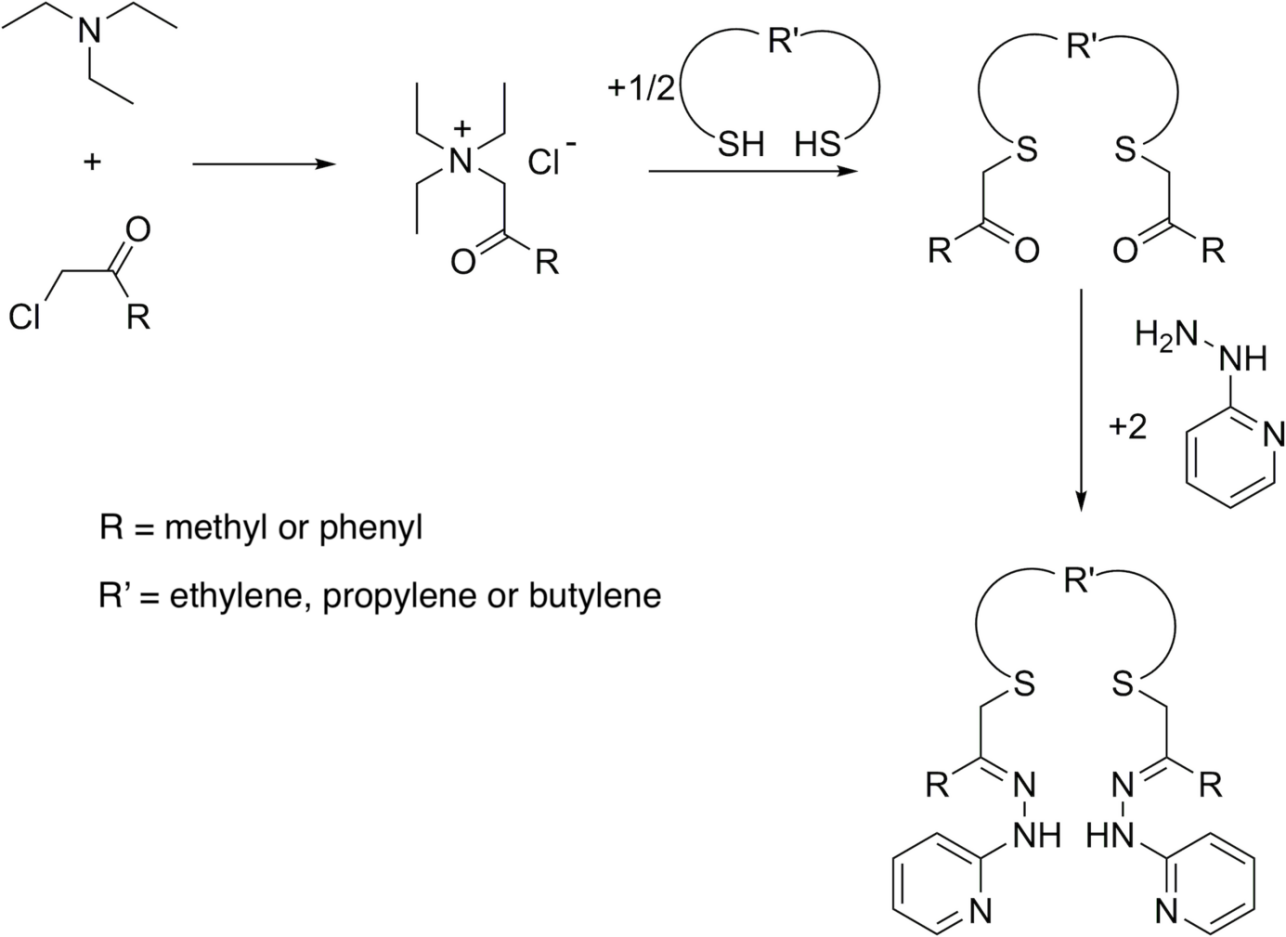
General synthesis procedure for the thio­ether ketones and for formation of a pyridyl­hydrazone derivative.

Here we report the synthesis and crystal structures of a new set of some thio­ether-ketones and their derivatives – promising ‘soft’ ligands for the stabilization of reduced oxidation states of metal ions. The general synthesis procedure for thio­ether ketones and for the formation of a pyridyl­hydrazone derivative is shown in Fig. 1 ▸.

## 2. Structural commentary

**Figure 2:**
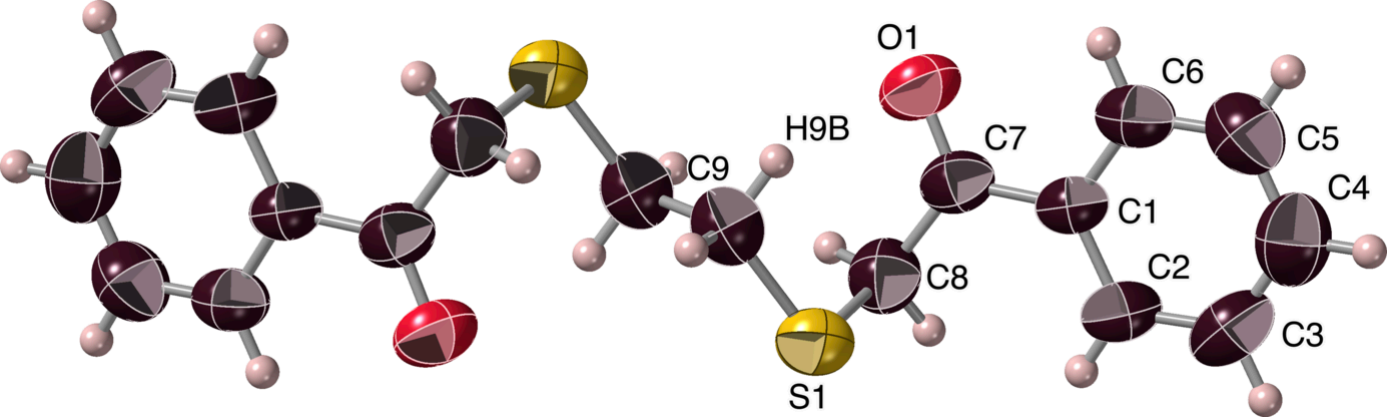
The ***Dtdpe*** mol­ecule, with its atom-numbering scheme. Hydrogen atoms are represented as simple spheres, with only those that are specifically discussed being labelled, and displacement ellipsoids are displayed at the 50% probability level.

***Dtdpe***, 1,8-diphenyl-3,6-di­thia­octane-1,8-dione or 2-({2-[(2-oxo-2-phenyl­eth­yl)sulfan­yl]eth­yl}sulfan­yl)-1-phenyl­ethan-1-one, the diketone precursor of the dihydrazone ***Prpse*** (Pavlishchuk *et al.*, 2024 ▸), crystallized from MeOH as thin, transparent, pale-yellow prisms in the monoclinic space group *P2_1_/c*. The ***Dtdpe*** mol­ecule with the atom numbering is shown in Fig. 2 ▸. Potential intra­molecular hydrogen-bond metrics are given in Table 1 ▸. Each mol­ecule is conformed so as to possess two ‘steps’ between the ends with the phenyl groups parallel to each other. The alkyl carbon atom C9 is 3.229 (3) Å from O1, which meets Steiner & Desiraju’s O⋯C distance criterion (3–4 Å (Steiner, 1994 ▸, 1996 ▸; Desiraju, 1991 ▸, 1996 ▸, 2002 ▸) for a weak intra­molecular inter­action. However the angular characteristics (Table 1 ▸, type #1) of the C9—H9*B*⋯O1 fragment are at best only marginally suitable (Desiraju, 1996 ▸, 2011 ▸). A pervasive difficulty with such assignments rests with the arbitrary approximations made for H-atom positions *via*X-ray crystallography, (Desiraju 1991 ▸, 1995 ▸, 2005 ▸; Bulusu & Desiraju, 2020 ▸) as well as the energetics of competing structural/conformational factors, which detract from the clarity of gas-phase calculation results related to potential *inter­molecular* hydrogen bonds (*vide infra*).

**Figure 3:**
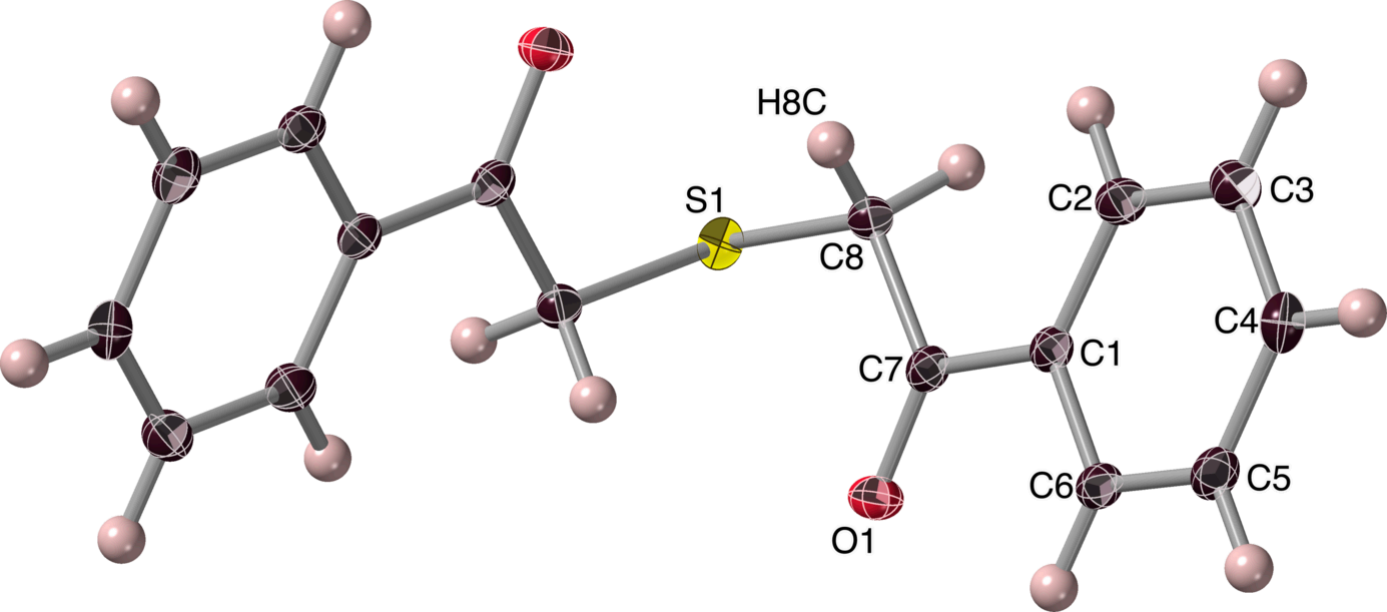
The ***Mtdp*** mol­ecule, with its atom-numbering scheme. Hydrogen atoms are represented as simple spheres, with only those that are specifically discussed being labelled, and displacement ellipsoids are displayed at the 50% probability level.

**Figure 4:**
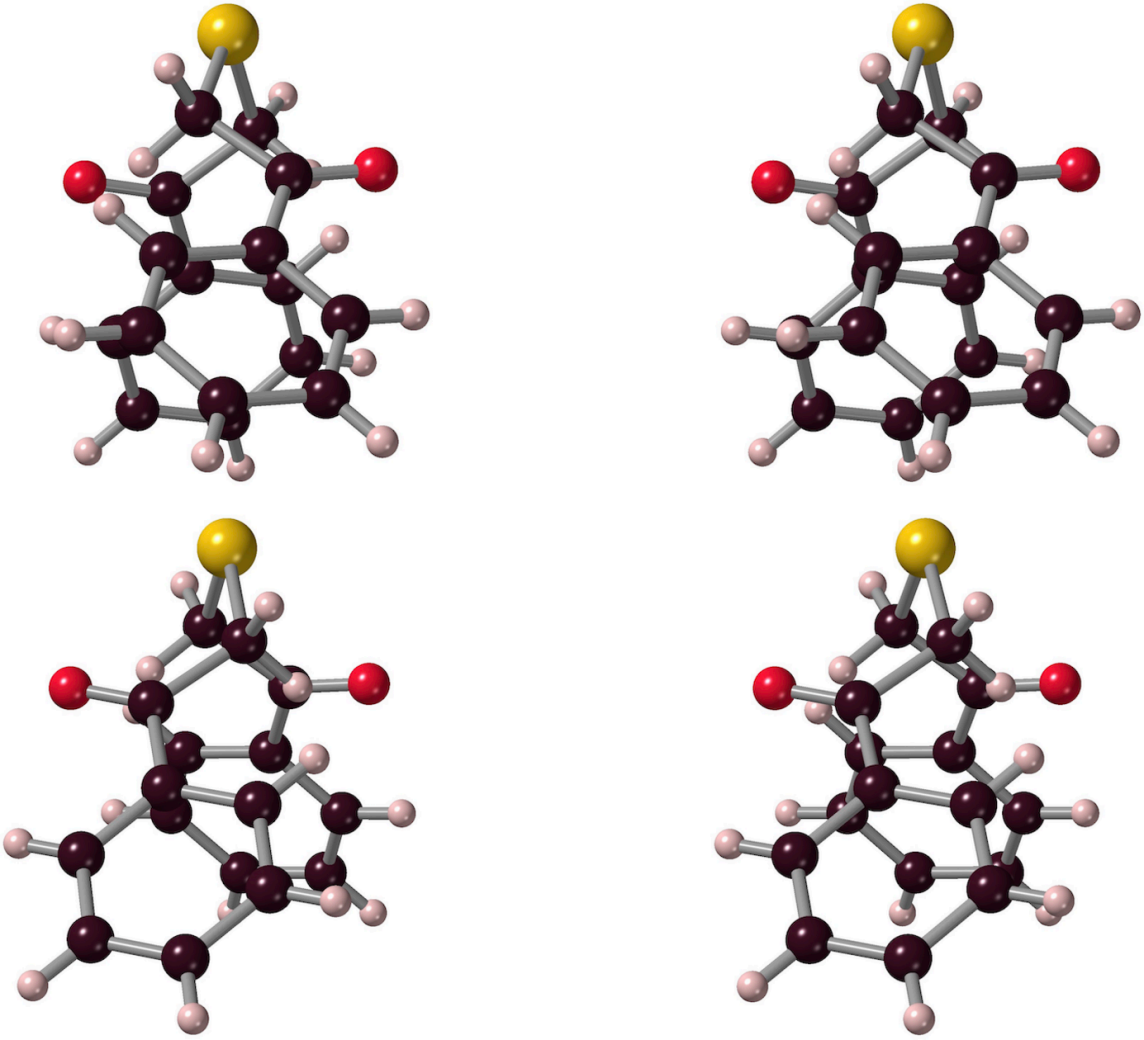
The conformational enanti­omers of ***Mtdp***: crossing the ‘bridge’ from front to rear places the front carbonyl to the right in one (upper) enanti­omer, and to the left in the other (lower). Ball-and-stick representations, inverse stereoviews.

The mono­thio-diketone ***Mtdp***, 1,5-diphenyl-3-thia­pentane-1,5-dione or 2-[(2-oxo-2-phenyl­eth­yl)sulfan­yl]-1-phenyl­ethan-1-one, crystallized from methanol as thin, slightly photosensitive colourless laths, belonging to the monoclinic space group *I*2/*a* (*C*2/*c*), with four asymmetric half-mol­ecule units in the unit cell. The structure, shown in Figs. 3 ▸, 4 ▸ and S1, suggests a bridge (‘*soribashi*’) shape for the dissymmetric mol­ecules, with the sulfur at bridge centre, so that crossing the bridge involves carbonyl oxygen atoms on the approach pointing to the left in one conformational enanti­omer, and to the right in the other (Fig. 4 ▸). Similarly to ***Dtdpe***, two equivalent potential weak intra­molecular hydrogen⋯O1 inter­actions exist, between O1 and the (more distant) H8*C* hydrogen, resulting in a six-membered cyclic arrangement O1–C7–C8–S1–C8^i^–H8*C*^i^ [symmetry code: (i) −*x* + 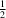, *y*, −*z* + 1). The metrics of this weak H8C⋯O1 inter­action (Table 1 ▸, #2) indicate that this inter­action is again quite marginal. Meanwhile, an intra­molecular inter­action between O1 and phenyl hydrogen H6*A* (Table 1 ▸, #3), produces a five-membered ring arrangement, O1–C7–C1–C6–H6*A* with O1 and C6 2.807 (2) Å apart, but with angular characteristics unsuitable for hydrogen bonding. For the ketonic C7—O1 bond, the length [1.215 (2) Å] is close to those observed in ***Dtdpe*** (*vide supra*), of a value typical for a Ph—C=O moiety (Tanimoto *et al.*, 1973 ▸; Seth *et al.*, 2011 ▸; Fleischer *et al.*, 1968 ▸) and not influenced by the neighbouring hydrogen inter­actions.

**Figure 5:**
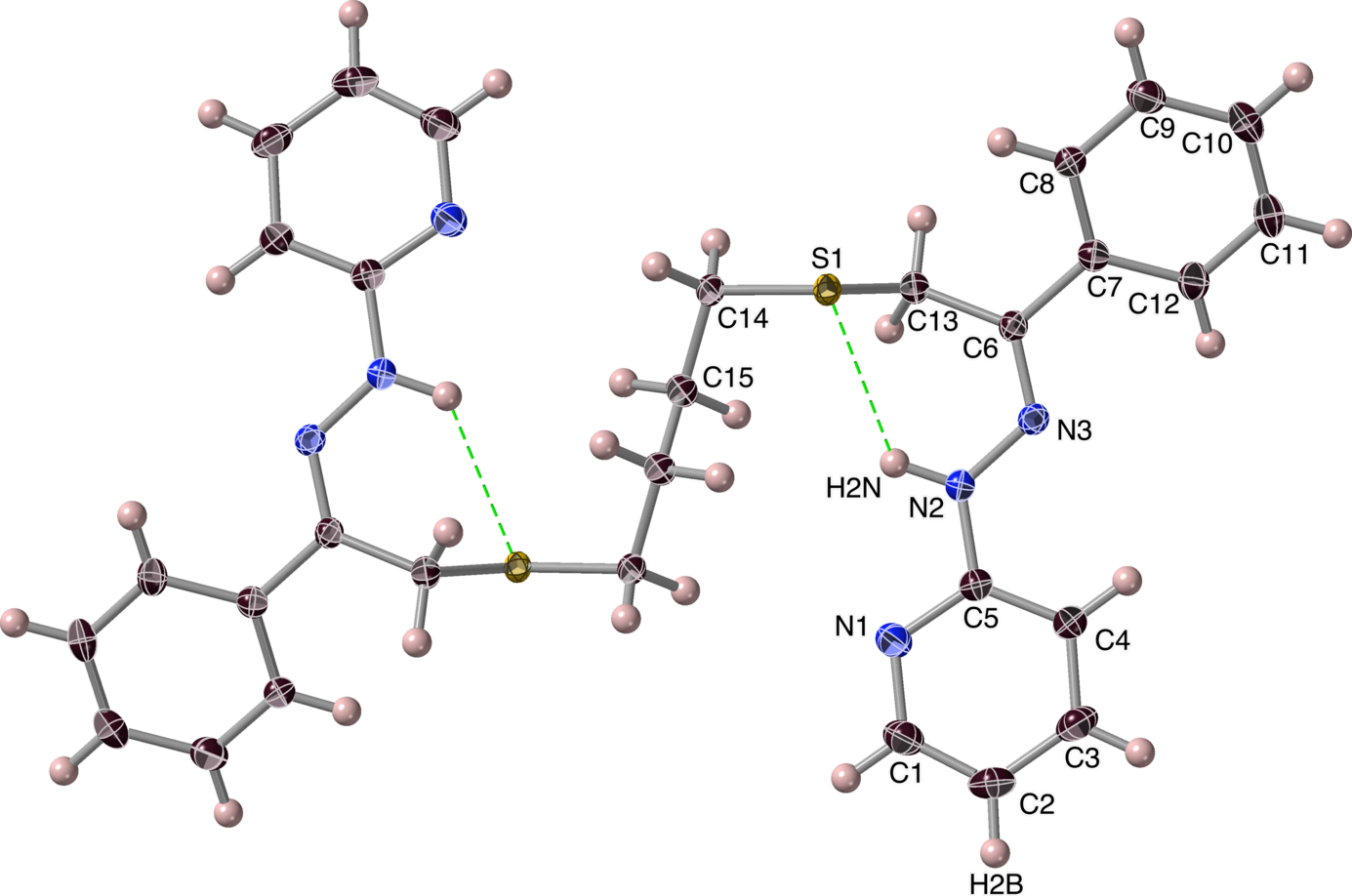
***Prpsb*** mol­ecule with the possible N—H⋯S hydrogen bonds shown. The pair of benzene rings are parallel, as are the pyridines. Hydrogen atoms are represented as simple spheres, with only those that are specifically discussed being labelled, and displacement ellipsoids are displayed at the 50% probability level.

**Figure 6:**
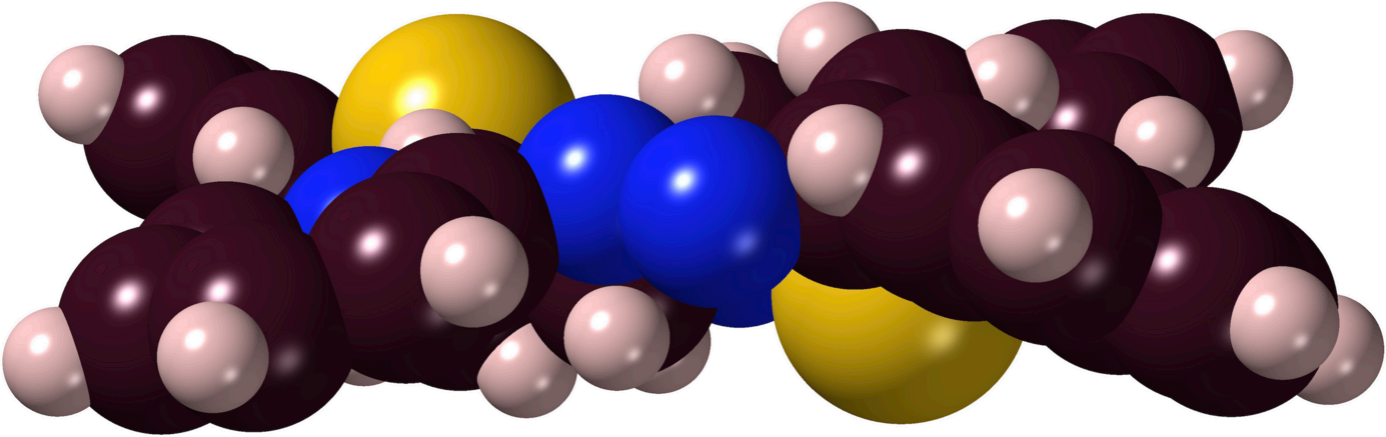
A space-filling lateral view of the ***Prpsb*** mol­ecule. The vertical ‘thickness’ is *ca*. 3.8 Å, compared with the ‘length’ of *ca*. 12.6 Å.

The di­thio­ether-dihydrazone ***Prpsb***, 2-[(2*Z*,12*Z*)-3,12-diphenyl-14-(pyridin-2-yl)-5,10-di­thia-1,2,13,14-tetra­aza­tetra­deca-2,12-dien-1-yl]pyridine, crystallized from ethanol as golden yellow crystals belonging to the centrosymmetric monoclinic space group *P*2_1_/*n* (*Z* = 2; Fig. 5 ▸) with the two half-mol­ecules being mirror images of one another (Fig. S2). The structural results evidence that the mol­ecule has *Z,Z*-stereochemistry about the C=N bonds, which may well be related to it not readily forming a nickel(II) complex (Pavlishchuk *et al.*, 2024 ▸), as those complexes have the ligands in the *E,E*-configuration. It has a perhaps surprisingly flat conformation, as suggested by the space-filling diagram (Fig. 6 ▸). The average non-H atom distance from the mean plane of the mol­ecule is 0.4 (0.2) Å.

Potentially significant hydrogen-bridging inter­actions occur intra­molecularly (Fig. 5 ▸, Table 1 ▸, #4). The N2⋯S1 distance is 3.3599 (15) Å [*cf*. covalent and van der Waals radii (Housecroft & Sharpe, 2012 ▸; Bird & Cheeseman, 1984 ▸) contact distance of 4.12 Å], while the N—H⋯S angle is 126°. These fit the distance criteria in accordance with spectroscopic and quantum mechanical calculation approaches (Biswal *et al.*, 2015 ▸), while the observed N2⋯S1 distance [3.3599 (15) Å] is close to those obtained for the formaldehyde-di­methyl­sulfide complex (3.200 Å) by FT microwave spectroscopy (Tatamitani *et al.*, 2015 ▸) and *ab initio* calculated for the indole-di­methyl­sulfide complex (3.327 Å) and for the 3-methyl­indole-di­methyl­sulfide complex (3.331Å) (Biswal & Wategaonkar, 2009 ▸). We are not aware of other crystallographic reports of such N—H⋯S hydrogen bonds.

**Figure 7:**
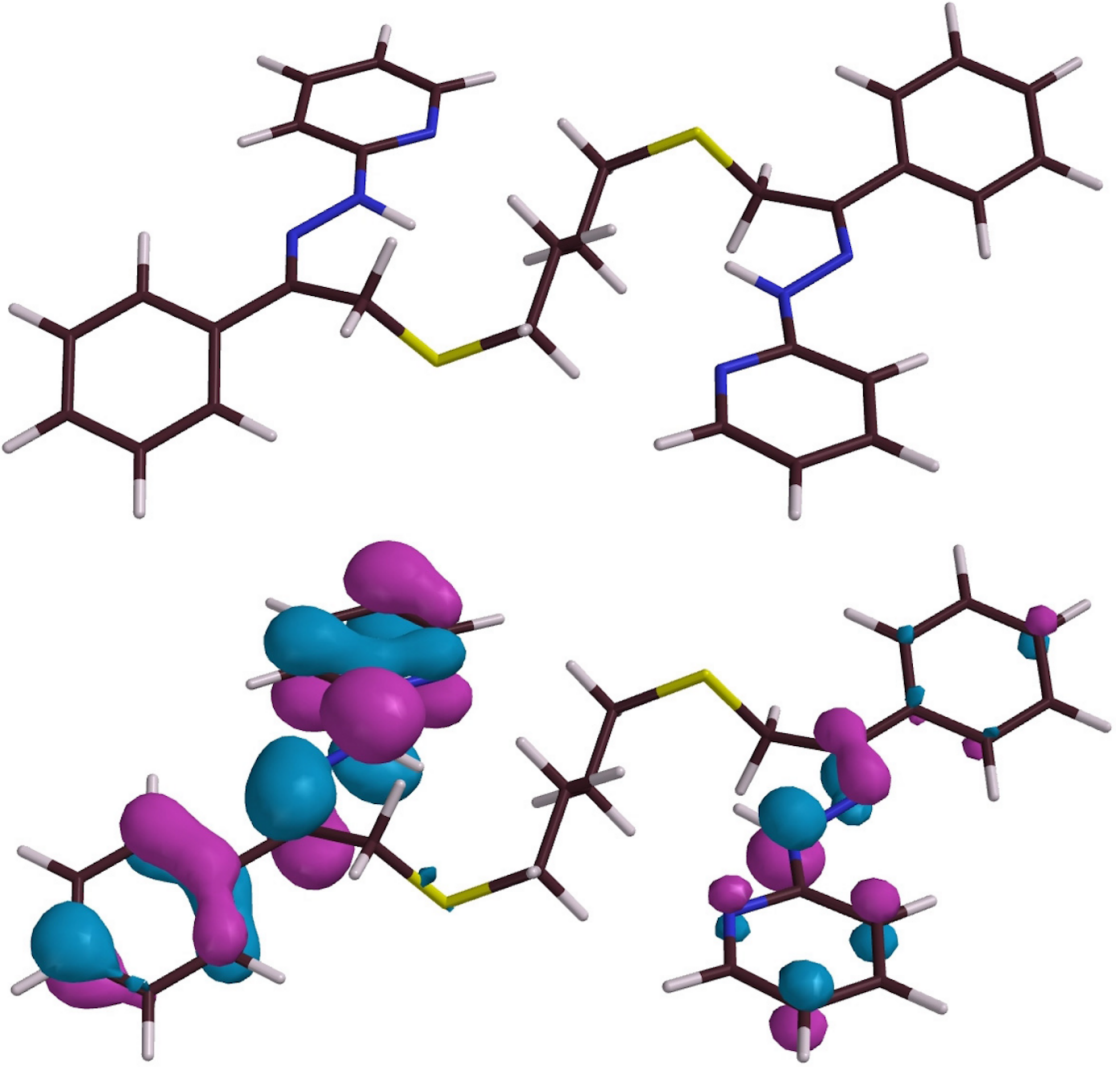
Upper, reference diagram of the ***Prpsb*** skeleton; lower, the ***Prpsb*** HOMO wavefunction (*Spartan-20/24*) surface in a structurally minimized mol­ecule.

The atom N2, although formally a hydrazine nitro­gen, is of a relatively planar geometry. The three angles with its surrounding atoms (H2*N*, N3, C5) sum to 355°, which is more like *sp^2^* hybridization (360°) than the angles in hydrazine itself (321°), while N2 is only 0.126 Å from the N3–H2*N*–N2–C5 mean plane (for which the sum of squares error, SSE, is only 0.022 Å^2^). This is slightly more than in a closely related complex [Ni(Prpse)]^2+^ (Pavlishchuk *et al.*, 2024 ▸) where further adjustment by metal coordination is likely. The broader atom set C6–N3–N2–H2*N*–C5–C4–N1 also exhibits a fair degree of planarity (SSE = 0.0348 Å^2^) and DFT calculations support the idea of conjugation extending from the pyridine ring through to the originally ketonic C6, with bonding π-character in both the hydrazone N2—N3 and pyridine α-C to N (C5—N2) bonds (Fig. 7 ▸). This planarity compares with a nonetheless incompletely continuous bonding π-system, as indicated by the HOMO’s (0, −1, −6, −7) (Fisg. 7 ▸, S3). A simple pyridyl­hydrazone (acetone pyridyl­hydrazone) model does show through-conjugation (Fig. S4) while by contrast, in some related Co^II^ complexes, *anti­bonding* π-MO’s appear to dominate this moiety (Pramanik *et al.*, 2014 ▸), so the electronic structure of such systems is very dependent upon the substituents and metal.

**Figure 8:**
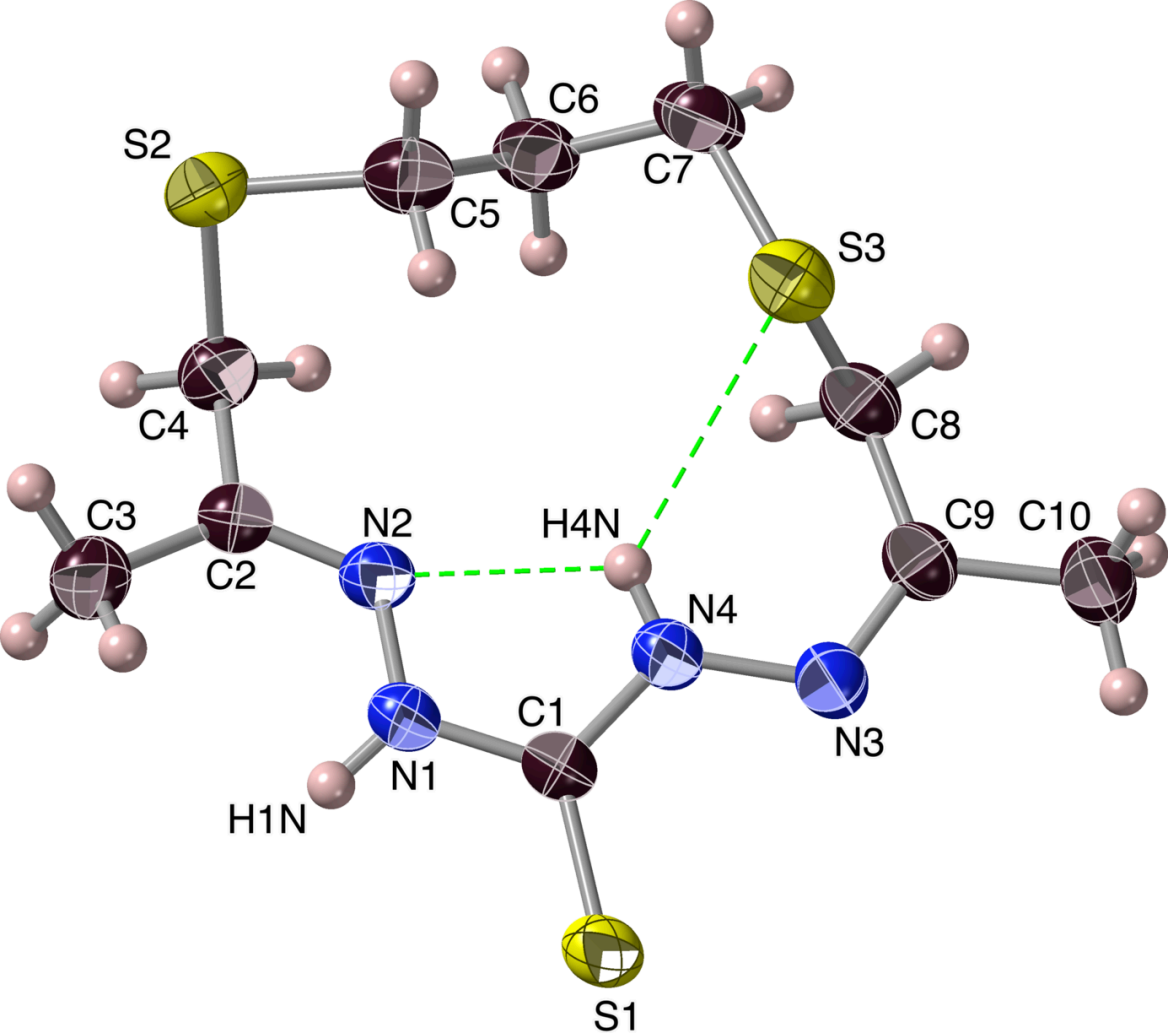
The macrocycle di­thio­ether-thio­hydrazone ***Ctrsp***, with its *crystallographic* numbering scheme. Hydrogen atoms are represented as simple spheres, with only those that are specifically discussed being labelled, and displacement ellipsoids are displayed at the 50% level. The organic nomenclature positions denoted as 1 and 11 are, respectively, S3 and S2. Potential N—H⋯N and N—H⋯S hydrogen bonds are shown in green.

The reaction between thio­carbohydrazide and ***Dtdkp*** (4,8-di­thia­undecane-2,10-dione; Pavlishchuk *et al.*, 2024 ▸) yielded a yellow microcrystalline product, for which the mass spectrum suggested the presence of two major components, at *m*/*z* = 397.1^+^ (for C_11_H_24_N_8_S_4_+H^+^) and 291.1^+^ (for C_10_H_18_N_4_S_3_+H^+^), corresponding to the values for the 1:2 and 1:1 hydrazones, respectively. Fractional recrystallization from 2-meth­oxy­ethanol gave rise to a low yield of pale-yellow 0.5-1 mm blocks, belonging to the space group *P*2_1_/*c* with four mol­ecules in the unit cell. The 1:1 product thus isolated by recrystallization proved to be the macrocyclic di­thio­ether-thio­hydrazone ***Ctrsp***, (3*E*,8*Z*)-3,9-dimethyl-1,11-di­thia-4,5,7,8-tetra­aza­cyclo­tetra­deca-3,8-diene-6-thione, for which its mol­ecular structure and atom-numbering scheme are shown in Fig. 8 ▸. The ***Ctrsp*** mol­ecule possesses *E*,*Z*-geometry about the hydrazone imines, with atoms N2 and N3 of each mol­ecule on opposite sides of the N1–C1–N4 unit, reducing the overall mol­ecular symmetry. Indeed, the individual mol­ecules are asymmetric because of conformational isomerism associated with relatively free rotations about the C—C and C—S bonds from C2 to C9 (Fig. S5). They hence relate to one another by inversion, resulting in two mol­ecules of each of the consequent enanti­omers in the unit cell, with the two conformations being arranged in alternating fashion in the crystal.

The existence of some weak intra­molecular hydrogen bridges (#5, #6) inside the macrocyclic cavity of ***Ctrsp*** might be suspected of being involved in governing its stereochemistry (Fig. 8 ▸, Table 1 ▸). Notwithstanding the distances between the N2 and N4 nitro­gen atoms [2.5572 (18) Å] and between N4 and S3 [3.3218 (13) Å] being rather short, the small N—H⋯*A* angles (113–117°, Table 1 ▸) mitigate against a hydrogen bond of significant strength.

**Figure 9:**
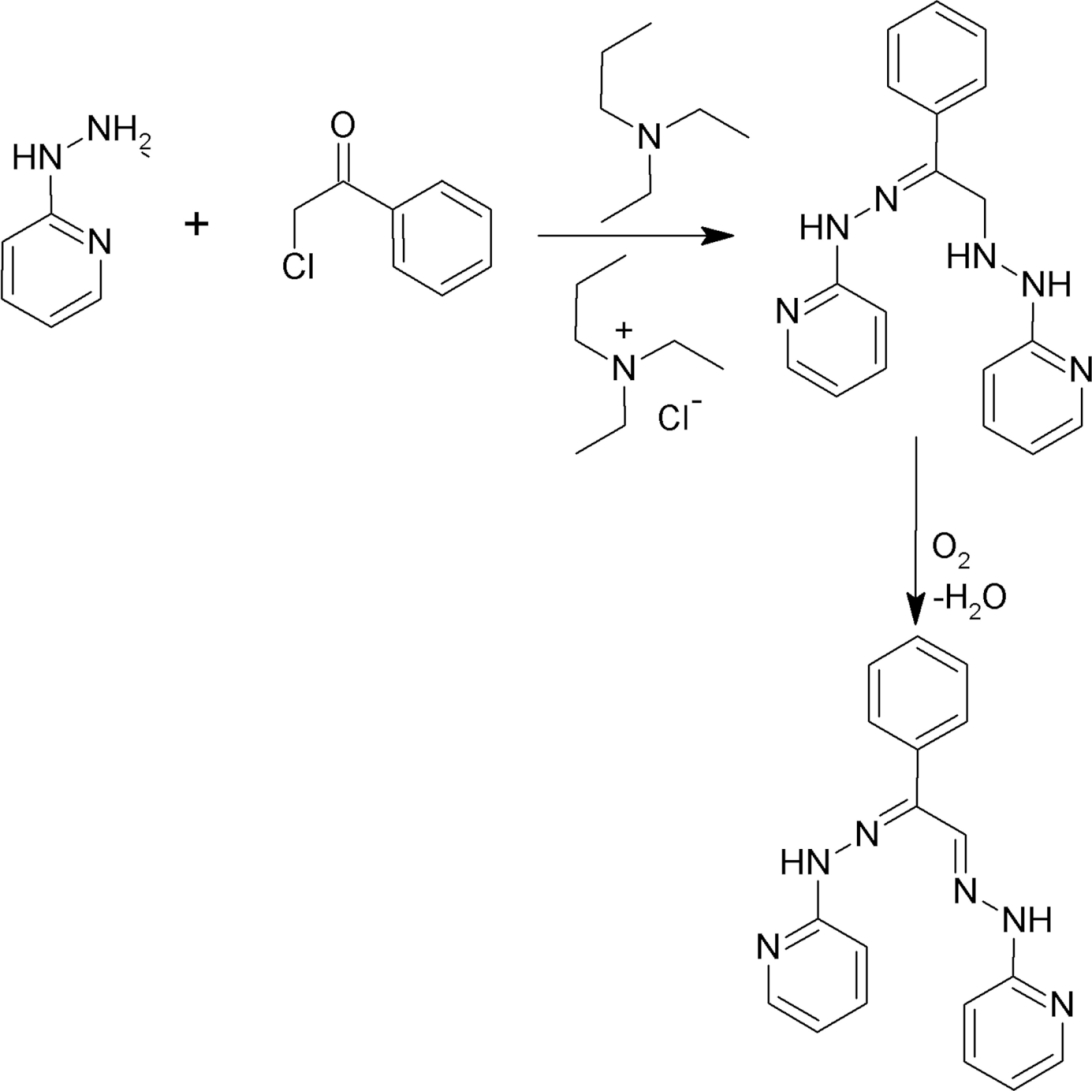
The proposed scheme for ***Dhpk*** formation.

**Figure 10:**
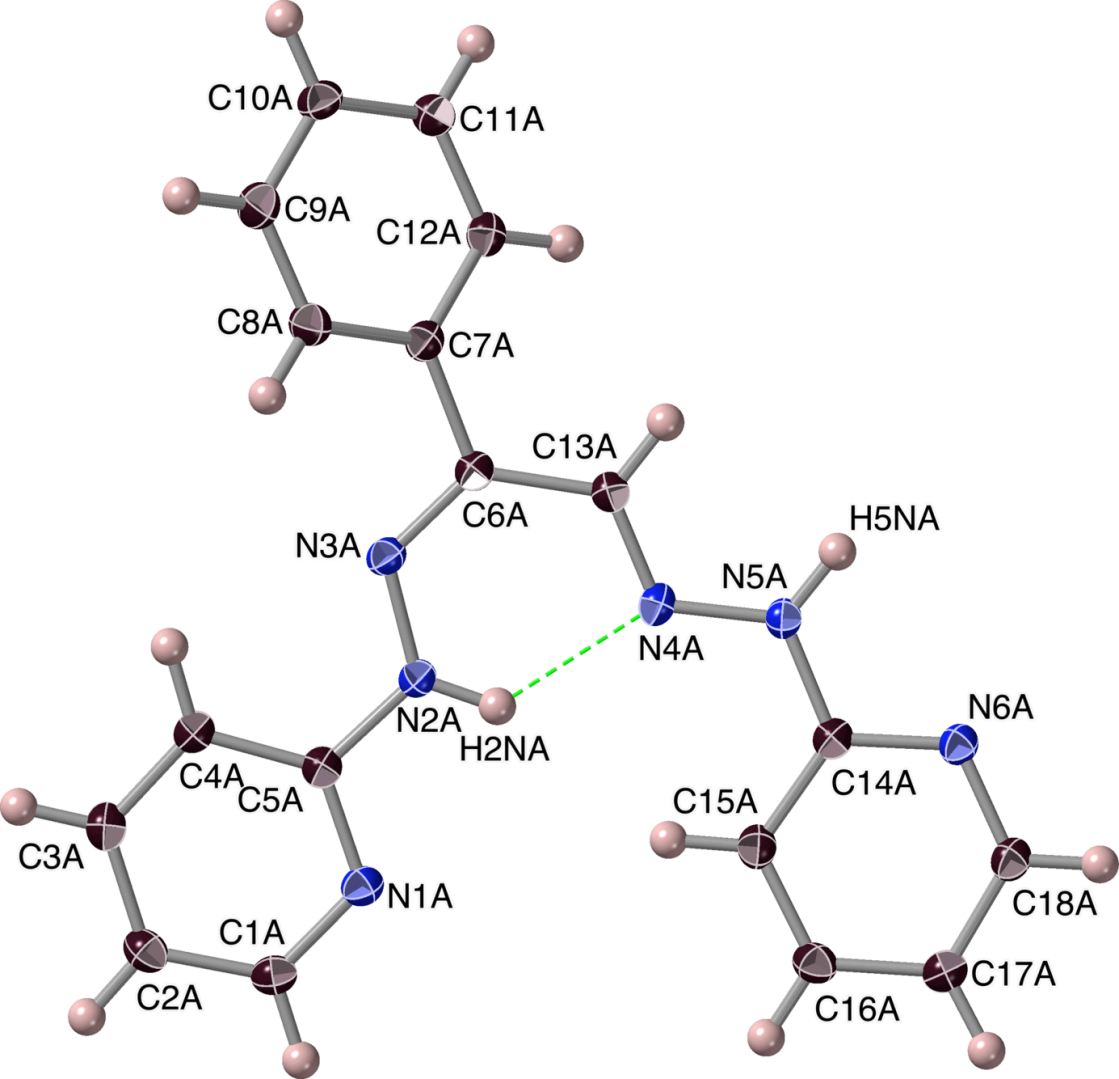
An ellipsoid rendering of an *A*-mol­ecule of ***Dhpk***. Hydrogen atoms are represented as simple spheres, with only those that are specifically discussed being labelled, and displacement ellipsoids are displayed at the 50% probability level.

**Figure 11:**
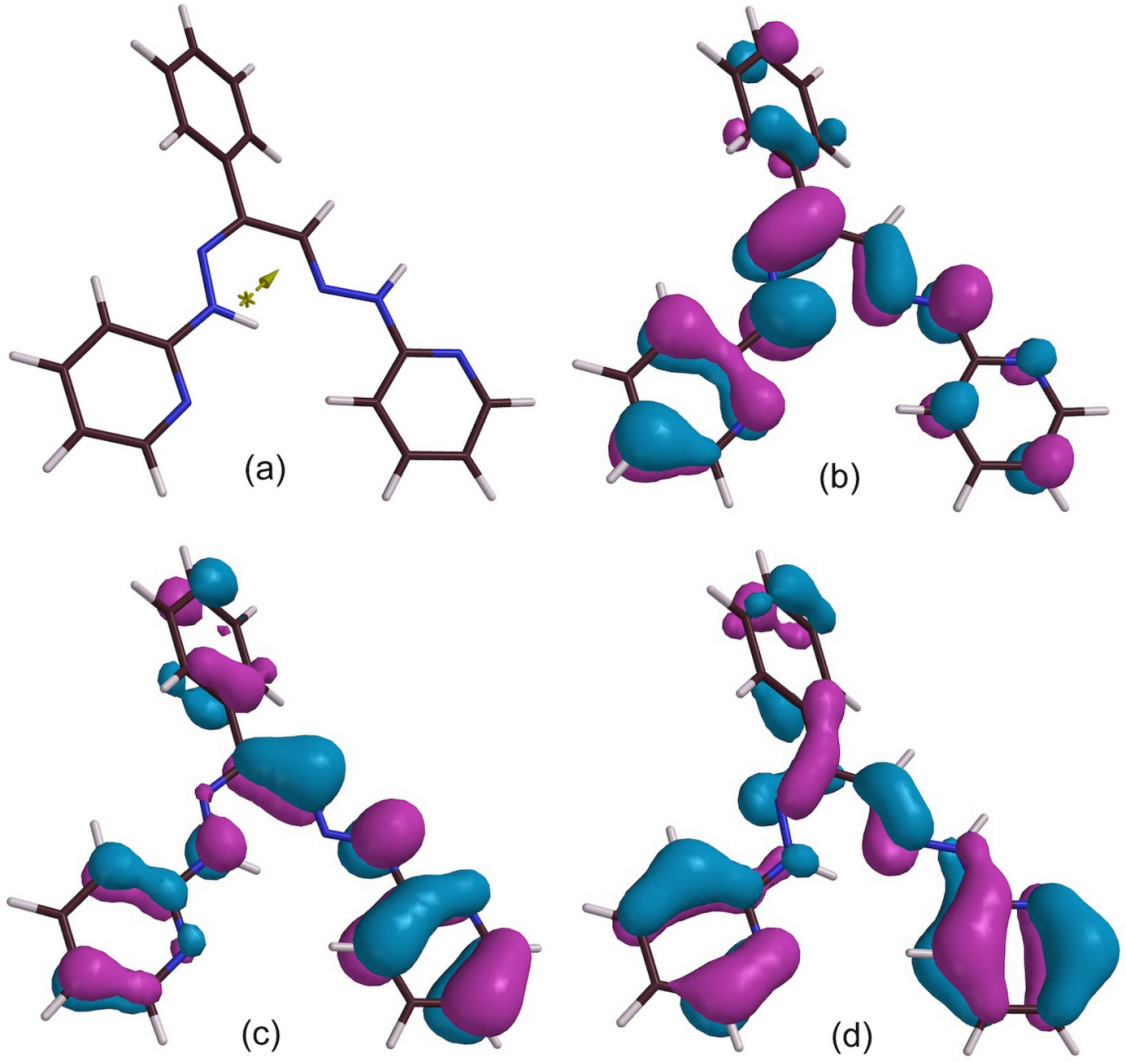
MO wavefunction surfaces (*Spartan-20/24*) for ***Dhpk***: (*a*) reference diagram with the (small) electric dipole moment (0.24 Debye) shown; (*b*) the HOMO, (*c*) the HOMO(–1); (*d*) the HOMO(–7).

**Figure 12:**
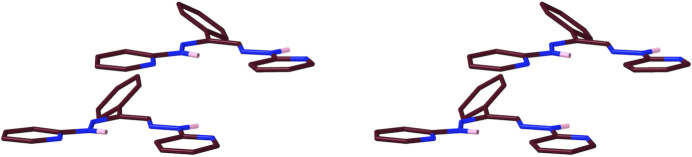
Stick models (inverse stereoview) of a pair conformational enanti­omers of non-adjacent ***Dhpk** A* and *D* mol­ecules. Note the opposite ‘tilts’ of the rear phenyl groups in the upper (*A*-mol­ecule) *vs*. the lower (d-mol­ecule) diagram.

Crystals of ***Dhpk***, 2-[(2*E*)-2-[(2*Z*)-2-phenyl-2-[2-(pyridin-2-yl)hydrazin-1-yl­idene]ethyl­idene]hydrazin-1-yl]pyridine, for diffraction were obtained by (slow thermal) recrystallization of the crude product from *N*-methyl­pyrrolidinone. They crystallized in the monoclinic space group *I*2/*a* (equivalent to *C*2/*c*) with a very large unit cell containing 32 ***Dhpk*** mol­ecules, of four inequivalent structures (*A*, *B*, *C* and *D*) and hence 16 of each conformational enanti­omer. ***Dhpk*** was obtained unexpectedly in a low yield as a by-product in attempts to synthesize ***Mtph*** from ***Mtdp*** according to Fig. 1 ▸, and its formation might be attributable to an alternative pathway of the expected reaction (Fig. 9 ▸). A ***Dhpk*** mol­ecule is shown in Fig. 10 ▸. DFT calculations confirm that associated with its planarity, the ***Dhpk*** mol­ecule has substantial π-bonding conjugation extending from C5 through C14 (Figs. 11 ▸, S6). Components of the MO’s contributing to the completion of this π-bonding pathway are depicted in Fig. 11 ▸, while Fig. 12 ▸ shows two conformational enanti­omers for ***Dhpk***.

Intra­molecular hydrogen bonding between H2*N* and N4 was found in all conformers of ***Dhpk***, with average N2⋯N4 distances of 2.67 Å (Table 1 ▸, #7). Despite the marginally favourable 128– 130° N2—H2*N*⋯N4 angles, the 2.0–2.03 Å H2*N*⋯N4 distances are substanti­ally less than the *ca*. 2.67 Å values for a van der Waals (N)H⋯N contact. By the formation of this bridge, a structurally favourable six-membered ring N2–N3–C6–C13–N4–H2*N* is set. This N—H⋯N inter­action would circumvent any possible C—H⋯N inter­actions.

## 3. Supra­molecular features

The unit cell of ***Dtdpe*** contains two mol­ecules which are pairwise inter­acting *via* putative C2—H2*A*⋯O1(2 − *x*, 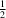 + *y*, 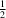 − *z*) hydrogen bridges (Table 2 ▸, Fig. S8). Beyond the edges of the unit cell, each ***Dtdpe*** mol­ecule is connected with four others through such inter­actions, with two from inside and two from outside the unit cell. For the crystal packing of the ***Dtdpe*** mol­ecules, the phenyl groups are parallel to each other (Figs. S7, S8), with C5 near C7 of a neighbouring mol­ecule, so that the phenyl C5 is against the C7 atom of the carbonyl group of an adjacent mol­ecule at 3.359 (3) Å. Phen­yl–phenyl face-to-face inter­actions are otherwise not significant at 4.54 Å. Phenyl carbon atom C2 is 3.365 (2) Å from oxygen atom O1 belonging to another ***Dtdpe*** mol­ecule, so that there is an even weaker C2—H2*A*⋯O1(2 − *x*, 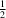 + *y*, 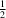 − *z*) inter­action with an H⋯O distance of 2.462 Å.

As it is clear that inter­atomic distances are not complete criteria for the presence of hydrogen bonding (as designated by *Mercury* or *checkCIF*), we addressed the question of the energetics of these inter­actions *via* calculations. Comparison of a hydrogen-bridged dimer with its monomers may reveal any energy advantage (or disadvantage) that might be attributed to such a hydrogen bridge. The DFT calculations (Table 3 ▸, S1) indicate that at an idealized *D*⋯*A* (C⋯O) distance of ∼3.49 Å, this (#8, Table 2 ▸) hydrogen bridge is stabilized by *ca*. 6.8 kJ mol^−1^. The crystallographic *θ* angle (163.8°) for C2—H2*A*⋯O1*B*(2 − *x*, 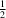 + *y*, 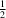 − *z*) is close to appropriate by a prior criterion (Desiraju, 1991 ▸). In summarizing the features of the organization of ***Dtdpe*** mol­ecules in the crystal, one may conclude that it is governed by three weak hydrogen—O1 bridges, two intra­molecular and one inter­molecular inter­actions, which leads to creation of a supra­molecular net consisting of ***Dtdpe*** mol­ecules.

**Figure 13:**
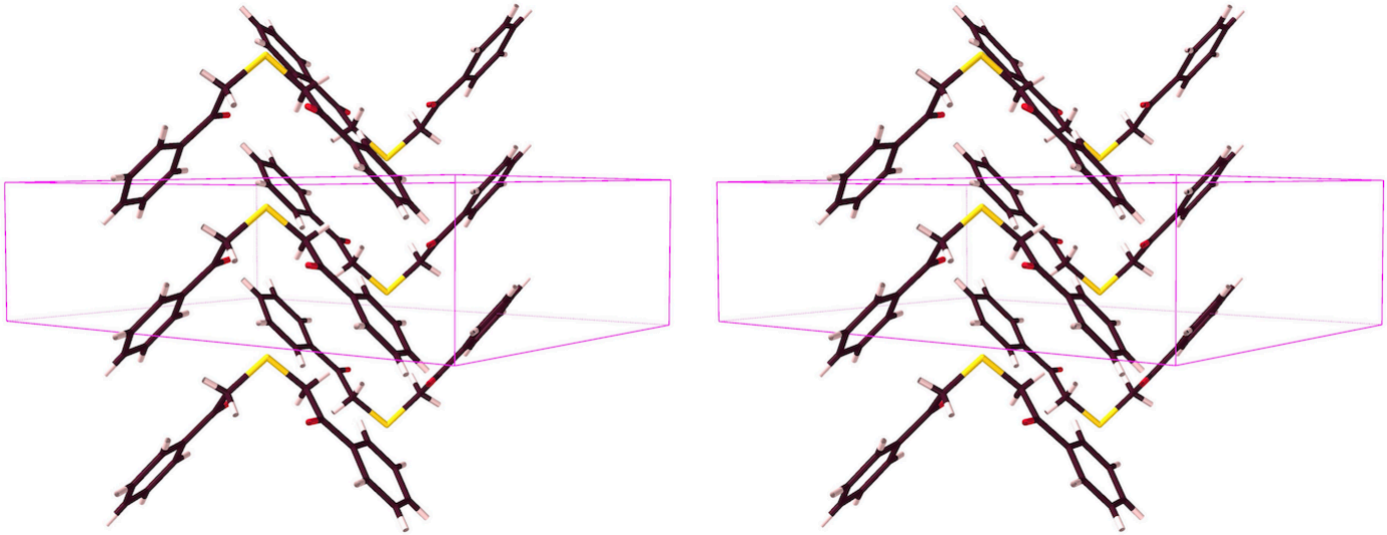
The chevron arrangement of the ***Mtdp*** mol­ecules (stick structure, inverse stereoview).

For the mono­thio-diketone ***Mtdp***, 2-[(2-oxo-2-phenyl­eth­yl)sulfan­yl]-1-phenyl­ethan-1-one, only weak hydrogen-bonding inter­actions are apparent: there are some close π-approaches between mol­ecules, such as a 3.339 (2) Å inter­action of phenyl-C5 with a neighbouring mol­ecule’s carbonyl-C7. Although the separation between C3 and O1(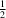 + *x*, 2 − *y*, *z*) of adjacent ***Mtdp*** mol­ecules is only 3.382 (2) Å, the presumed O⋯H distance of 2.769 Å is too long for an appreciable hydrogen bond. In general, the separations between adjacent ***Mtdp*** mol­ecules are too great for one to suspect the existence of any inter­molecular O1⋯H bridges or wider π—π inter­actions between them in the crystal. The 100.2° bond angle at sulfur is correlated with the 79.4° intra­molecular angle between phenyl planes, and the complementary inter­molecular inter­planar angles are hence 0°. These essentially orthogonal relationships amongst phenyl groups are apparent in Fig. 13 ▸, along with the mol­ecules being arranged as alternating anti­parallel sets of chevrons stacked along the *b*-axis direction (Fig. 13 ▸).

**Figure 14:**
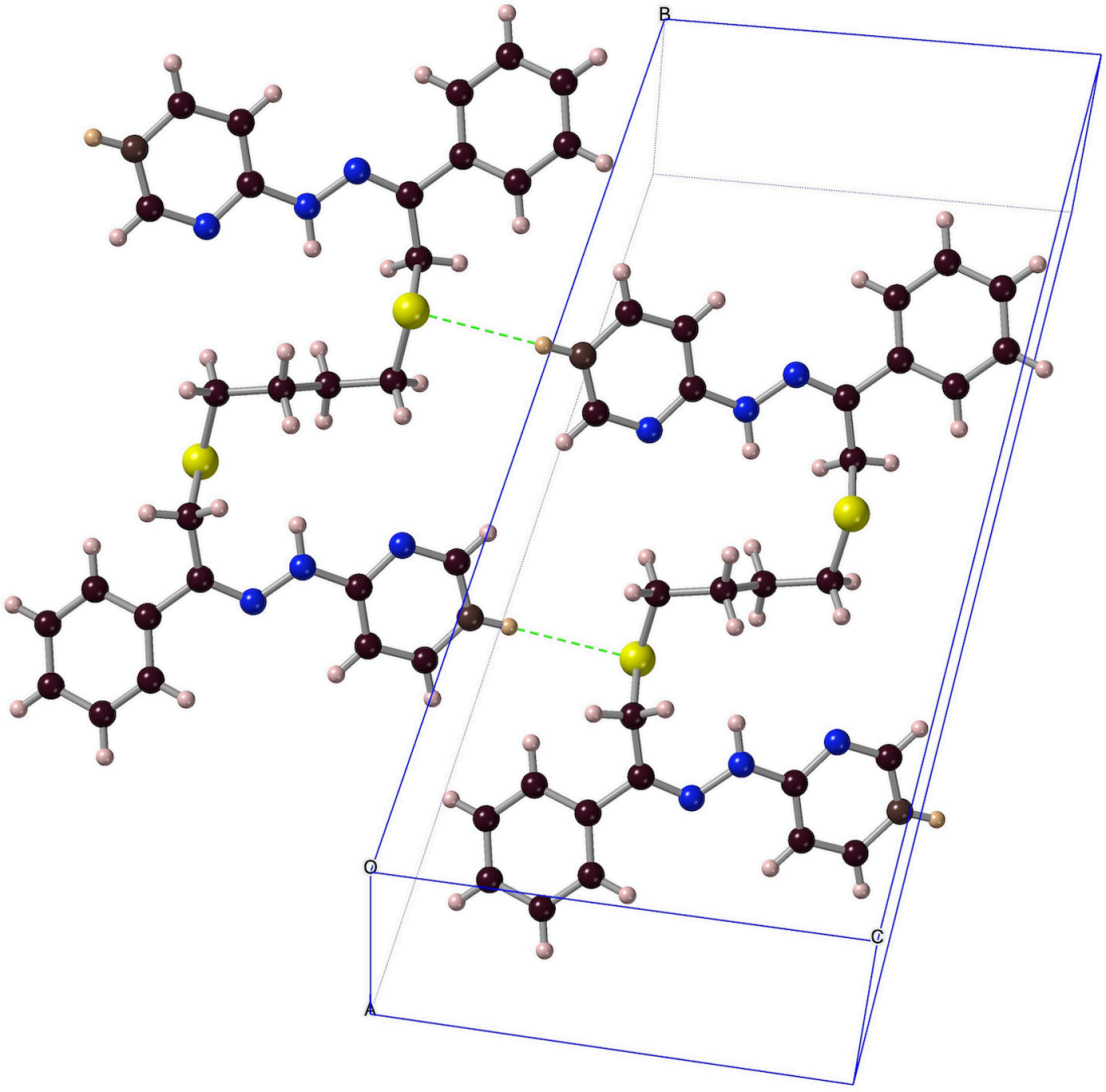
C2—H2*B*⋯S1(*x* − 1, *y*, *z* − 1) pairing of ***Prpsb*** mol­ecules in the crystal (ball-and-stick model).

For the di­thio­ether-dihydrazone ***Prpsb***, no π-stacking is apparent. However, there are inter­molecular C2—H2B⋯S1(*x* − 1, *y*, *z* − 1) contacts that are potential hydrogen bridges. Indeed, these are associated with symmetric pairing in the crystal (Fig. 14 ▸). Pairings of the ***Prpsb*** mol­ecules occurs *via* pyridyl *β*-H inter­actions C2—H2*B*⋯S1(*x* − 1, *y*, *z* − 1) (#10, Table 2 ▸). This pairing or dimerization occurs at the *ab* inter­face of the unit cell (Fig. 14 ▸), and each member of the mol­ecular pair extends above and below an *ab* face. In a ω97X-D/6-31G* DFT model (Table 3 ▸, S1) of this ***Prpsb*** dimer, it has a dubiously large energy advantage (39 kJ mol^−1^) over the monomers; the C⋯S and H⋯S distances are slightly less than in the crystallographic case. The inter­action angle is essentially 170° in both instances, which is favourable for an attractive inter­action. These lines of evidence do therefore suggest a significant hydrogen-bonding inter­action.

**Figure 15:**
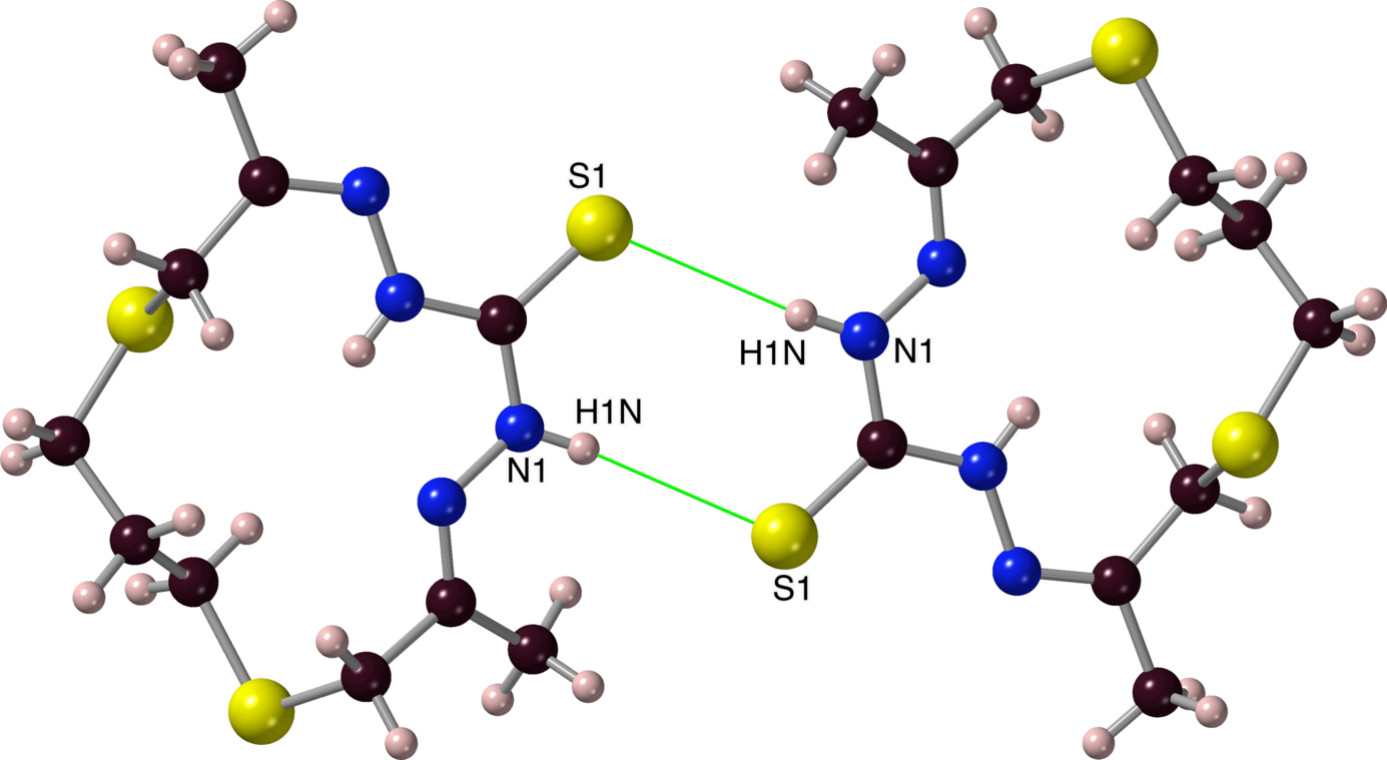
The relationship that casts laterally adjacent ***Ctrsp*** mol­ecules as a centrosymmetric pair, showing the N1—H*N*1⋯S1(−*x* + 1, −*y*, −*z* + 1) hydrogen bonds (ball-and-stick model).

Meanwhile in ***Ctrsp***, unlike enanti­omeric forms are connected pairwise with what present themselves as two S1(−*x* + 1, −*y*, −*z* + 1)⋯H*N*1 inter­actions (#11, Table 2 ▸), with H⋯S1^*i*^ distances of 2.73 (2) Å (Fig. 15 ▸), which is less than the sum (3.0 Å) of the van der Waals contact radii for S and H, and close to the inter­molecular H⋯S distances (2.537–2.739 Å) found in the thio­benzamide extended network (Rigane *et al.*, 2016 ▸). If the N1⋯S1(*x* + 1, −*y*, −*z* + 1) distance of 3.5178 (14) Å is a more reliable indicator for the possible formation of this bridge, then it does suggest an N—H⋯S hydrogen bridge. Although this is somewhat greater than the average hydrogen-bonded N⋯S distance (3.41–3.42 Å) seen for most thio­amides (Desiraju & Steiner, 2001 ▸), it is close to the 3.521 Å value observed in the thio­benzamide supra­molecular network (Rigane *et al.*, 2016 ▸). It is also significantly smaller than the sum of the appropriate covalent and van der Waals radii of H, N and S (4.12 Å). So, this approach indeed suggests the formation of N1—H*N*1⋯S1(−*x* + 1, −*y*, −*z* + 1) hydrogen bridges. In addition, DFT calculations (Table 3 ▸, S1) indicate stabilization of a hydrogen-bonded dimer by 30–40 kJ mol^−1^. It might be noted that the otherwise somewhat unfavourable eight-membered C/N/H/S ring formed (Fig. 15 ▸) is rather planar (SSE = 0.20 Å^2^).

The dipole moment of this mol­ecule (6.65 Debye *in vacuo*; Fig. S13) substanti­ally opposes such a pairwise alignment, so we conclude that the inter­molecular N—H⋯S inter­action at least matches this electrostatic repulsion, and allows pairwise alignment. The ***Ctrsp*** mol­ecules are arranged as double layers (Fig. S14), with each side of the double layer inter­facing the next one *via* its dihydrazone segments. Atoms N2 and C9 of neighbouring mol­ecules are packed closely [3.288 (2) Å], as are the nitro­gen atoms N4 of adjacent mol­ecules [3.583 (2) Å]. Meanwhile, close inter­molecular C6—H6*A*⋯S2(−*x*, *y* + 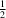, −*z* + 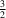) contacts (Fig. S15, Table 3) suffuse the crystal within the layers, though no association energy could be estimated (Table S1).

**Figure 16:**
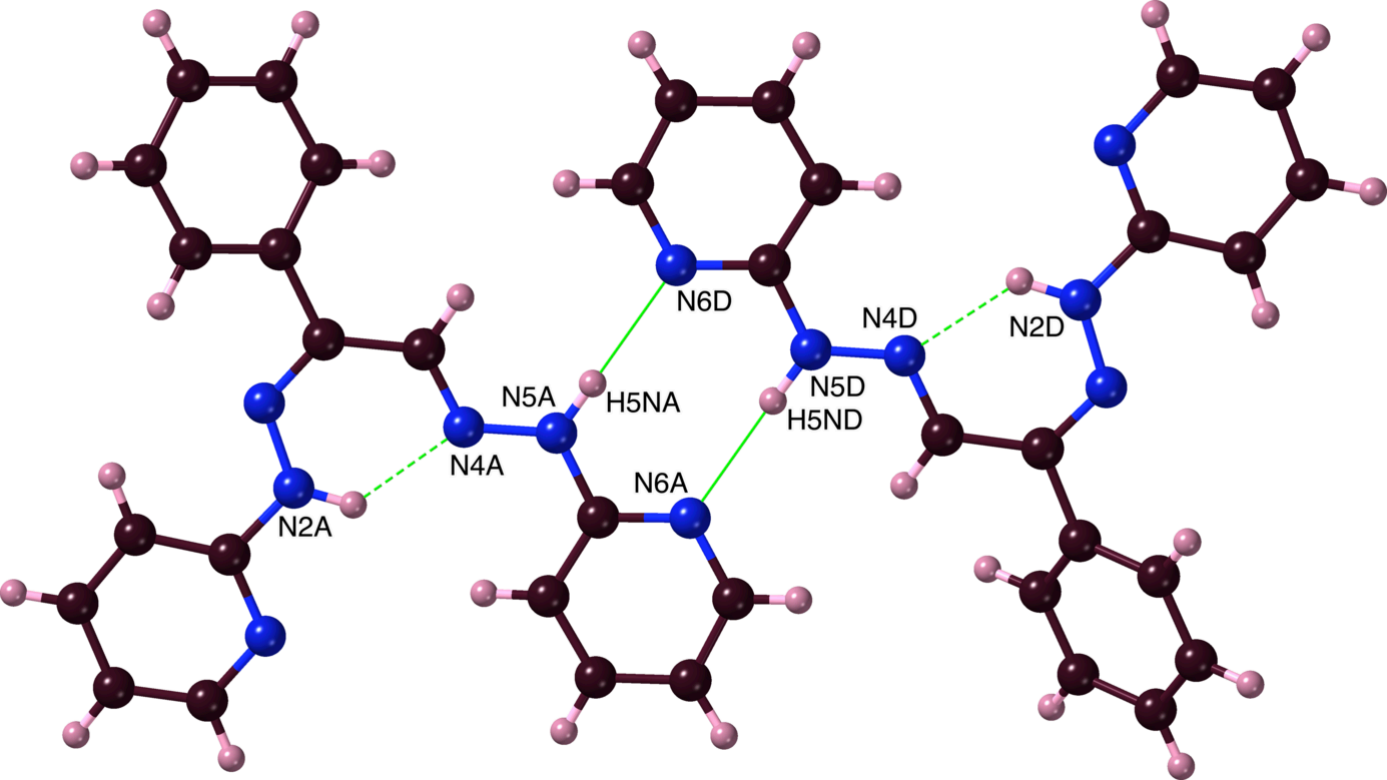
A depiction (ball-and-stick) of the hydrogen bridging present in ***Dhpk***, showing the hydrogen bonds (solid lines) that form a pseudo-centrosymmetric *A—D* dimer and the weaker (lower angle value) intra­molecular hydrogen bonds (dashed lines).

In ***Dhpk:***, the observed *inter*mol­ecular N5⋯N6 distances, essentially 3.04 Å, are much smaller than the 3.67 Å van der Waals contact sums, and both their average H5*N*⋯N6 distances (2.121 Å) and N5—H5*N*⋯N6 angles (*ca*. 176°) also betoken hydrogen bonding (Table 3 ▸, #13). Four inequivalent mol­ecules *A*, *B*, *C* and *D* of ***Dhpk*** are thusly connected as *A–D* and *B–C* pairs by inter­molecular hydrogen bonds with similar geometrical metrics, forming pseudo**-**centrosymmetric couples of like enanti­omers (Fig. 16 ▸, Table 3 ▸). DFT calculations indicate substantial stabilization (Table 3 ▸) of the dimer *vs* the monomers, with an hydrogen-bond order of *ca*. 0.08. The further agglomeration of these ***Dhkp*** pairs results in the formation of a large achiral unit cell containing 32 mol­ecules (Fig. S16). Corresponding pairs of ***Dhpk*** are organized in parallel strata in the crystal (Figs. S15, S17). The pyridines of any given dimer are essentially coplanar, but the inter­planar angles between pyridine moieties of different dimers are near 90°.

## 4. Synthesis and crystallization

The thio­ether-dihydrazone compounds were prepared by a multi-step procedure (Fig. 1 ▸). Their thio­ether-diketone precursors were prepared *via* the ‘TACO’ method of Goldcamp *et al.* (2000 ▸) employing chloro­acetone or phenacyl chloride (2-chloro­aceto­phenone), *via* reaction of *N,N,N*-tri­ethyl-*N*-(propan-2-on­yl)ammonium or *N,N,N*-triethyl-*N*-(phenyl­ethan-2-on­yl)ammonium chloride salt with various thiols. The chloride inter­mediates were prepared by the addition of Et_3_N to the chloro­acetone or phenacyl chloride.

**1,8-Diphenyl-3,6-di­thia­octane-1,8-dione (*****Dtdpe*****).** Di­thia­diphenacyl derivatives were prepared in a similar manner to their acetyl analogues (Pavlishchuk *et al.*, 2024 ▸). As a typical synthesis, 1,2-ethane­dithiol (6.00 mL, 71.5 mol) was treated with NaBH_4_ (2.74 g, 72.4 mmol) in EtOH under N_2_ to reduce any di­sulfides. The *N,N,N*-triethyl-*N*-(phenyl­ethan-2-on­yl)ammonium chloride inter­mediate was prepared by adding Et_3_N (14.9 g, 147 mmol) in 30 mL of MeOH to 2-chloro­aceto­phenone (22.1 g, 143 mol) in 100 mL Et_2_O dropwise over 15 minutes with constant stirring. The solution was allowed to stir for an additional 15 minutes and flushed with N_2_, followed by the addition of the treated di­thiol over 15 minutes. Some of the Et_3_NHCl by-product precipitated and was removed by gravity filtration. The remaining solvent was removed *via* rotary evaporation to afford the white solid product contaminated with the remaining Et_3_NHCl salt. The reaction mixture was stirred with a portion of Et_2_O for 1 h to separate the two components. The Et_3_NHCl was removed through gravity filtration, and the filtrate was refrigerated overnight, after which any precipitated Et_3_NHCl was filtered off and the remaining Et_2_O removed *via* rotary evaporation, yielding a fluffy white solid. A sample was recrystallized from MeOH (charcoal), to gradually yield diffraction-quality crystals. Yield: 8.71 g (37%). CI-MS: 541 ([*M*+*M*′]^+^, 2%), 509 ([*M*+*M*′′]^+^, 30%), 330 ([*M*]^+^, 64%), 211 ([*M*′]^+^, 8%), 179 ([*M*′′]^+^, 100%). Fragmentation designations are given in the ESI. ^1^H NMR (DMSO-*d*_6_): δ 2.50 (*t*, 4H), 3.33 (*s*, 4H), 7.53 (*m*, 4H), 7.64 (*m*, 2H), 7.98 (*m*, 4H).

**1,5-Diphenyl-3-thia­pentane-1,5-dione (*****Mtdp*****)** was prepared following a procedure published by Cuthbertson *et al.* (1975 ▸). Though many preparative methods for this compound can be found in the literature, this particular synthesis is quite straightforward and produced well-formed colourless plates in high yield: 18.2 g (90%). CI-MS: 271 ([*M*+H]^+^, 100%), 165 ([*M*′]^+^, 21%), 151 ([*M*′′]^+^, 29%). ^1^H NMR (DMSO-*d*_6_): δ 4.13 (*s*, 4H), 7.53 (*m*, 4H), 7.66 (*m*, 2H) 7.98 (*m*, 4H).

**1,10-Diphenyl-3,8-di­thia­decane-1,10-dione (*****Dtdpb*****)** was prepared in the same manner as ***Dtdkb*** and ***Dtdpp*** (Pavlishchuk *et al.*, 2024 ▸) using 1,4-butane­dithiol (12.0 mL, 94.2 mmol), NaBH_4_ (3.45 g, 91.2 mmol), 2-chloro­aceto­phenone (29.1 g, 188 mmol), and Et_3_N (20.0 g, 198 mmol). The white solid produced was characterized and used without further purification. Yield: 27.8 g (82%). Crude CI-MS: 358 ([*M*]^+^, 52%), 239 ([*M*′]^+^, 75%), 207 ([*M*′′]^+^, 100%). ^1^H NMR (DMSO-*d*_6_): δ 1.62 (*q*, 4H), 2.53 (*t*, 4H), 3.76 (*s*, 4H), 7.47 (*m*, 6H), 7.93 (*m*, 4H)].

**1,10-Bis(2**’**-pyridyl­hydrazon­yl)-1,10-diphenyl-3,8-di­thia­dec­ane (*****Prpsb*****).** A methanol (20 mL) solution of 1.81 g (5.06 mmol) ***Dtdpb*** (Pavlishchuk *et al.*, 2024 ▸) and 1.12 g (10.3 mmol) 2-hydrazino­pyridine was refluxed overnight. The reaction mixture was allowed to cool to room temperature, and a yellow solid precipitated. The crude solid was collected, then recrystallized from MeOH to afford a yellow powder. Yield: 1.22 g (45%). CI-MS: 541 ([*M*+H]^+^, 23%), 450 ([*M*′′′]^+^, 25%), 332 ([*M*′]^+^, 26%), 196 ([P*M*′]^+^, 100%). ^1^H NMR (DMSO-*d*_6_): δ 2.47 (*t*, 4H), 3.35 (*t*, 4H), 4.02 (*s*, 4H), 6.82 (*m*, 2H), 7.57 (*m*, 14H), 8.15 (*m*, 2H), 10.01 (*s*, 2H). A sample subsequently recrystallized from ethanol (charcoaled) yielded golden yellow crystals suitable for diffraction. Analysis: calculated % C, 66.6; H, 5.97, N, 15.5; S, 11.9; Found% C, 66.3; H, 5.94, N, 15.2; remainder 12.5.

**The pyridyl­hydrazones of*****Dtdkp*****: (*****Ctrsp*****).** In an attempt to prepare the bis­(thio­carbohydrazone) of the di­thio­ether, 2.67 g (12.1 mmol) of ***Dtdkp*** were added dropwise to a warm methano­lic solution (343 K) of thio­carbohydrazide (2.58 g, 24.3 mmol). The resulting reaction mixture was allowed to reflux for 30 minutes, and then allowed to cool to room temperature. As the solution cooled, an off-white, granular solid precipitated, which was filtered off, and the filtrate concentrated *via* rotary evaporation. Off-white crystals precipitated from the filtrate overnight. Both fractions displayed the same species in their mass spectra. The crude solid proved relatively insoluble, making recrystallization difficult, and proved detrimental to metal complex synthesis. Yield: 3.48 g (72.6%). FAB-MS: 397 ([*M*+H]^+^, 53%), 291 ([*M* − *M*′′′]^+^, 93%). ^1^H NMR (DMSO-*d*_6_): δ 2.51 (*s*, 2H), 3.19 (*s*, 4H), 3.45 (*s*, 4H), 4.11 (*s*, 4H). When a sample was recrystallized from hot 2-meth­oxy­ethanol (with charcoaling), light-yellow, diffraction-quality crystals appeared after several days. Analysis: calculated % C, 41.4; H, 6.25, N, 19.3; S, 33.1; Found% C, 41.5; H, 6.19, N, 19.1; remainder 33.2.

**1,5-Diphenyl-1,5-bis­(2-pyridyl­hydrazon­yl)-3-thia­penta­ne** and **2-[(2*****E*****)-2-[(2*****Z*****)-2-phenyl-2-[2-(pyridin-2-yl)hydrazin-1-yl­idene]ethyl­idene]hydrazin-1-yl]pyridine (*****Dhpk*****).** A methanol (20 mL) solution of 2.70 g (10.0 mmol) ***Mtdp*** and 4.13 g (37.8 mmol) of 2-hydrazino­pyridine was refluxed overnight. The reaction mixture was allowed to cool to room temperature, and a yellow solid precipitated. The crude solid was collected and recrystallized from MeOH to afford a yellow powder. Yield: 3.56 g (78.7%). CI-MS: 453 ([*M*+H]^+^, 2%), 196 ([*M*′′]^+^, 100%). ^1^H NMR (DMSO-*d*_6_): δ 3.32 (*s*, 4H), 6.86 (*m*, 10H), 7.24 (*m*, 2H), 7.70 (*m*, 2H), 8.18 (*m*, 2H), 10.18 (*s*, 2H). Generation of diffraction-quality crystals proved difficult. Finally, an aliquot was recrystallized from hot *N*-methyl­pyrrolidone (with charcoaling), to give a small yield of golden yellow platelets, used for X-ray diffraction. The product obtained was the *a­thio*-dihydrazone ***Dhpk***; C, H, N calculated for C_18_H_16_N_6_, 68.3%, 5.10%, 26.6%, Found (recrystallized from DMA), 67.8%, 5.02%, 26.2%.

**Physical Measurements:**

EI-, CI-, APCI-, ESI-, FAB-LSIMS- and FT-mass spectrometries were performed on Thermo Finnigan TSQ70, Thermo-Electron LTQ-FT 7T, VG70SE, Waters AutoSpec Ultima-Q, or Sciex API3000 mass instruments. Proton NMR were obtained on a 300 MHz Varian Unity Inova spectrometer using chloro­form-*d* or dimethyl sulfoxide-*d_6_* as solvent with TMS as inter­nal standard. Elemental microanalyses were performed by Robertson Microlit Laboratories (Madison/Ledgewood, NJ).

Structure diagrams were generated using *CrystalMaker*-*10/11* (Palmer *et al.*, 2024 ▸), *Mercury* 2022/2023 (Macrae *et al.*, 2006 ▸), *Preview-11* (Apple, Inc. 2024 ▸) and *Photoshop 7* (Knoll *et al.*, 2002 ▸).

The online engine *publCIF* (Westrip, 2010 ▸) was used to generate tables of geometric parameters. MO wavefunction and energy calculations employed the *Spartan-20* and *Spartan-24* software (Deppmeier *et al.*, 2024 ▸) on an iMac21,1. Initially, a geometry minimization was performed using a ω97X-D/6-31G* model, followed by a ω97X-D/6-31G* energy minimization. Subsequent calculations used a B3LYP/6-311+G** energy minimization, preceded by a B3LYP/6-311+G** geometry minimization where practical. For probing the potential inter­molecular hydrogen bonding, the ‘monomer’ was structurally minimized and the energy of the structure then calculated. The procedure was then repeated on the mol­ecular pair with the suspected hydrogen bond(s). Table S1 shows some additional results. Additional supplementary materials are available at https://researchdiscovery.drexel.edu/esploro/outputs/dataset/Some-thioether-ketones-and-their-related-derivatives/991021955715504721?institution=01DRXU_INST (https://doi.org/10.17918/00010914).

## 5. Refinement

Crystal data, data collection and structure refinement details are summarized in Table 4 ▸. C-bound H atoms were positioned with idealized geometry, and refined using a riding model.

## Supplementary Material

Crystal structure: contains datablock(s) Dtdpe, Mtdp, Prpsb, Ctrsp, Dhpk. DOI: 10.1107/S2056989025004037/jy2055sup1.cif

Supporting information file. DOI: 10.1107/S2056989025004037/jy2055Dtdpesup12.cml

Structure factors: contains datablock(s) Dtdpe. DOI: 10.1107/S2056989025004037/jy2055Dtdpesup13.hkl

Supporting information file. DOI: 10.1107/S2056989025004037/jy2055Dtdpesup7.cdx

Supporting information file. DOI: 10.1107/S2056989025004037/jy2055Mtdpsup13.cml

Structure factors: contains datablock(s) Mtdp. DOI: 10.1107/S2056989025004037/jy2055Mtdpsup14.hkl

Supporting information file. DOI: 10.1107/S2056989025004037/jy2055Mtdpsup8.cdx

Supporting information file. DOI: 10.1107/S2056989025004037/jy2055Prpsbsup14.cml

Structure factors: contains datablock(s) Prpsb. DOI: 10.1107/S2056989025004037/jy2055Prpsbsup15.hkl

Supporting information file. DOI: 10.1107/S2056989025004037/jy2055Prpsbsup9.cdx

Supporting information file. DOI: 10.1107/S2056989025004037/jy2055Ctrspsup10.cdx

Supporting information file. DOI: 10.1107/S2056989025004037/jy2055Ctrspsup15.cml

Structure factors: contains datablock(s) Ctrsp. DOI: 10.1107/S2056989025004037/jy2055Ctrspsup16.hkl

Supporting information file. DOI: 10.1107/S2056989025004037/jy2055Dhpksup11.cdx

Supporting information file. DOI: 10.1107/S2056989025004037/jy2055Dhpksup16.cml

Structure factors: contains datablock(s) Dhpk. DOI: 10.1107/S2056989025004037/jy2055Dhpksup17.hkl

Supporting information file. DOI: 10.1107/S2056989025004037/jy2055sup18.docx

CCDC references: 2409931, 2409928, 2409933, 2409954, 2409932

Additional supporting information:  crystallographic information; 3D view; checkCIF report

## Figures and Tables

**Table 1:** Intra­molecular hydrogen-bond metrics (Å, °) for the compounds

Bridge number	Compound	Bridge	*d*(*D*—H)	*d*(H⋯*A*)	*d*(*D*⋯*A*)	*D*—H⋯*A*
1	**Dtdpe**	C9—H9*B*⋯O1	0.97	2.61	3.229 (3)	122.1
2	**Mtdp**	C8—H8*C*⋯O1^i^	0.99	2.54	3.1981 (16)	123.9
3	**Mtdp**	C6—H6*A*⋯O1	0.95	2.512	2.807 (2)	98.03
4	**Prpsb**	N2—H2*N*⋯S1	0.94 (3)	2.73 (3)	3.3599 (15)	126 (2)
5	**Ctrsp**	N4—H4N⋯S3	0.83 (2)	2.864 (19)	3.3218 (13)	116.9 (15)
6	**Ctrsp**	N4—H4N⋯N2	0.83 (2)	2.11 (2)	2.5572 (18)	113.5 (16)
7*A*	**Dhpk**	N2*A*—H2*NA*⋯N4*A*	0.897 (17)	2.018 (16)	2.6687 (14)	128.3 (14)
7*B*	**Dhpk**	N2*B*—H2*NB*⋯N4*B*	0.906 (17)	1.995 (16)	2.6742 (13)	130.6 (14)
7*C*	**Dhpk**	N2*C*—H2*NC*⋯N4*C*	0.877 (17)	2.026 (16)	2.6784 (14)	130.4 (15)
7*D*	**Dhpk**	N2*D*—H2*ND*⋯N4*D*	0.911 (15)	2.008 (15)	2.6810 (13)	129.5 (12)

Symmetry code: (i) −*x* + 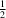, *y*, −*z* + 1.

**Table 2:** Potential inter­molecular hydrogen bridges (Å, °)

Bridge number	Compound	Bridge	*d*(*D*—H)	*d*(H⋯*A*)	*d*(*D*⋯*A*)	*D*—H⋯*A*
8	**Dtdpe**	C2—H2*A*⋯O1^i^	0.93	2.46	3.365 (2)	164
9	**Mtdp**	C3—H3*A*⋯O1^ii^	0.95	2.77	3.382 (2)	123
10	**Prpsb**	C2—H2*B*⋯S1^iii^	0.95	3.02	3.9489 (18)	167
11	**Ctrsp**	N1—H1*N*⋯S1^iv^	0.83 (2)	2.73 (2)	3.5178 (14)	160.4 (19)
12	**Ctrsp**	C6—H6*A*⋯S2^v^	0.97	2.94	3.7524 (17)	142
13*A*	**Dhpk**	N5*A*—H5*NA*⋯N6*D*	0.916 (14)	2.126 (14)	3.0401 (14)	175.9 (14)
13*B*	**Dhpk**	N5*B*—H5*NB*⋯N6*C*	0.913 (15)	2.126 (14)	3.0371 (14)	175.4 (12)
13*C*	**Dhpk**	N5*C*—H5*NC*⋯N6*B*	0.911 (14)	2.129 (14)	3.0390 (14)	177.0 (14)
13*D*	**Dhpk**	N5*D*—H5*ND*⋯N6*A*	0.938 (14)	2.102 (14)	3.0388 (14)	177.5 (10)

Symmetry codes: (i) 2 − *x*, 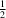 + *y*, 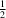 − *z*; (ii) 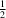 + *x*, 2 − *y*, *z*; (iii) *x* − 1, *y*, *z* − 1; (iv) −*x* + 1, −*y*, −*z* + 1; (v) −*x*, *y* + 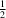, −*z* + 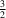.

**Table 3:** Outcomes from DFT calculations on inter­molecular hydrogen bonds

Compound and bridge type	Energy value^*a*^ (*E*_h_)	Dimer energy advantage (kJ)	H⋯*A* bond order	*d*(*D*—H), Å	*d*(H⋯*A*), Å	*d*(*D*⋯*A*), Å	*D*—H⋯*A*
**Ctrsp** dimer, 11	−3611.363181	38.6	0.3	1.03	1.40	3.41	168
**Ctrsp** monomer^*b*^	−1805.674235			1.01			
**Dtdpe** dimer^*c*^, 8	−3287.732002	6.8	0.03	1.08	2.41	3.49	147
**Dtdpe** monomer	−1643.864709			1.08			
**Mtdp** monomer	−1167.000642			1.09			
**Mtdp** dimer, 9	−2334.005015	9.8	^*d*^	1.22	2.76^*e*^	3.48^*e*^	133^*e*^
**Prpsb** monomer	−2286.795468			1.08			
**Prpsb** dimer^*c*^	−4573.605938	39.4	0.04	1.09	2.74	3.82	170
**Dhpk** dimer, 13*A*	−2048.565734	42.3	0.08	1.03	2.05e	3.08e	177
**Dhpk** monomer	−1024.274809			1.02			

Notes: (*a*) Intra­molecular hydrogen bonds are not specified during the calculations; (*b*) B3LYP/6–311+G** geometry minimization for a CH—S dimer gave an unrealistic geometry, although an estimate was made using ωB97X-D/6–31G*; (*c*) B3LYP/6–311+G** geometry minimization failed to converge for **Dtdpe** or **Prpsb**, although an estimate was made using ωB97X-D/6–31G*; (*d*) not calculated; (*e*) mean of two values.

**Table 4:** Experimental details

	**Dtdpe**	**Mtdp**	**Prpsb**	**Ctrsp**	**Dhpk**
Crystal data					
Chemical formula	C_18_H_18_O_2_S_2_	C_16_H_14_O_2_S	C_30_H_32_N_6_S_2_	C_10_H_18_N_4_S_3_	C_18_H_16_N_6_
*M*_r_	330.44	270.33	540.73	290.46	316.37
Crystal system, space group	Monoclinic, *P*2_1_/*c*	Monoclinic, *I*2/*a*	Monoclinic, *P*2_1_/*n*	Monoclinic, *P*2_1_/*c*	Monoclinic, *I*2/*a*
Temperature (K)	293	100	102	293	100
*a*, *b*, *c* (Å)	5.1639 (2), 11.1078 (3), 14.8132 (5)	13.1307 (3), 5.0945 (1), 19.2847 (5)	5.3442 (4), 24.9746 (18), 10.3552 (7)	9.1219 (3), 8.6565 (2), 17.7935 (7)	34.4957 (2), 10.4262 (1), 37.9992 (3)
β (°)	92.868 (4)	93.167 (2)	99.789 (3)	92.195 (3)	115.604 (1)
*V* (Å^3^)	848.61 (5)	1288.07 (5)	1361.98 (17)	1404.01 (8)	12324.7 (2)
*Z*	2	4	2	4	32
Radiation type	Cu *K*α	Cu *K*α	Mo *K*α	Cu *K*α	Cu *K*α
μ (mm^−1^)	2.87	2.18	0.23	4.70	0.69
Crystal size (mm)	0.35 × 0.20 × 0.07	0.29 × 0.10 × 0.08	0.32 × 0.26 × 0.12	0.40 × 0.34 × 0.20	0.44 × 0.23 × 0.18

Data collection					
Diffractometer	XtaLAB Synergy, Dualflex, HyPix	XtaLAB Synergy, Dualflex, HyPix	Bruker APEXII CCD	XtaLAB Synergy, Dualflex, HyPix	XtaLAB Synergy, Dualflex, HyPix
Absorption correction	Multi-scan (*CrysAlis PRO*; Rigaku OD, 2022 ▸)	Multi-scan (*CrysAlis PRO*; Rigaku OD, 2022 ▸)	Multi-scan (*SADABS*; Krause *et al.*, 2015 ▸)	Multi-scan (*CrysAlis PRO*; Rigaku OD, 2022 ▸)	Multi-scan (*CrysAlis PRO*; Rigaku OD, 2022 ▸)
*T*_min_, *T*_max_	0.609, 1.000	0.520, 1.000	0.354, 0.746	0.683, 1.000	0.582, 1.000
No. of measured, independent and observed [*I* > 2σ(*I*)] reflections	31356, 1802, 1464	6439, 1325, 1253	19288, 4154, 3484	75253, 2983, 2806	219947, 12730, 8570
*R*_int_	0.050	0.040	0.069	0.053	0.068
(sin θ/λ)_max_ (Å^−1^)	0.638	0.636	0.715	0.636	0.630

Refinement					
*R*[*F*^2^ > 2σ(*F*^2^)], *wR*(*F*^2^), *S*	0.046, 0.131, 1.11	0.031, 0.084, 1.10	0.065, 0.178, 1.03	0.031, 0.087, 1.08	0.041, 0.138, 1.04
No. of reflections	1802	1325	4154	2983	12730
No. of parameters	101	87	176	164	897
H-atom treatment	H-atom parameters constrained	H-atom parameters constrained	H atoms treated by a mixture of independent and constrained refinement	H atoms treated by a mixture of independent and constrained refinement	H atoms treated by a mixture of independent and constrained refinement
Δρ_max_, Δρ_min_ (e Å^−3^)	0.31, −0.33	0.31, −0.26	0.90, −0.75	0.22, −0.28	0.20, −0.31

Computer programs: *CrysAlis PRO* (Rigaku OD, 2022 ▸), *APEX2* (Bruker, 2012 ▸), *SHELXT* (Sheldrick, 2015*a* ▸), *SHELXL2019/2* (Sheldrick, 2015*b* ▸) and *SHELXTL* (Sheldrick, 2008 ▸).

## supplementary crystallographic information

### 2-({2-[(2-oxo-2-phenylethyl)sulfanyl]ethyl}sulfanyl)-1-phenylethan-1-one (Dtdpe) Crystal data

**Table:**

C_18_H_18_O_2_S_2_	*F*(000) = 348
*M**_r_* = 330.44	*D*_x_ = 1.293 Mg m^−^^3^
Monoclinic, *P*2_1_/*c*	Cu *K*α radiation, λ = 1.54184 Å
*a* = 5.1639 (2) Å	Cell parameters from 12805 reflections
*b* = 11.1078 (3) Å	θ = 5.0–71.9°
*c* = 14.8132 (5) Å	µ = 2.87 mm^−^^1^
β = 92.868 (4)°	*T* = 293 K
*V* = 848.61 (5) Å^3^	Needle, colorless
*Z* = 2	0.35 × 0.20 × 0.07 mm

### 2-({2-[(2-oxo-2-phenylethyl)sulfanyl]ethyl}sulfanyl)-1-phenylethan-1-one (Dtdpe) Data collection

**Table:**

XtaLAB Synergy, Dualflex, HyPix diffractometer	1802 independent reflections
Radiation source: micro-focus sealed X-ray tube	1464 reflections with *I* > 2σ(*I*)
Detector resolution: 10.0000 pixels mm^-1^	*R*_int_ = 0.050
ω scans	θ_max_ = 79.4°, θ_min_ = 5.0°
Absorption correction: multi-scan (CrysAlisPro; Rigaku OD, 2022)	*h* = −5→6
*T*_min_ = 0.609, *T*_max_ = 1.000	*k* = −14→14
31356 measured reflections	*l* = −18→18

### 2-({2-[(2-oxo-2-phenylethyl)sulfanyl]ethyl}sulfanyl)-1-phenylethan-1-one (Dtdpe) Refinement

**Table:**

Refinement on *F*^2^	Secondary atom site location: difference Fourier map
Least-squares matrix: full	Hydrogen site location: inferred from neighbouring sites
*R*[*F*^2^ > 2σ(*F*^2^)] = 0.046	H-atom parameters constrained
*wR*(*F*^2^) = 0.131	*w* = 1/[σ^2^(*F*_o_^2^) + (0.0519*P*)^2^ + 0.1934*P*] where *P* = (*F*_o_^2^ + 2*F*_c_^2^)/3
*S* = 1.11	(Δ/σ)_max_ < 0.001
1802 reflections	Δρ_max_ = 0.31 e Å^−^^3^
101 parameters	Δρ_min_ = −0.33 e Å^−^^3^
0 restraints	Extinction correction: SHELXL-2019/2 (Sheldrick, 2015b), Fc^*^=kFc[1+0.001xFc^2^λ^3^/sin(2θ)]^-1/4^
Primary atom site location: dual	Extinction coefficient: 0.0069 (17)

### 2-({2-[(2-oxo-2-phenylethyl)sulfanyl]ethyl}sulfanyl)-1-phenylethan-1-one (Dtdpe) Special details

**Table:**

Geometry. All esds (except the esd in the dihedral angle between two l.s. planes) are estimated using the full covariance matrix. The cell esds are taken into account individually in the estimation of esds in distances, angles and torsion angles; correlations between esds in cell parameters are only used when they are defined by crystal symmetry. An approximate (isotropic) treatment of cell esds is used for estimating esds involving l.s. planes.

### 2-({2-[(2-oxo-2-phenylethyl)sulfanyl]ethyl}sulfanyl)-1-phenylethan-1-one (Dtdpe) Fractional atomic coordinates and isotropic or equivalent isotropic displacement parameters (Å^2^)

**Table:**

	*x*	*y*	*z*	*U*_iso_*/*U*_eq_	
S1	0.90030 (15)	0.65689 (5)	0.41185 (4)	0.0972 (3)	
O1	0.9474 (3)	0.40238 (13)	0.28528 (12)	0.0942 (5)	
C1	0.6998 (3)	0.54507 (15)	0.20170 (12)	0.0614 (4)	
C2	0.6625 (4)	0.66376 (17)	0.17434 (17)	0.0782 (6)	
H2A	0.771240	0.724098	0.197704	0.094*	
C3	0.4623 (5)	0.6917 (2)	0.11208 (19)	0.0959 (8)	
H3A	0.437709	0.771073	0.093520	0.115*	
C4	0.3009 (5)	0.6039 (3)	0.07775 (18)	0.0982 (8)	
H4A	0.166636	0.623780	0.036237	0.118*	
C5	0.3357 (5)	0.4880 (3)	0.10394 (18)	0.0938 (7)	
H5A	0.225083	0.428546	0.080288	0.113*	
C6	0.5327 (4)	0.45782 (18)	0.16503 (15)	0.0761 (6)	
H6A	0.555023	0.377824	0.182243	0.091*	
C7	0.9099 (4)	0.50813 (17)	0.26744 (14)	0.0686 (5)	
C8	1.0720 (4)	0.6011 (2)	0.31667 (16)	0.0836 (6)	
H8A	1.107817	0.666959	0.276182	0.100*	
H8B	1.235969	0.565970	0.337878	0.100*	
C9	0.8729 (4)	0.5233 (2)	0.48053 (15)	0.0811 (6)	
H9A	0.761790	0.541115	0.529704	0.097*	
H9B	0.789686	0.460522	0.444009	0.097*	

### 2-({2-[(2-oxo-2-phenylethyl)sulfanyl]ethyl}sulfanyl)-1-phenylethan-1-one (Dtdpe) Atomic displacement parameters (Å^2^)

**Table:**

	*U*^11^	*U*^22^	*U*^33^	*U*^12^	*U*^13^	*U*^23^
S1	0.1327 (6)	0.0691 (4)	0.0890 (5)	0.0111 (3)	−0.0008 (4)	−0.0082 (3)
O1	0.1143 (12)	0.0631 (9)	0.1051 (12)	0.0211 (8)	0.0035 (9)	0.0079 (8)
C1	0.0642 (10)	0.0494 (9)	0.0721 (11)	0.0008 (7)	0.0177 (8)	0.0010 (7)
C2	0.0802 (13)	0.0543 (10)	0.1007 (15)	0.0007 (9)	0.0112 (11)	0.0058 (10)
C3	0.1001 (17)	0.0793 (15)	0.1091 (18)	0.0245 (13)	0.0134 (14)	0.0244 (13)
C4	0.0797 (15)	0.126 (2)	0.0889 (16)	0.0098 (15)	0.0025 (12)	0.0061 (15)
C5	0.0895 (16)	0.1012 (18)	0.0905 (16)	−0.0138 (13)	0.0010 (13)	−0.0110 (13)
C6	0.0873 (14)	0.0603 (10)	0.0821 (13)	−0.0068 (9)	0.0169 (11)	−0.0046 (9)
C7	0.0727 (11)	0.0590 (10)	0.0757 (12)	0.0051 (8)	0.0191 (9)	0.0035 (8)
C8	0.0812 (13)	0.0869 (14)	0.0827 (13)	−0.0123 (11)	0.0042 (11)	0.0109 (11)
C9	0.0735 (12)	0.0924 (15)	0.0778 (13)	−0.0020 (10)	0.0091 (10)	−0.0050 (11)

### 2-({2-[(2-oxo-2-phenylethyl)sulfanyl]ethyl}sulfanyl)-1-phenylethan-1-one (Dtdpe) Geometric parameters (Å, º)

**Table:**

S1—C9	1.808 (2)	C4—H4A	0.9300
S1—C8	1.812 (2)	C5—C6	1.369 (3)
O1—C7	1.217 (2)	C5—H5A	0.9300
C1—C2	1.390 (2)	C6—H6A	0.9300
C1—C6	1.390 (3)	C7—C8	1.496 (3)
C1—C7	1.479 (3)	C8—H8A	0.9700
C2—C3	1.386 (4)	C8—H8B	0.9700
C2—H2A	0.9300	C9—C9^i^	1.499 (4)
C3—C4	1.364 (4)	C9—H9A	0.9700
C3—H3A	0.9300	C9—H9B	0.9700
C4—C5	1.354 (4)		
			
C9—S1—C8	102.24 (11)	C5—C6—H6A	119.5
C2—C1—C6	118.23 (19)	C1—C6—H6A	119.5
C2—C1—C7	122.79 (18)	O1—C7—C1	120.94 (19)
C6—C1—C7	118.98 (17)	O1—C7—C8	118.8 (2)
C3—C2—C1	119.7 (2)	C1—C7—C8	120.24 (17)
C3—C2—H2A	120.2	C7—C8—S1	109.39 (14)
C1—C2—H2A	120.2	C7—C8—H8A	109.8
C4—C3—C2	120.6 (2)	S1—C8—H8A	109.8
C4—C3—H3A	119.7	C7—C8—H8B	109.8
C2—C3—H3A	119.7	S1—C8—H8B	109.8
C5—C4—C3	120.2 (2)	H8A—C8—H8B	108.2
C5—C4—H4A	119.9	C9^i^—C9—S1	114.1 (2)
C3—C4—H4A	119.9	C9^i^—C9—H9A	108.7
C4—C5—C6	120.4 (2)	S1—C9—H9A	108.7
C4—C5—H5A	119.8	C9^i^—C9—H9B	108.7
C6—C5—H5A	119.8	S1—C9—H9B	108.7
C5—C6—C1	120.9 (2)	H9A—C9—H9B	107.6
			
C6—C1—C2—C3	0.0 (3)	C2—C1—C7—O1	−175.08 (19)
C7—C1—C2—C3	179.65 (19)	C6—C1—C7—O1	4.6 (3)
C1—C2—C3—C4	0.3 (4)	C2—C1—C7—C8	8.0 (3)
C2—C3—C4—C5	−0.3 (4)	C6—C1—C7—C8	−172.39 (18)
C3—C4—C5—C6	0.0 (4)	O1—C7—C8—S1	−95.6 (2)
C4—C5—C6—C1	0.3 (4)	C1—C7—C8—S1	81.45 (19)
C2—C1—C6—C5	−0.3 (3)	C9—S1—C8—C7	62.91 (17)
C7—C1—C6—C5	−179.98 (19)	C8—S1—C9—C9^i^	66.3 (2)

Symmetry code: (i) −*x*+2, −*y*+1, −*z*+1.

### 2-({2-[(2-oxo-2-phenylethyl)sulfanyl]ethyl}sulfanyl)-1-phenylethan-1-one (Dtdpe) Hydrogen-bond geometry (Å, º)

**Table:**

*D*—H···*A*	*D*—H	H···*A*	*D*···*A*	*D*—H···*A*
C9—H9*B*···O1	0.97	2.61	3.229 (3)	122

### 2-[(2-Oxo-2-phenylethyl)sulfanyl]-1-phenylethan-1-one (Mtdp) Crystal data

**Table:**

C_16_H_14_O_2_S	*F*(000) = 568
*M**_r_* = 270.33	*D*_x_ = 1.394 Mg m^−^^3^
Monoclinic, *I*2/*a*	Cu *K*α radiation, λ = 1.54184 Å
*a* = 13.1307 (3) Å	Cell parameters from 4447 reflections
*b* = 5.0945 (1) Å	θ = 4.0–78.5°
*c* = 19.2847 (5) Å	µ = 2.18 mm^−^^1^
β = 93.167 (2)°	*T* = 100 K
*V* = 1288.07 (5) Å^3^	Needle, colorless
*Z* = 4	0.29 × 0.10 × 0.08 mm

### 2-[(2-Oxo-2-phenylethyl)sulfanyl]-1-phenylethan-1-one (Mtdp) Data collection

**Table:**

XtaLAB Synergy, Dualflex, HyPix diffractometer	1325 independent reflections
Radiation source: micro-focus sealed X-ray tube	1253 reflections with *I* > 2σ(*I*)
Detector resolution: 10.0000 pixels mm^-1^	*R*_int_ = 0.040
ω scans	θ_max_ = 78.7°, θ_min_ = 4.6°
Absorption correction: multi-scan (CrysAlisPro; Rigaku OD, 2022)	*h* = −16→16
*T*_min_ = 0.520, *T*_max_ = 1.000	*k* = −3→6
6439 measured reflections	*l* = −23→23

### 2-[(2-Oxo-2-phenylethyl)sulfanyl]-1-phenylethan-1-one (Mtdp) Refinement

**Table:**

Refinement on *F*^2^	Primary atom site location: dual
Least-squares matrix: full	Secondary atom site location: difference Fourier map
*R*[*F*^2^ > 2σ(*F*^2^)] = 0.031	Hydrogen site location: inferred from neighbouring sites
*wR*(*F*^2^) = 0.084	H-atom parameters constrained
*S* = 1.10	*w* = 1/[σ^2^(*F*_o_^2^) + (0.0391*P*)^2^ + 1.212*P*] where *P* = (*F*_o_^2^ + 2*F*_c_^2^)/3
1325 reflections	(Δ/σ)_max_ < 0.001
87 parameters	Δρ_max_ = 0.31 e Å^−^^3^
0 restraints	Δρ_min_ = −0.26 e Å^−^^3^

### 2-[(2-Oxo-2-phenylethyl)sulfanyl]-1-phenylethan-1-one (Mtdp) Special details

**Table:**

Geometry. All esds (except the esd in the dihedral angle between two l.s. planes) are estimated using the full covariance matrix. The cell esds are taken into account individually in the estimation of esds in distances, angles and torsion angles; correlations between esds in cell parameters are only used when they are defined by crystal symmetry. An approximate (isotropic) treatment of cell esds is used for estimating esds involving l.s. planes.

### 2-[(2-Oxo-2-phenylethyl)sulfanyl]-1-phenylethan-1-one (Mtdp) Fractional atomic coordinates and isotropic or equivalent isotropic displacement parameters (Å^2^)

**Table:**

	*x*	*y*	*z*	*U*_iso_*/*U*_eq_	
S1	0.250000	0.17578 (8)	0.500000	0.01674 (15)	
O1	0.20435 (7)	0.54273 (19)	0.37979 (5)	0.0200 (2)	
C1	0.36196 (10)	0.7651 (3)	0.37252 (6)	0.0147 (3)	
C2	0.46477 (10)	0.7866 (3)	0.39467 (7)	0.0179 (3)	
H2A	0.492916	0.669275	0.428895	0.022*	
C3	0.52617 (10)	0.9778 (3)	0.36713 (7)	0.0199 (3)	
H3A	0.595891	0.991681	0.382674	0.024*	
C4	0.48543 (11)	1.1487 (3)	0.31683 (7)	0.0195 (3)	
H4A	0.527110	1.281084	0.298368	0.023*	
C5	0.38376 (11)	1.1265 (3)	0.29345 (7)	0.0191 (3)	
H5A	0.356418	1.241683	0.258425	0.023*	
C6	0.32204 (10)	0.9363 (3)	0.32117 (6)	0.0163 (3)	
H6A	0.252497	0.922447	0.305220	0.020*	
C7	0.29217 (10)	0.5669 (2)	0.40208 (6)	0.0149 (3)	
C8	0.33651 (10)	0.4024 (3)	0.46252 (7)	0.0159 (3)	
H8C	0.363100	0.522934	0.499495	0.019*	
H8A	0.395163	0.301749	0.446311	0.019*	

### 2-[(2-Oxo-2-phenylethyl)sulfanyl]-1-phenylethan-1-one (Mtdp) Atomic displacement parameters (Å^2^)

**Table:**

	*U*^11^	*U*^22^	*U*^33^	*U*^12^	*U*^13^	*U*^23^
S1	0.0217 (3)	0.0119 (2)	0.0168 (2)	0.000	0.00301 (17)	0.000
O1	0.0170 (5)	0.0213 (5)	0.0211 (5)	−0.0015 (4)	−0.0028 (4)	0.0021 (4)
C1	0.0171 (6)	0.0137 (6)	0.0132 (6)	0.0016 (5)	0.0013 (5)	−0.0018 (5)
C2	0.0187 (7)	0.0172 (6)	0.0177 (7)	0.0011 (5)	−0.0005 (5)	0.0022 (5)
C3	0.0167 (6)	0.0212 (7)	0.0219 (7)	−0.0005 (5)	0.0011 (5)	−0.0013 (5)
C4	0.0233 (7)	0.0168 (6)	0.0189 (7)	−0.0007 (5)	0.0070 (5)	−0.0001 (5)
C5	0.0236 (7)	0.0179 (6)	0.0161 (6)	0.0046 (5)	0.0029 (5)	0.0026 (5)
C6	0.0180 (6)	0.0165 (6)	0.0142 (6)	0.0032 (5)	0.0002 (5)	−0.0011 (5)
C7	0.0177 (6)	0.0137 (6)	0.0134 (6)	0.0022 (5)	0.0009 (5)	−0.0021 (5)
C8	0.0163 (6)	0.0161 (6)	0.0151 (6)	0.0007 (5)	0.0006 (5)	0.0011 (5)

### 2-[(2-Oxo-2-phenylethyl)sulfanyl]-1-phenylethan-1-one (Mtdp) Geometric parameters (Å, º)

**Table:**

S1—C8	1.7989 (13)	C3—H3A	0.9500
S1—C8^i^	1.7990 (13)	C4—C5	1.390 (2)
O1—C7	1.2145 (16)	C4—H4A	0.9500
C1—C2	1.3975 (18)	C5—C6	1.3889 (19)
C1—C6	1.3998 (18)	C5—H5A	0.9500
C1—C7	1.4974 (18)	C6—H6A	0.9500
C2—C3	1.3887 (19)	C7—C8	1.5247 (17)
C2—H2A	0.9500	C8—H8C	0.9900
C3—C4	1.389 (2)	C8—H8A	0.9900
			
C8—S1—C8^i^	100.17 (9)	C6—C5—H5A	119.9
C2—C1—C6	119.01 (12)	C4—C5—H5A	119.9
C2—C1—C7	122.52 (12)	C5—C6—C1	120.23 (12)
C6—C1—C7	118.46 (11)	C5—C6—H6A	119.9
C3—C2—C1	120.64 (12)	C1—C6—H6A	119.9
C3—C2—H2A	119.7	O1—C7—C1	121.50 (11)
C1—C2—H2A	119.7	O1—C7—C8	122.01 (12)
C4—C3—C2	119.84 (12)	C1—C7—C8	116.48 (11)
C4—C3—H3A	120.1	C7—C8—S1	115.90 (9)
C2—C3—H3A	120.1	C7—C8—H8C	108.3
C3—C4—C5	120.11 (13)	S1—C8—H8C	108.3
C3—C4—H4A	119.9	C7—C8—H8A	108.3
C5—C4—H4A	119.9	S1—C8—H8A	108.3
C6—C5—C4	120.15 (12)	H8C—C8—H8A	107.4
			
C6—C1—C2—C3	1.1 (2)	C2—C1—C7—O1	−176.36 (12)
C7—C1—C2—C3	−178.31 (12)	C6—C1—C7—O1	4.20 (19)
C1—C2—C3—C4	−0.3 (2)	C2—C1—C7—C8	5.03 (18)
C2—C3—C4—C5	−0.8 (2)	C6—C1—C7—C8	−174.42 (11)
C3—C4—C5—C6	1.1 (2)	O1—C7—C8—S1	−1.35 (17)
C4—C5—C6—C1	−0.3 (2)	C1—C7—C8—S1	177.26 (9)
C2—C1—C6—C5	−0.78 (19)	C8^i^—S1—C8—C7	−67.02 (9)
C7—C1—C6—C5	178.68 (11)		

Symmetry code: (i) −*x*+1/2, *y*, −*z*+1.

### 2-[(2-Oxo-2-phenylethyl)sulfanyl]-1-phenylethan-1-one (Mtdp) Hydrogen-bond geometry (Å, º)

**Table:**

*D*—H···*A*	*D*—H	H···*A*	*D*···*A*	*D*—H···*A*
C8—H8*C*···O1^i^	0.99	2.54	3.1981 (16)	124

Symmetry code: (i) −*x*+1/2, *y*, −*z*+1.

### 2-[(2E,12E)-3,12-Diphenyl-14-(pyridin-2-yl)-5,10-dithia-1,2,13,14-tetraazatetradeca-2,12-dien-1-yl]pyridine (Prpsb) Crystal data

**Table:**

C_30_H_32_N_6_S_2_	*F*(000) = 572
*M**_r_* = 540.73	*D*_x_ = 1.319 Mg m^−^^3^
Monoclinic, *P*2_1_/*n*	Mo *K*α radiation, λ = 0.71073 Å
*a* = 5.3442 (4) Å	Cell parameters from 8046 reflections
*b* = 24.9746 (18) Å	θ = 2.6–30.4°
*c* = 10.3552 (7) Å	µ = 0.23 mm^−^^1^
β = 99.789 (3)°	*T* = 102 K
*V* = 1361.98 (17) Å^3^	Plate, pale yellow
*Z* = 2	0.32 × 0.26 × 0.12 mm

### 2-[(2E,12E)-3,12-Diphenyl-14-(pyridin-2-yl)-5,10-dithia-1,2,13,14-tetraazatetradeca-2,12-dien-1-yl]pyridine (Prpsb) Data collection

**Table:**

Bruker APEXII CCD diffractometer	3484 reflections with *I* > 2σ(*I*)
φ and ω scans	*R*_int_ = 0.069
Absorption correction: multi-scan (SADABS; Krause *et al.*, 2015)	θ_max_ = 30.5°, θ_min_ = 2.2°
*T*_min_ = 0.354, *T*_max_ = 0.746	*h* = −6→7
19288 measured reflections	*k* = −35→35
4154 independent reflections	*l* = −14→13

### 2-[(2E,12E)-3,12-Diphenyl-14-(pyridin-2-yl)-5,10-dithia-1,2,13,14-tetraazatetradeca-2,12-dien-1-yl]pyridine (Prpsb) Refinement

**Table:**

Refinement on *F*^2^	Primary atom site location: dual
Least-squares matrix: full	Secondary atom site location: difference Fourier map
*R*[*F*^2^ > 2σ(*F*^2^)] = 0.065	Hydrogen site location: mixed
*wR*(*F*^2^) = 0.178	H atoms treated by a mixture of independent and constrained refinement
*S* = 1.03	*w* = 1/[σ^2^(*F*_o_^2^) + (0.1212*P*)^2^ + 0.2725*P*] where *P* = (*F*_o_^2^ + 2*F*_c_^2^)/3
4154 reflections	(Δ/σ)_max_ = 0.001
176 parameters	Δρ_max_ = 0.90 e Å^−^^3^
0 restraints	Δρ_min_ = −0.75 e Å^−^^3^

### 2-[(2E,12E)-3,12-Diphenyl-14-(pyridin-2-yl)-5,10-dithia-1,2,13,14-tetraazatetradeca-2,12-dien-1-yl]pyridine (Prpsb) Special details

**Table:**

Geometry. All esds (except the esd in the dihedral angle between two l.s. planes) are estimated using the full covariance matrix. The cell esds are taken into account individually in the estimation of esds in distances, angles and torsion angles; correlations between esds in cell parameters are only used when they are defined by crystal symmetry. An approximate (isotropic) treatment of cell esds is used for estimating esds involving l.s. planes.

### 2-[(2E,12E)-3,12-Diphenyl-14-(pyridin-2-yl)-5,10-dithia-1,2,13,14-tetraazatetradeca-2,12-dien-1-yl]pyridine (Prpsb) Fractional atomic coordinates and isotropic or equivalent isotropic displacement parameters (Å^2^)

**Table:**

	*x*	*y*	*z*	*U*_iso_*/*U*_eq_	
S1	0.75046 (7)	0.55513 (2)	0.69891 (4)	0.01721 (14)	
N1	0.4346 (3)	0.57053 (6)	0.23082 (14)	0.0256 (3)	
N2	0.5016 (3)	0.61895 (6)	0.42101 (13)	0.0204 (3)	
H2N	0.631 (6)	0.5943 (11)	0.450 (3)	0.047 (8)*	
C1	0.2926 (4)	0.55830 (7)	0.11510 (18)	0.0291 (4)	
H1B	0.355484	0.531946	0.062679	0.035*	
C2	0.0619 (4)	0.58139 (8)	0.06730 (17)	0.0313 (4)	
H2B	−0.032663	0.571021	−0.015002	0.038*	
N3	0.4048 (3)	0.64814 (5)	0.51246 (13)	0.0191 (3)	
C3	−0.0285 (4)	0.62047 (8)	0.14345 (17)	0.0310 (4)	
H3B	−0.187495	0.637166	0.113629	0.037*	
C4	0.1141 (4)	0.63494 (7)	0.26283 (16)	0.0249 (4)	
H4A	0.057896	0.662018	0.315661	0.030*	
C5	0.3441 (3)	0.60811 (7)	0.30249 (15)	0.0199 (3)	
C6	0.5411 (3)	0.65214 (6)	0.62770 (15)	0.0171 (3)	
C7	0.4265 (3)	0.68234 (6)	0.72588 (15)	0.0184 (3)	
C8	0.4766 (3)	0.66702 (7)	0.85775 (15)	0.0226 (3)	
H8A	0.594040	0.639013	0.884942	0.027*	
C9	0.3552 (4)	0.69260 (8)	0.94918 (17)	0.0273 (4)	
H9A	0.387163	0.681266	1.038036	0.033*	
C10	0.1887 (4)	0.73432 (7)	0.91210 (19)	0.0290 (4)	
H10A	0.107507	0.751835	0.975168	0.035*	
C11	0.1408 (4)	0.75049 (7)	0.78141 (19)	0.0287 (4)	
H11A	0.027474	0.779333	0.755438	0.034*	
C12	0.2582 (3)	0.72460 (7)	0.68881 (17)	0.0234 (3)	
H12A	0.223682	0.735714	0.599822	0.028*	
C13	0.7972 (3)	0.62633 (6)	0.66728 (15)	0.0180 (3)	
H13A	0.889858	0.643773	0.747175	0.022*	
H13B	0.898779	0.630436	0.596365	0.022*	
C14	1.0577 (3)	0.52837 (6)	0.67878 (15)	0.0193 (3)	
H14A	1.192137	0.552833	0.721957	0.023*	
H14B	1.082491	0.493347	0.723907	0.023*	
C15	1.0888 (3)	0.52097 (6)	0.53639 (15)	0.0193 (3)	
H15A	1.266421	0.510443	0.533667	0.023*	
H15B	1.057320	0.555677	0.490302	0.023*	

### 2-[(2E,12E)-3,12-Diphenyl-14-(pyridin-2-yl)-5,10-dithia-1,2,13,14-tetraazatetradeca-2,12-dien-1-yl]pyridine (Prpsb) Atomic displacement parameters (Å^2^)

**Table:**

	*U*^11^	*U*^22^	*U*^33^	*U*^12^	*U*^13^	*U*^23^
S1	0.0137 (2)	0.0168 (2)	0.0208 (2)	0.00046 (12)	0.00214 (14)	−0.00108 (11)
N1	0.0299 (8)	0.0234 (7)	0.0237 (7)	−0.0001 (6)	0.0047 (6)	−0.0021 (5)
N2	0.0196 (7)	0.0203 (7)	0.0207 (6)	0.0032 (5)	0.0018 (5)	−0.0009 (5)
C1	0.0395 (11)	0.0247 (8)	0.0233 (8)	−0.0022 (7)	0.0059 (7)	−0.0026 (6)
C2	0.0365 (10)	0.0337 (10)	0.0210 (8)	−0.0083 (8)	−0.0029 (7)	0.0002 (6)
N3	0.0196 (7)	0.0169 (6)	0.0200 (6)	−0.0001 (5)	0.0016 (5)	0.0010 (4)
C3	0.0264 (9)	0.0407 (11)	0.0235 (8)	0.0030 (8)	−0.0026 (7)	0.0050 (7)
C4	0.0248 (8)	0.0270 (8)	0.0221 (8)	0.0035 (7)	0.0018 (6)	0.0012 (6)
C5	0.0213 (8)	0.0183 (7)	0.0197 (7)	−0.0030 (6)	0.0027 (5)	0.0019 (5)
C6	0.0171 (7)	0.0136 (6)	0.0200 (7)	−0.0010 (5)	0.0018 (5)	0.0001 (5)
C7	0.0167 (7)	0.0158 (6)	0.0221 (7)	−0.0030 (5)	0.0018 (5)	−0.0017 (5)
C8	0.0224 (8)	0.0227 (7)	0.0211 (7)	0.0030 (6)	−0.0002 (6)	−0.0020 (5)
C9	0.0294 (9)	0.0291 (9)	0.0233 (8)	−0.0003 (7)	0.0041 (7)	−0.0041 (6)
C10	0.0303 (9)	0.0242 (8)	0.0343 (9)	0.0000 (7)	0.0106 (7)	−0.0090 (7)
C11	0.0315 (9)	0.0183 (7)	0.0372 (10)	0.0052 (7)	0.0085 (7)	−0.0023 (6)
C12	0.0256 (8)	0.0155 (7)	0.0290 (8)	0.0015 (6)	0.0047 (7)	0.0012 (5)
C13	0.0144 (7)	0.0164 (7)	0.0228 (7)	−0.0013 (5)	0.0021 (5)	−0.0021 (5)
C14	0.0136 (7)	0.0213 (7)	0.0218 (7)	0.0022 (6)	−0.0002 (5)	−0.0031 (5)
C15	0.0136 (7)	0.0209 (7)	0.0233 (7)	−0.0024 (6)	0.0031 (5)	−0.0041 (5)

### 2-[(2E,12E)-3,12-Diphenyl-14-(pyridin-2-yl)-5,10-dithia-1,2,13,14-tetraazatetradeca-2,12-dien-1-yl]pyridine (Prpsb) Geometric parameters (Å, º)

**Table:**

S1—C14	1.8172 (16)	C7—C8	1.400 (2)
S1—C13	1.8329 (16)	C8—C9	1.391 (2)
N1—C5	1.337 (2)	C8—H8A	0.9500
N1—C1	1.340 (2)	C9—C10	1.382 (3)
N2—N3	1.3647 (19)	C9—H9A	0.9500
N2—C5	1.392 (2)	C10—C11	1.394 (3)
N2—H2N	0.94 (3)	C10—H10A	0.9500
C1—C2	1.375 (3)	C11—C12	1.391 (2)
C1—H1B	0.9500	C11—H11A	0.9500
C2—C3	1.392 (3)	C12—H12A	0.9500
C2—H2B	0.9500	C13—H13A	0.9900
N3—C6	1.292 (2)	C13—H13B	0.9900
C3—C4	1.385 (2)	C14—C15	1.523 (2)
C3—H3B	0.9500	C14—H14A	0.9900
C4—C5	1.399 (2)	C14—H14B	0.9900
C4—H4A	0.9500	C15—C15^i^	1.524 (3)
C6—C7	1.479 (2)	C15—H15A	0.9900
C6—C13	1.505 (2)	C15—H15B	0.9900
C7—C12	1.397 (2)		
			
C14—S1—C13	100.55 (7)	C10—C9—C8	120.68 (17)
C5—N1—C1	116.93 (17)	C10—C9—H9A	119.7
N3—N2—C5	118.16 (14)	C8—C9—H9A	119.7
N3—N2—H2N	118.3 (17)	C9—C10—C11	119.45 (16)
C5—N2—H2N	117.3 (17)	C9—C10—H10A	120.3
N1—C1—C2	124.27 (18)	C11—C10—H10A	120.3
N1—C1—H1B	117.9	C12—C11—C10	120.30 (17)
C2—C1—H1B	117.9	C12—C11—H11A	119.8
C1—C2—C3	117.81 (17)	C10—C11—H11A	119.8
C1—C2—H2B	121.1	C11—C12—C7	120.42 (16)
C3—C2—H2B	121.1	C11—C12—H12A	119.8
C6—N3—N2	117.49 (14)	C7—C12—H12A	119.8
C4—C3—C2	119.83 (18)	C6—C13—S1	108.60 (10)
C4—C3—H3B	120.1	C6—C13—H13A	110.0
C2—C3—H3B	120.1	S1—C13—H13A	110.0
C3—C4—C5	117.34 (17)	C6—C13—H13B	110.0
C3—C4—H4A	121.3	S1—C13—H13B	110.0
C5—C4—H4A	121.3	H13A—C13—H13B	108.4
N1—C5—N2	113.84 (15)	C15—C14—S1	113.90 (11)
N1—C5—C4	123.79 (15)	C15—C14—H14A	108.8
N2—C5—C4	122.36 (15)	S1—C14—H14A	108.8
N3—C6—C7	116.10 (14)	C15—C14—H14B	108.8
N3—C6—C13	124.20 (14)	S1—C14—H14B	108.8
C7—C6—C13	119.64 (13)	H14A—C14—H14B	107.7
C12—C7—C8	118.80 (15)	C14—C15—C15^i^	113.63 (17)
C12—C7—C6	121.24 (14)	C14—C15—H15A	108.8
C8—C7—C6	119.89 (14)	C15^i^—C15—H15A	108.8
C9—C8—C7	120.32 (16)	C14—C15—H15B	108.8
C9—C8—H8A	119.8	C15^i^—C15—H15B	108.8
C7—C8—H8A	119.8	H15A—C15—H15B	107.7
			
C5—N1—C1—C2	−0.7 (3)	N3—C6—C7—C8	145.96 (16)
N1—C1—C2—C3	0.8 (3)	C13—C6—C7—C8	−31.4 (2)
C5—N2—N3—C6	171.38 (14)	C12—C7—C8—C9	1.7 (3)
C1—C2—C3—C4	0.3 (3)	C6—C7—C8—C9	−175.23 (16)
C2—C3—C4—C5	−1.3 (3)	C7—C8—C9—C10	−1.7 (3)
C1—N1—C5—N2	−179.72 (15)	C8—C9—C10—C11	0.5 (3)
C1—N1—C5—C4	−0.5 (3)	C9—C10—C11—C12	0.5 (3)
N3—N2—C5—N1	−166.76 (14)	C10—C11—C12—C7	−0.4 (3)
N3—N2—C5—C4	14.0 (2)	C8—C7—C12—C11	−0.7 (3)
C3—C4—C5—N1	1.5 (3)	C6—C7—C12—C11	176.21 (16)
C3—C4—C5—N2	−179.37 (16)	N3—C6—C13—S1	−78.09 (17)
N2—N3—C6—C7	−177.79 (13)	C7—C6—C13—S1	99.02 (14)
N2—N3—C6—C13	−0.6 (2)	C14—S1—C13—C6	158.22 (11)
N3—C6—C7—C12	−30.9 (2)	C13—S1—C14—C15	−79.10 (12)
C13—C6—C7—C12	151.77 (15)	S1—C14—C15—C15^i^	−65.2 (2)

Symmetry code: (i) −*x*+2, −*y*+1, −*z*+1.

### 2-[(2E,12E)-3,12-Diphenyl-14-(pyridin-2-yl)-5,10-dithia-1,2,13,14-tetraazatetradeca-2,12-dien-1-yl]pyridine (Prpsb) Hydrogen-bond geometry (Å, º)

**Table:**

*D*—H···*A*	*D*—H	H···*A*	*D*···*A*	*D*—H···*A*
N2—H2*N*···S1	0.94 (3)	2.73 (3)	3.3599 (15)	126 (2)
C2—H2*B*···S1^ii^	0.95	3.02	3.9489 (18)	167

Symmetry code: (ii) *x*−1, *y*, *z*−1.

### (3E,8Z)-3,9-Dimethyl-1,11-dithia-4,5,7,8-tetraazacyclotetradeca-3,8-diene-6-thione (Ctrsp) Crystal data

**Table:**

C_10_H_18_N_4_S_3_	*F*(000) = 616
*M**_r_* = 290.46	*D*_x_ = 1.374 Mg m^−^^3^
Monoclinic, *P*2_1_/*c*	Cu *K*α radiation, λ = 1.54184 Å
*a* = 9.1219 (3) Å	Cell parameters from 50347 reflections
*b* = 8.6565 (2) Å	θ = 4.8–78.6°
*c* = 17.7935 (7) Å	µ = 4.70 mm^−^^1^
β = 92.195 (3)°	*T* = 293 K
*V* = 1404.01 (8) Å^3^	Block, pale yellow
*Z* = 4	0.40 × 0.34 × 0.20 mm

### (3E,8Z)-3,9-Dimethyl-1,11-dithia-4,5,7,8-tetraazacyclotetradeca-3,8-diene-6-thione (Ctrsp) Data collection

**Table:**

XtaLAB Synergy, Dualflex, HyPix diffractometer	2983 independent reflections
Radiation source: micro-focus sealed X-ray tube	2806 reflections with *I* > 2σ(*I*)
Detector resolution: 10.0000 pixels mm^-1^	*R*_int_ = 0.053
ω scans	θ_max_ = 78.9°, θ_min_ = 4.9°
Absorption correction: multi-scan (CrysAlisPro; Rigaku OD, 2022)	*h* = −11→11
*T*_min_ = 0.683, *T*_max_ = 1.000	*k* = −9→11
75253 measured reflections	*l* = −22→22

### (3E,8Z)-3,9-Dimethyl-1,11-dithia-4,5,7,8-tetraazacyclotetradeca-3,8-diene-6-thione (Ctrsp) Refinement

**Table:**

Refinement on *F*^2^	Primary atom site location: dual
Least-squares matrix: full	Secondary atom site location: difference Fourier map
*R*[*F*^2^ > 2σ(*F*^2^)] = 0.031	Hydrogen site location: mixed
*wR*(*F*^2^) = 0.087	H atoms treated by a mixture of independent and constrained refinement
*S* = 1.08	*w* = 1/[σ^2^(*F*_o_^2^) + (0.0445*P*)^2^ + 0.3926*P*] where *P* = (*F*_o_^2^ + 2*F*_c_^2^)/3
2983 reflections	(Δ/σ)_max_ = 0.001
164 parameters	Δρ_max_ = 0.22 e Å^−^^3^
0 restraints	Δρ_min_ = −0.28 e Å^−^^3^

### (3E,8Z)-3,9-Dimethyl-1,11-dithia-4,5,7,8-tetraazacyclotetradeca-3,8-diene-6-thione (Ctrsp) Special details

**Table:**

Geometry. All esds (except the esd in the dihedral angle between two l.s. planes) are estimated using the full covariance matrix. The cell esds are taken into account individually in the estimation of esds in distances, angles and torsion angles; correlations between esds in cell parameters are only used when they are defined by crystal symmetry. An approximate (isotropic) treatment of cell esds is used for estimating esds involving l.s. planes.

### (3E,8Z)-3,9-Dimethyl-1,11-dithia-4,5,7,8-tetraazacyclotetradeca-3,8-diene-6-thione (Ctrsp) Fractional atomic coordinates and isotropic or equivalent isotropic displacement parameters (Å^2^)

**Table:**

	*x*	*y*	*z*	*U*_iso_*/*U*_eq_	
S1	0.69762 (4)	0.13162 (5)	0.53637 (3)	0.05242 (13)	
S2	0.03160 (5)	0.40185 (5)	0.70320 (3)	0.05621 (13)	
S3	0.53376 (4)	0.64536 (5)	0.71956 (2)	0.04978 (12)	
N1	0.42456 (13)	0.18596 (15)	0.57232 (7)	0.0421 (3)	
H1N	0.406 (2)	0.098 (2)	0.5560 (12)	0.061 (6)*	
N2	0.31058 (13)	0.28979 (14)	0.58268 (7)	0.0408 (3)	
N3	0.70338 (13)	0.47426 (15)	0.57665 (7)	0.0434 (3)	
N4	0.57215 (14)	0.39538 (15)	0.58005 (7)	0.0423 (3)	
H4N	0.497 (2)	0.433 (2)	0.5967 (11)	0.053 (5)*	
C1	0.56086 (15)	0.24457 (16)	0.56344 (7)	0.0378 (3)	
C2	0.18561 (16)	0.23626 (18)	0.60005 (8)	0.0431 (3)	
C3	0.1472 (2)	0.0727 (2)	0.61565 (12)	0.0641 (5)	
H3A	0.235385	0.014447	0.625703	0.096*	
H3B	0.094818	0.029794	0.572731	0.096*	
H3C	0.086755	0.068214	0.658572	0.096*	
C4	0.06794 (17)	0.3563 (2)	0.60637 (9)	0.0486 (4)	
H4A	−0.021574	0.319193	0.581314	0.058*	
H4B	0.097687	0.449631	0.580904	0.058*	
C5	0.19995 (19)	0.4978 (2)	0.73331 (9)	0.0519 (4)	
H5A	0.197519	0.516916	0.786970	0.062*	
H5B	0.281462	0.428758	0.724999	0.062*	
C6	0.22813 (18)	0.64884 (19)	0.69410 (9)	0.0497 (4)	
H6A	0.140912	0.712743	0.695429	0.060*	
H6B	0.247669	0.628655	0.641806	0.060*	
C7	0.3568 (2)	0.7359 (2)	0.73024 (10)	0.0557 (4)	
H7A	0.340343	0.747291	0.783489	0.067*	
H7B	0.359541	0.838632	0.708604	0.067*	
C8	0.56680 (17)	0.70076 (18)	0.62292 (9)	0.0458 (3)	
H8A	0.481977	0.675051	0.590806	0.055*	
H8B	0.582617	0.811384	0.620125	0.055*	
C9	0.69942 (17)	0.61676 (17)	0.59655 (8)	0.0422 (3)	
C10	0.83900 (18)	0.7069 (2)	0.59552 (10)	0.0535 (4)	
H10A	0.913013	0.646324	0.572404	0.080*	
H10B	0.870766	0.732008	0.646114	0.080*	
H10C	0.822889	0.800419	0.567373	0.080*	

### (3E,8Z)-3,9-Dimethyl-1,11-dithia-4,5,7,8-tetraazacyclotetradeca-3,8-diene-6-thione (Ctrsp) Atomic displacement parameters (Å^2^)

**Table:**

	*U*^11^	*U*^22^	*U*^33^	*U*^12^	*U*^13^	*U*^23^
S1	0.0416 (2)	0.0428 (2)	0.0728 (3)	0.00578 (15)	0.00102 (17)	−0.01643 (18)
S2	0.0541 (2)	0.0559 (3)	0.0599 (2)	−0.00071 (18)	0.01997 (18)	−0.00550 (18)
S3	0.0529 (2)	0.0508 (2)	0.0451 (2)	0.00232 (16)	−0.00629 (16)	−0.00093 (15)
N1	0.0419 (6)	0.0334 (6)	0.0512 (7)	0.0007 (5)	0.0045 (5)	−0.0070 (5)
N2	0.0404 (6)	0.0378 (6)	0.0444 (6)	0.0041 (5)	0.0031 (5)	−0.0043 (5)
N3	0.0411 (6)	0.0389 (7)	0.0499 (7)	−0.0008 (5)	−0.0008 (5)	−0.0034 (5)
N4	0.0393 (6)	0.0355 (6)	0.0519 (7)	0.0006 (5)	0.0022 (5)	−0.0060 (5)
C1	0.0424 (7)	0.0348 (7)	0.0360 (6)	0.0024 (5)	−0.0017 (5)	−0.0027 (5)
C2	0.0436 (7)	0.0431 (8)	0.0429 (7)	−0.0008 (6)	0.0048 (6)	−0.0054 (6)
C3	0.0620 (11)	0.0471 (10)	0.0850 (13)	−0.0051 (8)	0.0263 (9)	−0.0033 (9)
C4	0.0416 (8)	0.0504 (9)	0.0538 (9)	0.0029 (6)	0.0037 (6)	−0.0048 (7)
C5	0.0585 (9)	0.0528 (10)	0.0447 (8)	0.0045 (7)	0.0051 (7)	−0.0026 (7)
C6	0.0497 (8)	0.0459 (9)	0.0537 (9)	0.0083 (7)	0.0022 (7)	−0.0051 (7)
C7	0.0647 (10)	0.0457 (9)	0.0567 (9)	0.0047 (8)	0.0035 (7)	−0.0161 (7)
C8	0.0525 (8)	0.0349 (8)	0.0496 (8)	0.0043 (6)	−0.0025 (6)	0.0012 (6)
C9	0.0466 (8)	0.0356 (7)	0.0441 (7)	0.0003 (6)	−0.0044 (6)	0.0008 (6)
C10	0.0516 (9)	0.0419 (9)	0.0666 (10)	−0.0049 (7)	−0.0036 (7)	−0.0015 (7)

### (3E,8Z)-3,9-Dimethyl-1,11-dithia-4,5,7,8-tetraazacyclotetradeca-3,8-diene-6-thione (Ctrsp) Geometric parameters (Å, º)

**Table:**

S1—C1	1.6704 (14)	C4—H4A	0.9700
S2—C5	1.8091 (18)	C4—H4B	0.9700
S2—C4	1.8104 (17)	C5—C6	1.508 (2)
S3—C7	1.8109 (18)	C5—H5A	0.9700
S3—C8	1.8214 (16)	C5—H5B	0.9700
N1—C1	1.3577 (18)	C6—C7	1.517 (2)
N1—N2	1.3919 (17)	C6—H6A	0.9700
N1—H1N	0.83 (2)	C6—H6B	0.9700
N2—C2	1.2794 (19)	C7—H7A	0.9700
N3—C9	1.2843 (19)	C7—H7B	0.9700
N3—N4	1.3814 (18)	C8—C9	1.502 (2)
N4—C1	1.3416 (19)	C8—H8A	0.9700
N4—H4N	0.83 (2)	C8—H8B	0.9700
C2—C3	1.487 (2)	C9—C10	1.494 (2)
C2—C4	1.501 (2)	C10—H10A	0.9600
C3—H3A	0.9600	C10—H10B	0.9600
C3—H3B	0.9600	C10—H10C	0.9600
C3—H3C	0.9600		
			
C5—S2—C4	101.40 (7)	C6—C5—H5B	108.6
C7—S3—C8	99.53 (8)	S2—C5—H5B	108.6
C1—N1—N2	117.78 (12)	H5A—C5—H5B	107.6
C1—N1—H1N	118.5 (14)	C5—C6—C7	112.15 (14)
N2—N1—H1N	119.7 (14)	C5—C6—H6A	109.2
C2—N2—N1	118.37 (13)	C7—C6—H6A	109.2
C9—N3—N4	115.41 (13)	C5—C6—H6B	109.2
C1—N4—N3	122.05 (13)	C7—C6—H6B	109.2
C1—N4—H4N	114.1 (14)	H6A—C6—H6B	107.9
N3—N4—H4N	123.6 (14)	C6—C7—S3	114.77 (11)
N4—C1—N1	113.64 (13)	C6—C7—H7A	108.6
N4—C1—S1	125.54 (11)	S3—C7—H7A	108.6
N1—C1—S1	120.81 (11)	C6—C7—H7B	108.6
N2—C2—C3	127.45 (14)	S3—C7—H7B	108.6
N2—C2—C4	114.43 (14)	H7A—C7—H7B	107.6
C3—C2—C4	118.10 (14)	C9—C8—S3	109.38 (10)
C2—C3—H3A	109.5	C9—C8—H8A	109.8
C2—C3—H3B	109.5	S3—C8—H8A	109.8
H3A—C3—H3B	109.5	C9—C8—H8B	109.8
C2—C3—H3C	109.5	S3—C8—H8B	109.8
H3A—C3—H3C	109.5	H8A—C8—H8B	108.2
H3B—C3—H3C	109.5	N3—C9—C10	117.74 (14)
C2—C4—S2	112.30 (11)	N3—C9—C8	125.64 (14)
C2—C4—H4A	109.1	C10—C9—C8	116.61 (13)
S2—C4—H4A	109.1	C9—C10—H10A	109.5
C2—C4—H4B	109.1	C9—C10—H10B	109.5
S2—C4—H4B	109.1	H10A—C10—H10B	109.5
H4A—C4—H4B	107.9	C9—C10—H10C	109.5
C6—C5—S2	114.76 (12)	H10A—C10—H10C	109.5
C6—C5—H5A	108.6	H10B—C10—H10C	109.5
S2—C5—H5A	108.6		
			
C1—N1—N2—C2	−171.77 (13)	C5—S2—C4—C2	68.26 (13)
C9—N3—N4—C1	−177.66 (14)	C4—S2—C5—C6	65.09 (13)
N3—N4—C1—N1	177.97 (12)	S2—C5—C6—C7	170.72 (11)
N3—N4—C1—S1	−0.4 (2)	C5—C6—C7—S3	67.92 (17)
N2—N1—C1—N4	11.94 (18)	C8—S3—C7—C6	78.04 (15)
N2—N1—C1—S1	−169.57 (10)	C7—S3—C8—C9	−171.14 (11)
N1—N2—C2—C3	4.1 (2)	N4—N3—C9—C10	178.75 (13)
N1—N2—C2—C4	−177.19 (12)	N4—N3—C9—C8	0.4 (2)
N2—C2—C4—S2	−103.13 (14)	S3—C8—C9—N3	76.54 (17)
C3—C2—C4—S2	75.67 (17)	S3—C8—C9—C10	−101.80 (14)

### (3E,8Z)-3,9-Dimethyl-1,11-dithia-4,5,7,8-tetraazacyclotetradeca-3,8-diene-6-thione (Ctrsp) Hydrogen-bond geometry (Å, º)

**Table:**

*D*—H···*A*	*D*—H	H···*A*	*D*···*A*	*D*—H···*A*
N1—H1*N*···S1^i^	0.83 (2)	2.73 (2)	3.5178 (14)	160.4 (19)
N4—H4*N*···S3	0.83 (2)	2.864 (19)	3.3218 (13)	116.9 (15)
N4—H4*N*···N2	0.83 (2)	2.11 (2)	2.5572 (18)	113.5 (16)
C6—H6*A*···S2^ii^	0.97	2.94	3.7524 (17)	142

Symmetry codes: (i) −*x*+1, −*y*, −*z*+1; (ii) −*x*, *y*+1/2, −*z*+3/2.

### 2-((2E)-2-{(2Z)-2-Phenyl-2-[2-(pyridin-2-yl)hydrazin-1-ylidene]ethylidene}hydrazin-1-yl)pyridine (Dhpk) Crystal data

**Table:**

C_18_H_16_N_6_	*F*(000) = 5312
*M**_r_* = 316.37	*D*_x_ = 1.364 Mg m^−^^3^
Monoclinic, *I*2/*a*	Cu *K*α radiation, λ = 1.54184 Å
*a* = 34.4957 (2) Å	Cell parameters from 70062 reflections
*b* = 10.4262 (1) Å	θ = 2.9–76.2°
*c* = 37.9992 (3) Å	µ = 0.69 mm^−^^1^
β = 115.604 (1)°	*T* = 100 K
*V* = 12324.7 (2) Å^3^	Thick needle, colorless
*Z* = 32	0.44 × 0.23 × 0.18 mm

### 2-((2E)-2-{(2Z)-2-Phenyl-2-[2-(pyridin-2-yl)hydrazin-1-ylidene]ethylidene}hydrazin-1-yl)pyridine (Dhpk) Data collection

**Table:**

XtaLAB Synergy, Dualflex, HyPix diffractometer	12730 independent reflections
Radiation source: micro-focus sealed X-ray tube	8570 reflections with *I* > 2σ(*I*)
Detector resolution: 10.0000 pixels mm^-1^	*R*_int_ = 0.068
ω scans	θ_max_ = 76.3°, θ_min_ = 2.8°
Absorption correction: multi-scan (CrysAlisPro; Rigaku OD, 2022)	*h* = −43→43
*T*_min_ = 0.582, *T*_max_ = 1.000	*k* = −13→13
219947 measured reflections	*l* = −47→45

### 2-((2E)-2-{(2Z)-2-Phenyl-2-[2-(pyridin-2-yl)hydrazin-1-ylidene]ethylidene}hydrazin-1-yl)pyridine (Dhpk) Refinement

**Table:**

Refinement on *F*^2^	Primary atom site location: dual
Least-squares matrix: full	Secondary atom site location: difference Fourier map
*R*[*F*^2^ > 2σ(*F*^2^)] = 0.041	Hydrogen site location: mixed
*wR*(*F*^2^) = 0.138	H atoms treated by a mixture of independent and constrained refinement
*S* = 1.04	*w* = 1/[σ^2^(*F*_o_^2^) + (0.0792*P*)^2^ + 2.6809*P*] where *P* = (*F*_o_^2^ + 2*F*_c_^2^)/3
12730 reflections	(Δ/σ)_max_ = 0.001
897 parameters	Δρ_max_ = 0.20 e Å^−^^3^
0 restraints	Δρ_min_ = −0.31 e Å^−^^3^

### 2-((2E)-2-{(2Z)-2-Phenyl-2-[2-(pyridin-2-yl)hydrazin-1-ylidene]ethylidene}hydrazin-1-yl)pyridine (Dhpk) Special details

**Table:**

Geometry. All esds (except the esd in the dihedral angle between two l.s. planes) are estimated using the full covariance matrix. The cell esds are taken into account individually in the estimation of esds in distances, angles and torsion angles; correlations between esds in cell parameters are only used when they are defined by crystal symmetry. An approximate (isotropic) treatment of cell esds is used for estimating esds involving l.s. planes.

### 2-((2E)-2-{(2Z)-2-Phenyl-2-[2-(pyridin-2-yl)hydrazin-1-ylidene]ethylidene}hydrazin-1-yl)pyridine (Dhpk) Fractional atomic coordinates and isotropic or equivalent isotropic displacement parameters (Å^2^)

**Table:**

	*x*	*y*	*z*	*U*_iso_*/*U*_eq_	
N1A	0.38832 (3)	1.55230 (8)	0.28953 (3)	0.0202 (2)	
N2A	0.35056 (3)	1.43696 (8)	0.23398 (3)	0.0194 (2)	
H2NA	0.3415 (5)	1.3897 (14)	0.2487 (5)	0.038 (4)*	
N3A	0.33946 (3)	1.41244 (8)	0.19619 (3)	0.0190 (2)	
N4A	0.30205 (3)	1.23606 (8)	0.23274 (3)	0.0186 (2)	
N5A	0.28486 (3)	1.14681 (8)	0.24767 (3)	0.0189 (2)	
H5NA	0.2673 (4)	1.0824 (13)	0.2329 (4)	0.027 (3)*	
N6A	0.27820 (3)	1.05544 (8)	0.29969 (3)	0.0193 (2)	
C1A	0.41346 (3)	1.65258 (10)	0.30759 (3)	0.0216 (2)	
H1AA	0.420173	1.666278	0.334322	0.026*	
C2A	0.43024 (3)	1.73737 (10)	0.28968 (4)	0.0225 (2)	
H2AA	0.447405	1.808095	0.303557	0.027*	
C3A	0.42120 (4)	1.71584 (10)	0.25076 (4)	0.0228 (2)	
H3AA	0.432764	1.770905	0.237772	0.027*	
C4A	0.39530 (3)	1.61387 (10)	0.23112 (3)	0.0202 (2)	
H4AA	0.388759	1.596835	0.204587	0.024*	
C5A	0.37900 (3)	1.53633 (9)	0.25186 (3)	0.0182 (2)	
C6A	0.31404 (3)	1.31595 (10)	0.17903 (3)	0.0179 (2)	
C7A	0.30655 (3)	1.29757 (9)	0.13773 (3)	0.0187 (2)	
C8A	0.33945 (3)	1.32872 (10)	0.12689 (3)	0.0241 (2)	
H8AA	0.366284	1.358482	0.146118	0.029*	
C9A	0.33336 (4)	1.31672 (10)	0.08863 (3)	0.0267 (2)	
H9AA	0.356005	1.337738	0.081768	0.032*	
C10A	0.29409 (4)	1.27388 (10)	0.06010 (3)	0.0236 (2)	
H10A	0.289757	1.266495	0.033730	0.028*	
C11A	0.26143 (3)	1.24215 (9)	0.07046 (3)	0.0227 (2)	
H11A	0.234595	1.212929	0.051104	0.027*	
C12A	0.26767 (3)	1.25277 (9)	0.10901 (3)	0.0211 (2)	
H12A	0.245202	1.229254	0.115859	0.025*	
C13A	0.29491 (3)	1.22636 (10)	0.19650 (3)	0.0188 (2)	
H13A	0.276856	1.159623	0.180977	0.023*	
C14A	0.29536 (3)	1.15010 (9)	0.28698 (3)	0.0176 (2)	
C15A	0.32216 (4)	1.24571 (10)	0.31184 (4)	0.0215 (2)	
H15A	0.333369	1.312585	0.301948	0.026*	
C16A	0.33160 (3)	1.23925 (10)	0.35092 (4)	0.0222 (2)	
H16A	0.349670	1.302152	0.368409	0.027*	
C17A	0.31470 (3)	1.14062 (10)	0.36488 (3)	0.0216 (2)	
H17A	0.321136	1.133979	0.391780	0.026*	
C18A	0.28820 (3)	1.05297 (10)	0.33793 (3)	0.0213 (2)	
H18A	0.276223	0.986250	0.347149	0.026*	
N1C	0.63757 (3)	−0.29748 (8)	0.54010 (3)	0.0212 (2)	
N2C	0.59888 (3)	−0.18330 (8)	0.48453 (3)	0.0198 (2)	
H2NC	0.5905 (5)	−0.1328 (14)	0.4994 (5)	0.040 (4)*	
N3C	0.58684 (3)	−0.15853 (8)	0.44649 (3)	0.0191 (2)	
N4C	0.55372 (3)	0.02391 (8)	0.48561 (3)	0.0192 (2)	
N5C	0.53575 (3)	0.11109 (8)	0.50049 (3)	0.0196 (2)	
H5NC	0.5169 (4)	0.1721 (13)	0.4856 (4)	0.029 (3)*	
N6C	0.52555 (3)	0.19348 (8)	0.55182 (3)	0.0192 (2)	
C1C	0.66273 (3)	−0.39810 (10)	0.55801 (3)	0.0221 (2)	
H1CA	0.670306	−0.410300	0.584964	0.026*	
C2C	0.67835 (3)	−0.48522 (10)	0.53975 (4)	0.0228 (2)	
H2CA	0.695879	−0.555265	0.553680	0.027*	
C3C	0.66754 (3)	−0.46673 (10)	0.50039 (4)	0.0221 (2)	
H3CA	0.678067	−0.523737	0.487021	0.027*	
C4C	0.64143 (3)	−0.36514 (10)	0.48077 (3)	0.0201 (2)	
H4CA	0.633603	−0.350745	0.453860	0.024*	
C5C	0.62684 (3)	−0.28381 (9)	0.50203 (3)	0.0184 (2)	
C6C	0.56252 (3)	−0.05870 (10)	0.43043 (3)	0.0189 (2)	
C7C	0.55180 (3)	−0.04087 (10)	0.38843 (3)	0.0199 (2)	
C8C	0.54561 (3)	−0.14741 (10)	0.36438 (3)	0.0234 (2)	
H8CA	0.547299	−0.231206	0.374767	0.028*	
C9C	0.53700 (3)	−0.13210 (11)	0.32546 (3)	0.0272 (2)	
H9CA	0.532870	−0.205495	0.309415	0.033*	
C10C	0.53438 (4)	−0.01059 (12)	0.30973 (4)	0.0286 (3)	
H10C	0.528436	−0.000356	0.283038	0.034*	
C11C	0.54057 (4)	0.09623 (11)	0.33357 (3)	0.0302 (3)	
H11C	0.538974	0.179798	0.323070	0.036*	
C12C	0.54905 (3)	0.08167 (10)	0.37255 (3)	0.0254 (2)	
H12C	0.552995	0.155234	0.388490	0.030*	
C13C	0.54529 (3)	0.03240 (10)	0.44911 (3)	0.0192 (2)	
H13C	0.527342	0.099997	0.434004	0.023*	
C14C	0.54512 (3)	0.10451 (9)	0.53950 (3)	0.0184 (2)	
C15C	0.57312 (4)	0.01118 (10)	0.56450 (4)	0.0214 (2)	
H15C	0.586507	−0.050581	0.554994	0.026*	
C16C	0.58046 (4)	0.01232 (10)	0.60312 (4)	0.0226 (2)	
H16C	0.599318	−0.048957	0.620716	0.027*	
C17C	0.56027 (3)	0.10325 (10)	0.61644 (3)	0.0220 (2)	
H17C	0.564733	0.104934	0.642915	0.026*	
C18C	0.53352 (4)	0.19079 (10)	0.58964 (3)	0.0221 (2)	
H18C	0.519852	0.253327	0.598602	0.026*	
N1B	0.35843 (3)	0.79933 (8)	0.45970 (3)	0.0233 (2)	
N2B	0.40002 (3)	0.69341 (8)	0.51614 (3)	0.0192 (2)	
H2NB	0.4088 (5)	0.6457 (14)	0.5020 (5)	0.041 (4)*	
N3B	0.41090 (3)	0.66739 (8)	0.55379 (3)	0.0187 (2)	
N4B	0.44785 (3)	0.49019 (8)	0.51685 (3)	0.0184 (2)	
N5B	0.46443 (3)	0.39976 (8)	0.50175 (3)	0.0186 (2)	
H5NB	0.4815 (4)	0.3347 (14)	0.5164 (4)	0.032 (4)*	
N6B	0.47035 (3)	0.30701 (8)	0.44954 (3)	0.0191 (2)	
C1B	0.32985 (4)	0.89161 (10)	0.44079 (4)	0.0267 (3)	
H1BA	0.321466	0.901391	0.413610	0.032*	
C2B	0.31182 (4)	0.97331 (10)	0.45825 (4)	0.0267 (3)	
H2BA	0.291976	1.037896	0.443600	0.032*	
C3B	0.32361 (4)	0.95809 (10)	0.49786 (4)	0.0256 (3)	
H3BA	0.311681	1.012185	0.510772	0.031*	
C4B	0.35278 (3)	0.86384 (10)	0.51841 (3)	0.0216 (2)	
H4BA	0.361156	0.851001	0.545473	0.026*	
C5B	0.36959 (3)	0.78782 (9)	0.49783 (3)	0.0190 (2)	
C6B	0.43606 (3)	0.57003 (10)	0.57072 (3)	0.0182 (2)	
C7B	0.44320 (3)	0.54996 (9)	0.61183 (3)	0.0184 (2)	
C8B	0.40987 (3)	0.57859 (10)	0.62239 (3)	0.0238 (2)	
H8BA	0.383013	0.607948	0.603095	0.029*	
C9B	0.41565 (3)	0.56458 (10)	0.66051 (3)	0.0265 (2)	
H9BA	0.392706	0.583642	0.667166	0.032*	
C10B	0.45486 (4)	0.52273 (10)	0.68918 (3)	0.0232 (2)	
H10B	0.458923	0.514297	0.715445	0.028*	
C11B	0.48797 (3)	0.49342 (9)	0.67915 (3)	0.0229 (2)	
H11B	0.514856	0.464998	0.698633	0.027*	
C12B	0.48205 (3)	0.50541 (9)	0.64069 (3)	0.0208 (2)	
H12B	0.504733	0.482998	0.634018	0.025*	
C13B	0.45480 (3)	0.48006 (10)	0.55295 (3)	0.0186 (2)	
H13B	0.472517	0.412484	0.568314	0.022*	
C14B	0.45402 (3)	0.40337 (9)	0.46254 (3)	0.0176 (2)	
C15B	0.42796 (3)	0.50061 (10)	0.43773 (3)	0.0204 (2)	
H15B	0.417463	0.568912	0.447749	0.025*	
C16B	0.41818 (3)	0.49377 (10)	0.39860 (4)	0.0221 (2)	
H16B	0.400520	0.557543	0.381190	0.027*	
C17B	0.43421 (3)	0.39316 (10)	0.38450 (3)	0.0214 (2)	
H17B	0.427542	0.386136	0.357559	0.026*	
C18B	0.46014 (3)	0.30422 (10)	0.41127 (3)	0.0213 (2)	
H18B	0.471540	0.236359	0.401913	0.026*	
N1D	0.10914 (3)	0.45502 (8)	0.20909 (3)	0.0233 (2)	
N2D	0.15201 (3)	0.55943 (8)	0.26544 (3)	0.0191 (2)	
H2ND	0.1592 (5)	0.6113 (13)	0.2499 (4)	0.034 (4)*	
N3D	0.16401 (3)	0.58494 (8)	0.30346 (3)	0.0184 (2)	
N4D	0.19627 (3)	0.76900 (8)	0.26387 (3)	0.0187 (2)	
N5D	0.21366 (3)	0.85741 (8)	0.24885 (3)	0.0188 (2)	
H5ND	0.2330 (4)	0.9205 (13)	0.2641 (4)	0.026 (3)*	
N6D	0.22285 (3)	0.94237 (8)	0.19732 (3)	0.0190 (2)	
C1D	0.08078 (4)	0.36228 (10)	0.19044 (4)	0.0264 (3)	
H1DA	0.071232	0.354934	0.163036	0.032*	
C2D	0.06448 (4)	0.27662 (10)	0.20835 (4)	0.0261 (3)	
H2DA	0.044530	0.212155	0.193775	0.031*	
C3D	0.07822 (4)	0.28790 (10)	0.24838 (4)	0.0252 (3)	
H3DA	0.067670	0.230792	0.261699	0.030*	
C4D	0.10731 (3)	0.38255 (10)	0.26873 (4)	0.0210 (2)	
H4DA	0.117056	0.392347	0.296091	0.025*	
C5D	0.12196 (3)	0.46366 (9)	0.24760 (3)	0.0188 (2)	
C6D	0.18802 (3)	0.68536 (10)	0.31927 (3)	0.0180 (2)	
C7D	0.19845 (3)	0.70459 (10)	0.36115 (3)	0.0198 (2)	
C8D	0.20520 (3)	0.59891 (10)	0.38562 (3)	0.0232 (2)	
H8DA	0.204224	0.514904	0.375583	0.028*	
C9D	0.21331 (3)	0.61498 (11)	0.42438 (3)	0.0272 (2)	
H9DA	0.217915	0.542093	0.440712	0.033*	
C10D	0.21475 (4)	0.73719 (12)	0.43949 (4)	0.0287 (3)	
H10D	0.220206	0.748249	0.466039	0.034*	
C11D	0.20813 (4)	0.84297 (11)	0.41537 (3)	0.0306 (3)	
H11D	0.209048	0.926741	0.425535	0.037*	
C12D	0.20019 (3)	0.82762 (10)	0.37654 (3)	0.0258 (2)	
H12D	0.195938	0.900769	0.360376	0.031*	
C13D	0.20478 (3)	0.77732 (10)	0.30042 (3)	0.0188 (2)	
H13D	0.222495	0.845436	0.315455	0.023*	
C14D	0.20430 (3)	0.85119 (9)	0.20987 (3)	0.0178 (2)	
C15D	0.17704 (3)	0.75587 (10)	0.18506 (3)	0.0204 (2)	
H15D	0.164477	0.692262	0.194781	0.025*	
C16D	0.16925 (3)	0.75805 (10)	0.14632 (4)	0.0218 (2)	
H16D	0.150826	0.695776	0.128818	0.026*	
C17D	0.18838 (3)	0.85154 (10)	0.13275 (3)	0.0221 (2)	
H17D	0.183557	0.854181	0.106183	0.027*	
C18D	0.21459 (4)	0.94015 (10)	0.15945 (3)	0.0217 (2)	
H18D	0.227702	1.003999	0.150345	0.026*	

### 2-((2E)-2-{(2Z)-2-Phenyl-2-[2-(pyridin-2-yl)hydrazin-1-ylidene]ethylidene}hydrazin-1-yl)pyridine (Dhpk) Atomic displacement parameters (Å^2^)

**Table:**

	*U*^11^	*U*^22^	*U*^33^	*U*^12^	*U*^13^	*U*^23^
N1A	0.0224 (5)	0.0204 (4)	0.0189 (5)	0.0015 (3)	0.0098 (4)	−0.0002 (4)
N2A	0.0238 (5)	0.0189 (4)	0.0175 (5)	−0.0025 (3)	0.0107 (4)	−0.0008 (4)
N3A	0.0227 (5)	0.0189 (4)	0.0171 (5)	0.0004 (3)	0.0102 (4)	0.0000 (4)
N4A	0.0198 (4)	0.0181 (4)	0.0207 (5)	0.0015 (3)	0.0114 (4)	0.0025 (4)
N5A	0.0214 (5)	0.0174 (4)	0.0199 (5)	−0.0023 (3)	0.0108 (4)	0.0000 (4)
N6A	0.0214 (5)	0.0179 (4)	0.0193 (5)	0.0009 (3)	0.0094 (4)	0.0017 (4)
C1A	0.0225 (5)	0.0232 (5)	0.0191 (6)	0.0018 (4)	0.0091 (5)	−0.0018 (4)
C2A	0.0207 (5)	0.0202 (5)	0.0258 (7)	−0.0007 (4)	0.0093 (5)	−0.0036 (4)
C3A	0.0232 (6)	0.0210 (5)	0.0265 (7)	0.0001 (4)	0.0128 (5)	0.0024 (4)
C4A	0.0208 (5)	0.0216 (5)	0.0191 (6)	0.0013 (4)	0.0095 (5)	0.0015 (4)
C5A	0.0184 (5)	0.0173 (5)	0.0199 (6)	0.0025 (4)	0.0092 (5)	0.0005 (4)
C6A	0.0183 (5)	0.0180 (5)	0.0186 (6)	0.0017 (4)	0.0090 (5)	0.0004 (4)
C7A	0.0229 (5)	0.0158 (4)	0.0197 (6)	0.0012 (4)	0.0113 (5)	0.0005 (4)
C8A	0.0231 (5)	0.0272 (5)	0.0240 (6)	−0.0043 (4)	0.0122 (5)	−0.0039 (4)
C9A	0.0275 (6)	0.0310 (5)	0.0279 (6)	−0.0046 (4)	0.0180 (5)	−0.0040 (4)
C10A	0.0303 (6)	0.0244 (5)	0.0190 (6)	−0.0005 (4)	0.0134 (5)	−0.0023 (4)
C11A	0.0228 (5)	0.0223 (5)	0.0221 (6)	−0.0013 (4)	0.0089 (4)	−0.0010 (4)
C12A	0.0217 (5)	0.0204 (4)	0.0235 (6)	0.0001 (4)	0.0120 (4)	0.0010 (4)
C13A	0.0188 (5)	0.0181 (5)	0.0200 (6)	−0.0005 (4)	0.0087 (5)	−0.0001 (4)
C14A	0.0177 (5)	0.0177 (5)	0.0189 (6)	0.0035 (4)	0.0092 (5)	0.0011 (4)
C15A	0.0237 (5)	0.0196 (5)	0.0243 (7)	−0.0016 (4)	0.0133 (5)	−0.0004 (4)
C16A	0.0220 (5)	0.0222 (5)	0.0229 (6)	−0.0013 (4)	0.0103 (5)	−0.0046 (4)
C17A	0.0224 (5)	0.0233 (5)	0.0193 (6)	0.0022 (4)	0.0091 (5)	0.0005 (4)
C18A	0.0245 (6)	0.0199 (5)	0.0210 (6)	0.0011 (4)	0.0112 (5)	0.0028 (4)
N1C	0.0231 (5)	0.0220 (4)	0.0197 (6)	−0.0014 (4)	0.0104 (4)	0.0004 (4)
N2C	0.0250 (5)	0.0204 (4)	0.0166 (5)	0.0029 (4)	0.0113 (4)	0.0008 (4)
N3C	0.0223 (5)	0.0192 (4)	0.0178 (5)	−0.0006 (3)	0.0106 (4)	0.0001 (4)
N4C	0.0214 (5)	0.0177 (4)	0.0220 (5)	−0.0005 (3)	0.0126 (4)	−0.0023 (4)
N5C	0.0232 (5)	0.0184 (4)	0.0196 (5)	0.0032 (4)	0.0116 (4)	0.0007 (4)
N6C	0.0222 (5)	0.0179 (4)	0.0187 (5)	−0.0004 (3)	0.0099 (4)	−0.0010 (4)
C1C	0.0218 (5)	0.0245 (5)	0.0196 (6)	−0.0018 (4)	0.0087 (5)	0.0026 (4)
C2C	0.0211 (5)	0.0210 (5)	0.0262 (6)	−0.0001 (4)	0.0100 (5)	0.0038 (4)
C3C	0.0216 (5)	0.0207 (5)	0.0273 (6)	−0.0017 (4)	0.0136 (5)	−0.0015 (4)
C4C	0.0216 (5)	0.0213 (5)	0.0195 (6)	−0.0024 (4)	0.0108 (5)	−0.0010 (4)
C5C	0.0178 (5)	0.0180 (5)	0.0199 (6)	−0.0030 (4)	0.0086 (5)	−0.0004 (4)
C6C	0.0190 (5)	0.0202 (5)	0.0190 (6)	−0.0015 (4)	0.0096 (5)	−0.0014 (4)
C7C	0.0171 (5)	0.0242 (5)	0.0190 (6)	0.0005 (4)	0.0085 (5)	0.0005 (4)
C8C	0.0234 (5)	0.0253 (5)	0.0241 (6)	−0.0019 (4)	0.0126 (5)	−0.0020 (4)
C9C	0.0262 (6)	0.0346 (6)	0.0230 (6)	−0.0055 (4)	0.0126 (5)	−0.0071 (4)
C10C	0.0265 (6)	0.0418 (6)	0.0171 (6)	−0.0038 (5)	0.0091 (5)	0.0013 (5)
C11C	0.0342 (6)	0.0310 (6)	0.0261 (6)	0.0005 (5)	0.0137 (5)	0.0053 (5)
C12C	0.0290 (6)	0.0245 (5)	0.0229 (6)	0.0006 (4)	0.0114 (5)	0.0000 (4)
C13C	0.0214 (5)	0.0177 (5)	0.0191 (6)	0.0001 (4)	0.0093 (5)	0.0005 (4)
C14C	0.0196 (5)	0.0171 (5)	0.0204 (6)	−0.0039 (4)	0.0104 (5)	−0.0016 (4)
C15C	0.0245 (5)	0.0204 (5)	0.0223 (6)	0.0018 (4)	0.0130 (5)	0.0007 (4)
C16C	0.0251 (6)	0.0204 (5)	0.0229 (6)	0.0012 (4)	0.0110 (5)	0.0024 (4)
C17C	0.0257 (6)	0.0233 (5)	0.0171 (6)	−0.0011 (4)	0.0095 (5)	−0.0006 (4)
C18C	0.0261 (6)	0.0205 (5)	0.0217 (6)	0.0000 (4)	0.0124 (5)	−0.0022 (4)
N1B	0.0301 (5)	0.0211 (4)	0.0193 (6)	0.0013 (4)	0.0113 (4)	0.0013 (4)
N2B	0.0241 (5)	0.0188 (4)	0.0168 (5)	0.0027 (3)	0.0107 (4)	0.0011 (4)
N3B	0.0218 (5)	0.0189 (4)	0.0164 (5)	−0.0007 (3)	0.0092 (4)	−0.0004 (4)
N4B	0.0190 (4)	0.0179 (4)	0.0209 (5)	−0.0015 (3)	0.0110 (4)	−0.0024 (4)
N5B	0.0213 (5)	0.0175 (4)	0.0190 (5)	0.0019 (3)	0.0107 (4)	−0.0005 (4)
N6B	0.0204 (5)	0.0184 (4)	0.0190 (5)	−0.0004 (3)	0.0090 (4)	−0.0017 (4)
C1B	0.0336 (6)	0.0247 (5)	0.0205 (6)	0.0036 (4)	0.0105 (5)	0.0034 (4)
C2B	0.0282 (6)	0.0232 (5)	0.0273 (7)	0.0055 (4)	0.0105 (5)	0.0047 (5)
C3B	0.0274 (6)	0.0235 (5)	0.0283 (7)	0.0024 (4)	0.0144 (5)	−0.0016 (5)
C4B	0.0234 (5)	0.0235 (5)	0.0195 (6)	0.0001 (4)	0.0107 (5)	−0.0002 (4)
C5B	0.0203 (5)	0.0175 (5)	0.0199 (6)	−0.0026 (4)	0.0092 (5)	−0.0005 (4)
C6B	0.0187 (5)	0.0191 (5)	0.0176 (6)	−0.0015 (4)	0.0086 (5)	−0.0006 (4)
C7B	0.0222 (5)	0.0162 (4)	0.0183 (6)	−0.0009 (4)	0.0101 (5)	−0.0007 (4)
C8B	0.0218 (5)	0.0281 (5)	0.0225 (6)	0.0034 (4)	0.0105 (4)	0.0034 (4)
C9B	0.0263 (6)	0.0309 (5)	0.0279 (6)	0.0044 (4)	0.0171 (5)	0.0034 (4)
C10B	0.0294 (6)	0.0244 (5)	0.0187 (6)	0.0011 (4)	0.0132 (5)	0.0024 (4)
C11B	0.0233 (5)	0.0217 (5)	0.0230 (6)	0.0016 (4)	0.0094 (4)	0.0013 (4)
C12B	0.0214 (5)	0.0206 (4)	0.0226 (6)	0.0005 (4)	0.0115 (4)	−0.0001 (4)
C13B	0.0201 (5)	0.0175 (5)	0.0192 (6)	0.0000 (4)	0.0095 (5)	−0.0003 (4)
C14B	0.0171 (5)	0.0182 (5)	0.0190 (6)	−0.0036 (4)	0.0092 (5)	−0.0010 (4)
C15B	0.0236 (5)	0.0185 (5)	0.0228 (6)	0.0002 (4)	0.0134 (5)	−0.0001 (4)
C16B	0.0227 (6)	0.0221 (5)	0.0226 (6)	0.0002 (4)	0.0108 (5)	0.0033 (4)
C17B	0.0218 (5)	0.0248 (5)	0.0182 (6)	−0.0027 (4)	0.0090 (5)	−0.0010 (4)
C18B	0.0228 (5)	0.0217 (5)	0.0204 (6)	−0.0019 (4)	0.0103 (5)	−0.0032 (4)
N1D	0.0300 (5)	0.0206 (4)	0.0195 (6)	−0.0006 (4)	0.0109 (4)	−0.0005 (4)
N2D	0.0239 (5)	0.0186 (4)	0.0169 (5)	−0.0014 (3)	0.0109 (4)	−0.0002 (4)
N3D	0.0212 (5)	0.0190 (4)	0.0170 (5)	0.0013 (3)	0.0100 (4)	−0.0001 (4)
N4D	0.0194 (4)	0.0180 (4)	0.0215 (5)	0.0012 (3)	0.0115 (4)	0.0026 (4)
N5D	0.0211 (5)	0.0188 (4)	0.0186 (5)	−0.0027 (3)	0.0105 (4)	0.0006 (4)
N6D	0.0210 (5)	0.0184 (4)	0.0186 (5)	0.0014 (3)	0.0095 (4)	0.0020 (4)
C1D	0.0347 (6)	0.0227 (5)	0.0201 (6)	−0.0025 (4)	0.0104 (5)	−0.0026 (4)
C2D	0.0281 (6)	0.0217 (5)	0.0280 (7)	−0.0039 (4)	0.0116 (5)	−0.0051 (5)
C3D	0.0278 (6)	0.0217 (5)	0.0311 (7)	−0.0018 (4)	0.0173 (6)	−0.0007 (5)
C4D	0.0228 (5)	0.0209 (5)	0.0220 (6)	0.0013 (4)	0.0123 (5)	0.0005 (4)
C5D	0.0209 (5)	0.0171 (5)	0.0200 (6)	0.0025 (4)	0.0103 (5)	−0.0003 (4)
C6D	0.0180 (5)	0.0186 (5)	0.0188 (6)	0.0020 (4)	0.0093 (5)	0.0009 (4)
C7D	0.0176 (5)	0.0242 (5)	0.0183 (6)	−0.0003 (4)	0.0083 (5)	−0.0006 (4)
C8D	0.0234 (5)	0.0250 (5)	0.0230 (6)	0.0028 (4)	0.0118 (5)	0.0020 (4)
C9D	0.0256 (6)	0.0347 (6)	0.0232 (6)	0.0054 (4)	0.0123 (5)	0.0068 (4)
C10D	0.0277 (6)	0.0413 (6)	0.0171 (6)	0.0029 (5)	0.0096 (5)	−0.0013 (5)
C11D	0.0350 (6)	0.0308 (6)	0.0256 (6)	−0.0006 (5)	0.0128 (5)	−0.0064 (5)
C12D	0.0294 (6)	0.0244 (5)	0.0246 (6)	−0.0013 (4)	0.0127 (5)	−0.0007 (4)
C13D	0.0190 (5)	0.0191 (5)	0.0186 (6)	−0.0004 (4)	0.0084 (5)	0.0000 (4)
C14D	0.0175 (5)	0.0169 (5)	0.0201 (6)	0.0036 (4)	0.0092 (5)	0.0013 (4)
C15D	0.0227 (5)	0.0188 (5)	0.0228 (6)	−0.0005 (4)	0.0127 (5)	0.0000 (4)
C16D	0.0232 (6)	0.0201 (5)	0.0227 (6)	−0.0008 (4)	0.0105 (5)	−0.0027 (4)
C17D	0.0262 (6)	0.0229 (5)	0.0179 (6)	0.0013 (4)	0.0101 (5)	0.0013 (4)
C18D	0.0249 (6)	0.0199 (5)	0.0223 (6)	0.0003 (4)	0.0120 (5)	0.0031 (4)

### 2-((2E)-2-{(2Z)-2-Phenyl-2-[2-(pyridin-2-yl)hydrazin-1-ylidene]ethylidene}hydrazin-1-yl)pyridine (Dhpk) Geometric parameters (Å, º)

**Table:**

N1A—C5A	1.3349 (15)	N1B—C5B	1.3338 (15)
N1A—C1A	1.3424 (14)	N1B—C1B	1.3423 (15)
N2A—N3A	1.3410 (13)	N2B—N3B	1.3405 (13)
N2A—C5A	1.3861 (14)	N2B—C5B	1.3869 (14)
N2A—H2NA	0.896 (15)	N2B—H2NB	0.875 (15)
N3A—C6A	1.3085 (14)	N3B—C6B	1.3095 (13)
N4A—C13A	1.2936 (15)	N4B—C13B	1.2907 (15)
N4A—N5A	1.3530 (12)	N4B—N5B	1.3523 (12)
N5A—C14A	1.3768 (15)	N5B—C14B	1.3743 (15)
N5A—H5NA	0.916 (14)	N5B—H5NB	0.912 (15)
N6A—C18A	1.3410 (15)	N6B—C18B	1.3404 (15)
N6A—C14A	1.3433 (13)	N6B—C14B	1.3458 (13)
C1A—C2A	1.3857 (15)	C1B—C2B	1.3817 (16)
C1A—H1AA	0.9500	C1B—H1BA	0.9500
C2A—C3A	1.3914 (17)	C2B—C3B	1.3883 (17)
C2A—H2AA	0.9500	C2B—H2BA	0.9500
C3A—C4A	1.3817 (15)	C3B—C4B	1.3816 (16)
C3A—H3AA	0.9500	C3B—H3BA	0.9500
C4A—C5A	1.4050 (14)	C4B—C5B	1.4028 (15)
C4A—H4AA	0.9500	C4B—H4BA	0.9500
C6A—C13A	1.4586 (14)	C6B—C13B	1.4596 (14)
C6A—C7A	1.4884 (16)	C6B—C7B	1.4875 (15)
C7A—C12A	1.3939 (15)	C7B—C12B	1.3945 (15)
C7A—C8A	1.4024 (14)	C7B—C8B	1.4039 (14)
C8A—C9A	1.3820 (14)	C8B—C9B	1.3820 (15)
C8A—H8AA	0.9500	C8B—H8BA	0.9500
C9A—C10A	1.3929 (16)	C9B—C10B	1.3905 (16)
C9A—H9AA	0.9500	C9B—H9BA	0.9500
C10A—C11A	1.3848 (14)	C10B—C11B	1.3855 (14)
C10A—H10A	0.9500	C10B—H10B	0.9500
C11A—C12A	1.3901 (14)	C11B—C12B	1.3910 (14)
C11A—H11A	0.9500	C11B—H11B	0.9500
C12A—H12A	0.9500	C12B—H12B	0.9500
C13A—H13A	0.9500	C13B—H13B	0.9500
C14A—C15A	1.4092 (15)	C14B—C15B	1.4115 (15)
C15A—C16A	1.3776 (17)	C15B—C16B	1.3770 (17)
C15A—H15A	0.9500	C15B—H15B	0.9500
C16A—C17A	1.3951 (15)	C16B—C17B	1.3961 (15)
C16A—H16A	0.9500	C16B—H16B	0.9500
C17A—C18A	1.3823 (16)	C17B—C18B	1.3817 (16)
C17A—H17A	0.9500	C17B—H17B	0.9500
C18A—H18A	0.9500	C18B—H18B	0.9500
N1C—C5C	1.3368 (15)	N1D—C5D	1.3363 (15)
N1C—C1C	1.3441 (14)	N1D—C1D	1.3397 (15)
N2C—N3C	1.3452 (13)	N2D—N3D	1.3461 (13)
N2C—C5C	1.3843 (14)	N2D—C5D	1.3869 (14)
N2C—H2NC	0.907 (15)	N2D—H2ND	0.909 (15)
N3C—C6C	1.3106 (14)	N3D—C6D	1.3088 (14)
N4C—C13C	1.2910 (15)	N4D—C13D	1.2925 (15)
N4C—N5C	1.3541 (12)	N4D—N5D	1.3530 (12)
N5C—C14C	1.3753 (15)	N5D—C14D	1.3747 (15)
N5C—H5NC	0.910 (14)	N5D—H5ND	0.938 (14)
N6C—C18C	1.3416 (15)	N6D—C18D	1.3410 (15)
N6C—C14C	1.3449 (13)	N6D—C14D	1.3432 (13)
C1C—C2C	1.3855 (15)	C1D—C2D	1.3811 (16)
C1C—H1CA	0.9500	C1D—H1DA	0.9500
C2C—C3C	1.3897 (17)	C2D—C3D	1.3889 (18)
C2C—H2CA	0.9500	C2D—H2DA	0.9500
C3C—C4C	1.3816 (15)	C3D—C4D	1.3814 (16)
C3C—H3CA	0.9500	C3D—H3DA	0.9500
C4C—C5C	1.4057 (14)	C4D—C5D	1.4021 (15)
C4C—H4CA	0.9500	C4D—H4DA	0.9500
C6C—C13C	1.4574 (14)	C6D—C13D	1.4576 (14)
C6C—C7C	1.4861 (16)	C6D—C7D	1.4860 (16)
C7C—C8C	1.3950 (15)	C7D—C8D	1.3948 (15)
C7C—C12C	1.3986 (15)	C7D—C12D	1.4002 (15)
C8C—C9C	1.3862 (15)	C8D—C9D	1.3856 (15)
C8C—H8CA	0.9500	C8D—H8DA	0.9500
C9C—C10C	1.3870 (16)	C9D—C10D	1.3896 (16)
C9C—H9CA	0.9500	C9D—H9DA	0.9500
C10C—C11C	1.3925 (17)	C10D—C11D	1.3884 (17)
C10C—H10C	0.9500	C10D—H10D	0.9500
C11C—C12C	1.3885 (15)	C11D—C12D	1.3887 (15)
C11C—H11C	0.9500	C11D—H11D	0.9500
C12C—H12C	0.9500	C12D—H12D	0.9500
C13C—H13C	0.9500	C13D—H13D	0.9500
C14C—C15C	1.4105 (15)	C14D—C15D	1.4122 (15)
C15C—C16C	1.3770 (17)	C15D—C16D	1.3778 (17)
C15C—H15C	0.9500	C15D—H15D	0.9500
C16C—C17C	1.3946 (15)	C16D—C17D	1.3947 (15)
C16C—H16C	0.9500	C16D—H16D	0.9500
C17C—C18C	1.3818 (16)	C17D—C18D	1.3811 (16)
C17C—H17C	0.9500	C17D—H17D	0.9500
C18C—H18C	0.9500	C18D—H18D	0.9500
			
C5A—N1A—C1A	116.65 (9)	C5B—N1B—C1B	116.52 (10)
N3A—N2A—C5A	118.79 (9)	N3B—N2B—C5B	118.77 (9)
N3A—N2A—H2NA	123.1 (10)	N3B—N2B—H2NB	121.8 (10)
C5A—N2A—H2NA	118.0 (10)	C5B—N2B—H2NB	118.9 (10)
C6A—N3A—N2A	120.00 (9)	C6B—N3B—N2B	120.11 (9)
C13A—N4A—N5A	118.44 (9)	C13B—N4B—N5B	118.39 (9)
N4A—N5A—C14A	118.72 (9)	N4B—N5B—C14B	118.73 (9)
N4A—N5A—H5NA	122.9 (8)	N4B—N5B—H5NB	122.7 (9)
C14A—N5A—H5NA	118.3 (8)	C14B—N5B—H5NB	118.5 (9)
C18A—N6A—C14A	117.18 (9)	C18B—N6B—C14B	117.32 (9)
N1A—C1A—C2A	124.09 (11)	N1B—C1B—C2B	124.31 (11)
N1A—C1A—H1AA	118.0	N1B—C1B—H1BA	117.8
C2A—C1A—H1AA	118.0	C2B—C1B—H1BA	117.8
C1A—C2A—C3A	118.02 (10)	C1B—C2B—C3B	117.93 (11)
C1A—C2A—H2AA	121.0	C1B—C2B—H2BA	121.0
C3A—C2A—H2AA	121.0	C3B—C2B—H2BA	121.0
C4A—C3A—C2A	119.59 (10)	C4B—C3B—C2B	119.63 (10)
C4A—C3A—H3AA	120.2	C4B—C3B—H3BA	120.2
C2A—C3A—H3AA	120.2	C2B—C3B—H3BA	120.2
C3A—C4A—C5A	117.51 (11)	C3B—C4B—C5B	117.55 (11)
C3A—C4A—H4AA	121.2	C3B—C4B—H4BA	121.2
C5A—C4A—H4AA	121.2	C5B—C4B—H4BA	121.2
N1A—C5A—N2A	114.24 (9)	N1B—C5B—N2B	114.17 (9)
N1A—C5A—C4A	124.07 (10)	N1B—C5B—C4B	124.04 (10)
N2A—C5A—C4A	121.67 (10)	N2B—C5B—C4B	121.79 (11)
N3A—C6A—C13A	127.09 (10)	N3B—C6B—C13B	127.08 (10)
N3A—C6A—C7A	113.45 (9)	N3B—C6B—C7B	113.50 (9)
C13A—C6A—C7A	119.40 (9)	C13B—C6B—C7B	119.34 (9)
C12A—C7A—C8A	118.34 (10)	C12B—C7B—C8B	118.26 (10)
C12A—C7A—C6A	122.57 (9)	C12B—C7B—C6B	122.65 (9)
C8A—C7A—C6A	119.08 (10)	C8B—C7B—C6B	119.08 (10)
C9A—C8A—C7A	120.90 (10)	C9B—C8B—C7B	120.81 (10)
C9A—C8A—H8AA	119.5	C9B—C8B—H8BA	119.6
C7A—C8A—H8AA	119.5	C7B—C8B—H8BA	119.6
C8A—C9A—C10A	120.17 (9)	C8B—C9B—C10B	120.34 (9)
C8A—C9A—H9AA	119.9	C8B—C9B—H9BA	119.8
C10A—C9A—H9AA	119.9	C10B—C9B—H9BA	119.8
C11A—C10A—C9A	119.48 (10)	C11B—C10B—C9B	119.49 (10)
C11A—C10A—H10A	120.3	C11B—C10B—H10B	120.3
C9A—C10A—H10A	120.3	C9B—C10B—H10B	120.3
C10A—C11A—C12A	120.43 (10)	C10B—C11B—C12B	120.33 (10)
C10A—C11A—H11A	119.8	C10B—C11B—H11B	119.8
C12A—C11A—H11A	119.8	C12B—C11B—H11B	119.8
C11A—C12A—C7A	120.67 (9)	C11B—C12B—C7B	120.73 (9)
C11A—C12A—H12A	119.7	C11B—C12B—H12B	119.6
C7A—C12A—H12A	119.7	C7B—C12B—H12B	119.6
N4A—C13A—C6A	121.26 (10)	N4B—C13B—C6B	121.53 (10)
N4A—C13A—H13A	119.4	N4B—C13B—H13B	119.2
C6A—C13A—H13A	119.4	C6B—C13B—H13B	119.2
N6A—C14A—N5A	114.93 (9)	N6B—C14B—N5B	115.01 (9)
N6A—C14A—C15A	122.82 (10)	N6B—C14B—C15B	122.54 (10)
N5A—C14A—C15A	122.25 (9)	N5B—C14B—C15B	122.45 (9)
C16A—C15A—C14A	117.97 (10)	C16B—C15B—C14B	118.18 (10)
C16A—C15A—H15A	121.0	C16B—C15B—H15B	120.9
C14A—C15A—H15A	121.0	C14B—C15B—H15B	120.9
C15A—C16A—C17A	120.21 (11)	C15B—C16B—C17B	120.03 (11)
C15A—C16A—H16A	119.9	C15B—C16B—H16B	120.0
C17A—C16A—H16A	119.9	C17B—C16B—H16B	120.0
C18A—C17A—C16A	117.17 (11)	C18B—C17B—C16B	117.35 (11)
C18A—C17A—H17A	121.4	C18B—C17B—H17B	121.3
C16A—C17A—H17A	121.4	C16B—C17B—H17B	121.3
N6A—C18A—C17A	124.63 (10)	N6B—C18B—C17B	124.56 (10)
N6A—C18A—H18A	117.7	N6B—C18B—H18B	117.7
C17A—C18A—H18A	117.7	C17B—C18B—H18B	117.7
C5C—N1C—C1C	116.57 (9)	C5D—N1D—C1D	116.63 (10)
N3C—N2C—C5C	119.27 (9)	N3D—N2D—C5D	119.03 (9)
N3C—N2C—H2NC	121.7 (10)	N3D—N2D—H2ND	122.3 (9)
C5C—N2C—H2NC	119.0 (10)	C5D—N2D—H2ND	117.8 (9)
C6C—N3C—N2C	119.42 (9)	C6D—N3D—N2D	119.39 (9)
C13C—N4C—N5C	118.12 (9)	C13D—N4D—N5D	118.11 (9)
N4C—N5C—C14C	118.68 (9)	N4D—N5D—C14D	118.80 (9)
N4C—N5C—H5NC	122.8 (9)	N4D—N5D—H5ND	123.0 (8)
C14C—N5C—H5NC	118.5 (9)	C14D—N5D—H5ND	118.1 (8)
C18C—N6C—C14C	117.21 (9)	C18D—N6D—C14D	117.13 (9)
N1C—C1C—C2C	124.33 (11)	N1D—C1D—C2D	124.47 (12)
N1C—C1C—H1CA	117.8	N1D—C1D—H1DA	117.8
C2C—C1C—H1CA	117.8	C2D—C1D—H1DA	117.8
C1C—C2C—C3C	117.80 (10)	C1D—C2D—C3D	117.76 (11)
C1C—C2C—H2CA	121.1	C1D—C2D—H2DA	121.1
C3C—C2C—H2CA	121.1	C3D—C2D—H2DA	121.1
C4C—C3C—C2C	119.75 (10)	C4D—C3D—C2D	119.68 (10)
C4C—C3C—H3CA	120.1	C4D—C3D—H3DA	120.2
C2C—C3C—H3CA	120.1	C2D—C3D—H3DA	120.2
C3C—C4C—C5C	117.66 (11)	C3D—C4D—C5D	117.69 (11)
C3C—C4C—H4CA	121.2	C3D—C4D—H4DA	121.2
C5C—C4C—H4CA	121.2	C5D—C4D—H4DA	121.2
N1C—C5C—N2C	114.21 (9)	N1D—C5D—N2D	114.07 (9)
N1C—C5C—C4C	123.85 (10)	N1D—C5D—C4D	123.76 (10)
N2C—C5C—C4C	121.92 (10)	N2D—C5D—C4D	122.16 (11)
N3C—C6C—C13C	127.19 (10)	N3D—C6D—C13D	127.41 (10)
N3C—C6C—C7C	114.31 (9)	N3D—C6D—C7D	114.32 (9)
C13C—C6C—C7C	118.50 (9)	C13D—C6D—C7D	118.27 (9)
C8C—C7C—C12C	118.83 (10)	C8D—C7D—C12D	118.73 (10)
C8C—C7C—C6C	120.03 (10)	C8D—C7D—C6D	120.05 (9)
C12C—C7C—C6C	121.11 (10)	C12D—C7D—C6D	121.18 (10)
C9C—C8C—C7C	120.58 (10)	C9D—C8D—C7D	120.79 (10)
C9C—C8C—H8CA	119.7	C9D—C8D—H8DA	119.6
C7C—C8C—H8CA	119.7	C7D—C8D—H8DA	119.6
C8C—C9C—C10C	120.60 (10)	C8D—C9D—C10D	120.31 (10)
C8C—C9C—H9CA	119.7	C8D—C9D—H9DA	119.8
C10C—C9C—H9CA	119.7	C10D—C9D—H9DA	119.8
C9C—C10C—C11C	119.15 (11)	C11D—C10D—C9D	119.30 (11)
C9C—C10C—H10C	120.4	C11D—C10D—H10D	120.3
C11C—C10C—H10C	120.4	C9D—C10D—H10D	120.3
C12C—C11C—C10C	120.59 (10)	C10D—C11D—C12D	120.70 (10)
C12C—C11C—H11C	119.7	C10D—C11D—H11D	119.7
C10C—C11C—H11C	119.7	C12D—C11D—H11D	119.7
C11C—C12C—C7C	120.25 (10)	C11D—C12D—C7D	120.16 (10)
C11C—C12C—H12C	119.9	C11D—C12D—H12D	119.9
C7C—C12C—H12C	119.9	C7D—C12D—H12D	119.9
N4C—C13C—C6C	121.91 (10)	N4D—C13D—C6D	121.99 (10)
N4C—C13C—H13C	119.0	N4D—C13D—H13D	119.0
C6C—C13C—H13C	119.0	C6D—C13D—H13D	119.0
N6C—C14C—N5C	114.91 (10)	N6D—C14D—N5D	115.04 (9)
N6C—C14C—C15C	122.81 (10)	N6D—C14D—C15D	122.89 (10)
N5C—C14C—C15C	122.27 (9)	N5D—C14D—C15D	122.07 (9)
C16C—C15C—C14C	117.97 (10)	C16D—C15D—C14D	117.89 (10)
C16C—C15C—H15C	121.0	C16D—C15D—H15D	121.1
C14C—C15C—H15C	121.0	C14D—C15D—H15D	121.1
C15C—C16C—C17C	120.13 (11)	C15D—C16D—C17D	120.10 (11)
C15C—C16C—H16C	119.9	C15D—C16D—H16D	119.9
C17C—C16C—H16C	119.9	C17D—C16D—H16D	119.9
C18C—C17C—C16C	117.43 (11)	C18D—C17D—C16D	117.40 (11)
C18C—C17C—H17C	121.3	C18D—C17D—H17D	121.3
C16C—C17C—H17C	121.3	C16D—C17D—H17D	121.3
N6C—C18C—C17C	124.45 (10)	N6D—C18D—C17D	124.60 (10)
N6C—C18C—H18C	117.8	N6D—C18D—H18D	117.7
C17C—C18C—H18C	117.8	C17D—C18D—H18D	117.7
			
C5A—N2A—N3A—C6A	177.26 (9)	C5B—N2B—N3B—C6B	172.83 (9)
C13A—N4A—N5A—C14A	174.47 (9)	C13B—N4B—N5B—C14B	174.28 (9)
C5A—N1A—C1A—C2A	−0.80 (15)	C5B—N1B—C1B—C2B	0.01 (17)
N1A—C1A—C2A—C3A	−1.35 (16)	N1B—C1B—C2B—C3B	−0.82 (18)
C1A—C2A—C3A—C4A	1.52 (15)	C1B—C2B—C3B—C4B	0.46 (17)
C2A—C3A—C4A—C5A	0.32 (15)	C2B—C3B—C4B—C5B	0.60 (16)
C1A—N1A—C5A—N2A	−175.71 (9)	C1B—N1B—C5B—N2B	−178.38 (9)
C1A—N1A—C5A—C4A	2.87 (15)	C1B—N1B—C5B—C4B	1.17 (16)
N3A—N2A—C5A—N1A	−179.41 (9)	N3B—N2B—C5B—N1B	−174.84 (9)
N3A—N2A—C5A—C4A	1.97 (15)	N3B—N2B—C5B—C4B	5.60 (15)
C3A—C4A—C5A—N1A	−2.67 (16)	C3B—C4B—C5B—N1B	−1.49 (16)
C3A—C4A—C5A—N2A	175.81 (9)	C3B—C4B—C5B—N2B	178.03 (10)
N2A—N3A—C6A—C13A	0.21 (16)	N2B—N3B—C6B—C13B	−0.41 (16)
N2A—N3A—C6A—C7A	−177.11 (9)	N2B—N3B—C6B—C7B	−176.96 (9)
N3A—C6A—C7A—C12A	−146.48 (10)	N3B—C6B—C7B—C12B	−145.60 (10)
C13A—C6A—C7A—C12A	35.98 (14)	C13B—C6B—C7B—C12B	37.56 (14)
N3A—C6A—C7A—C8A	32.23 (13)	N3B—C6B—C7B—C8B	33.43 (13)
C13A—C6A—C7A—C8A	−145.31 (10)	C13B—C6B—C7B—C8B	−143.41 (10)
C12A—C7A—C8A—C9A	0.71 (15)	C12B—C7B—C8B—C9B	0.86 (15)
C6A—C7A—C8A—C9A	−178.05 (9)	C6B—C7B—C8B—C9B	−178.22 (10)
C7A—C8A—C9A—C10A	0.36 (16)	C7B—C8B—C9B—C10B	0.53 (16)
C8A—C9A—C10A—C11A	−0.70 (16)	C8B—C9B—C10B—C11B	−0.89 (16)
C9A—C10A—C11A—C12A	−0.03 (15)	C9B—C10B—C11B—C12B	−0.16 (15)
C10A—C11A—C12A—C7A	1.13 (15)	C10B—C11B—C12B—C7B	1.58 (15)
C8A—C7A—C12A—C11A	−1.45 (15)	C8B—C7B—C12B—C11B	−1.91 (15)
C6A—C7A—C12A—C11A	177.27 (9)	C6B—C7B—C12B—C11B	177.13 (9)
N5A—N4A—C13A—C6A	−177.24 (9)	N5B—N4B—C13B—C6B	−176.70 (9)
N3A—C6A—C13A—N4A	0.04 (17)	N3B—C6B—C13B—N4B	0.42 (17)
C7A—C6A—C13A—N4A	177.22 (9)	C7B—C6B—C13B—N4B	176.78 (9)
C18A—N6A—C14A—N5A	178.66 (9)	C18B—N6B—C14B—N5B	178.20 (9)
C18A—N6A—C14A—C15A	−1.23 (15)	C18B—N6B—C14B—C15B	−1.52 (14)
N4A—N5A—C14A—N6A	−177.66 (8)	N4B—N5B—C14B—N6B	−177.33 (8)
N4A—N5A—C14A—C15A	2.22 (14)	N4B—N5B—C14B—C15B	2.39 (14)
N6A—C14A—C15A—C16A	1.32 (15)	N6B—C14B—C15B—C16B	1.73 (15)
N5A—C14A—C15A—C16A	−178.56 (10)	N5B—C14B—C15B—C16B	−177.97 (10)
C14A—C15A—C16A—C17A	−0.25 (15)	C14B—C15B—C16B—C17B	−0.50 (15)
C15A—C16A—C17A—C18A	−0.79 (15)	C15B—C16B—C17B—C18B	−0.79 (15)
C14A—N6A—C18A—C17A	0.08 (15)	C14B—N6B—C18B—C17B	0.10 (15)
C16A—C17A—C18A—N6A	0.91 (16)	C16B—C17B—C18B—N6B	1.05 (16)
C5C—N2C—N3C—C6C	−175.81 (9)	C5D—N2D—N3D—C6D	−171.33 (9)
C13C—N4C—N5C—C14C	−178.81 (9)	C13D—N4D—N5D—C14D	−178.87 (9)
C5C—N1C—C1C—C2C	1.05 (15)	C5D—N1D—C1D—C2D	−0.22 (17)
N1C—C1C—C2C—C3C	0.50 (16)	N1D—C1D—C2D—C3D	0.41 (18)
C1C—C2C—C3C—C4C	−1.04 (15)	C1D—C2D—C3D—C4D	−0.12 (16)
C2C—C3C—C4C—C5C	0.07 (15)	C2D—C3D—C4D—C5D	−0.32 (16)
C1C—N1C—C5C—N2C	176.37 (9)	C1D—N1D—C5D—N2D	178.63 (9)
C1C—N1C—C5C—C4C	−2.13 (15)	C1D—N1D—C5D—C4D	−0.27 (15)
N3C—N2C—C5C—N1C	178.21 (9)	N3D—N2D—C5D—N1D	173.57 (9)
N3C—N2C—C5C—C4C	−3.25 (15)	N3D—N2D—C5D—C4D	−7.50 (14)
C3C—C4C—C5C—N1C	1.61 (15)	C3D—C4D—C5D—N1D	0.54 (16)
C3C—C4C—C5C—N2C	−176.78 (9)	C3D—C4D—C5D—N2D	−178.28 (9)
N2C—N3C—C6C—C13C	−1.21 (16)	N2D—N3D—C6D—C13D	−0.75 (16)
N2C—N3C—C6C—C7C	179.40 (9)	N2D—N3D—C6D—C7D	178.94 (8)
N3C—C6C—C7C—C8C	34.86 (14)	N3D—C6D—C7D—C8D	35.53 (14)
C13C—C6C—C7C—C8C	−144.59 (10)	C13D—C6D—C7D—C8D	−144.75 (10)
N3C—C6C—C7C—C12C	−143.14 (10)	N3D—C6D—C7D—C12D	−142.18 (10)
C13C—C6C—C7C—C12C	37.42 (14)	C13D—C6D—C7D—C12D	37.55 (14)
C12C—C7C—C8C—C9C	0.19 (15)	C12D—C7D—C8D—C9D	0.27 (15)
C6C—C7C—C8C—C9C	−177.85 (10)	C6D—C7D—C8D—C9D	−177.49 (9)
C7C—C8C—C9C—C10C	−0.03 (16)	C7D—C8D—C9D—C10D	0.14 (16)
C8C—C9C—C10C—C11C	0.10 (17)	C8D—C9D—C10D—C11D	−0.24 (17)
C9C—C10C—C11C—C12C	−0.32 (17)	C9D—C10D—C11D—C12D	−0.08 (17)
C10C—C11C—C12C—C7C	0.49 (17)	C10D—C11D—C12D—C7D	0.49 (17)
C8C—C7C—C12C—C11C	−0.41 (16)	C8D—C7D—C12D—C11D	−0.58 (16)
C6C—C7C—C12C—C11C	177.61 (10)	C6D—C7D—C12D—C11D	177.16 (10)
N5C—N4C—C13C—C6C	−178.77 (9)	N5D—N4D—C13D—C6D	−179.07 (9)
N3C—C6C—C13C—N4C	1.58 (17)	N3D—C6D—C13D—N4D	1.41 (17)
C7C—C6C—C13C—N4C	−179.05 (9)	C7D—C6D—C13D—N4D	−178.27 (9)
C18C—N6C—C14C—N5C	179.75 (9)	C18D—N6D—C14D—N5D	−179.94 (9)
C18C—N6C—C14C—C15C	−0.18 (15)	C18D—N6D—C14D—C15D	−0.16 (15)
N4C—N5C—C14C—N6C	−179.19 (8)	N4D—N5D—C14D—N6D	−179.99 (8)
N4C—N5C—C14C—C15C	0.75 (15)	N4D—N5D—C14D—C15D	0.23 (14)
N6C—C14C—C15C—C16C	−0.02 (16)	N6D—C14D—C15D—C16D	−0.32 (15)
N5C—C14C—C15C—C16C	−179.94 (10)	N5D—C14D—C15D—C16D	179.44 (10)
C14C—C15C—C16C—C17C	0.43 (16)	C14D—C15D—C16D—C17D	0.64 (15)
C15C—C16C—C17C—C18C	−0.63 (16)	C15D—C16D—C17D—C18D	−0.48 (16)
C14C—N6C—C18C—C17C	−0.04 (16)	C14D—N6D—C18D—C17D	0.34 (15)
C16C—C17C—C18C—N6C	0.44 (16)	C16D—C17D—C18D—N6D	−0.02 (16)

### 2-((2E)-2-{(2Z)-2-Phenyl-2-[2-(pyridin-2-yl)hydrazin-1-ylidene]ethylidene}hydrazin-1-yl)pyridine (Dhpk) Hydrogen-bond geometry (Å, º)

**Table:**

*D*—H···*A*	*D*—H	H···*A*	*D*···*A*	*D*—H···*A*
N2*A*—H2*NA*···N4*A*	0.897 (17)	2.018 (16)	2.6687 (14)	128.3 (14)
N5*A*—H5*NA*···N6*D*	0.916 (14)	2.126 (14)	3.0401 (14)	175.9 (14)
N2*C*—H2*NC*···N4*C*	0.906 (17)	1.995 (16)	2.6742 (13)	130.6 (14)
N5*C*—H5*NC*···N6*B*	0.911 (14)	2.129 (14)	3.0390 (14)	177.0 (14)
N2*B*—H2*NB*···N4*B*	0.877 (17)	2.026 (16)	2.6784 (14)	130.4 (15)
N5*B*—H5*NB*···N6*C*	0.913 (15)	2.126 (14)	3.0371 (14)	175.4 (12)
N2*D*—H2*ND*···N4*D*	0.911 (15)	2.008 (15)	2.6810 (13)	129.5 (12)
N5*D*—H5*ND*···N6*A*	0.938 (14)	2.102 (14)	3.0388 (14)	177.5 (10)
