# Supplementary material for: Some thio­ether-ketones and their related derivatives
Source: Acta Crystallogr E Crystallogr Commun. 2025 May 13;81(Pt 6):520–9. doi: 10.1107/S2056989025004037 (PMC12142416; doi:10.1107/S2056989025004037)
Supplement: Supplementary file 17 [file e-81-00520-sup18.docx]

**Supplementary Information, Part-II**

**Some thioether-ketones and their related derivatives.**

Molly A. O'Connor^a^, Raymond J. Butcher^b^, Vitaly V. Pavlishchuk^c^*, Anthony W. Addison^a^ and Anna V. Pavlishchuk^c^

**
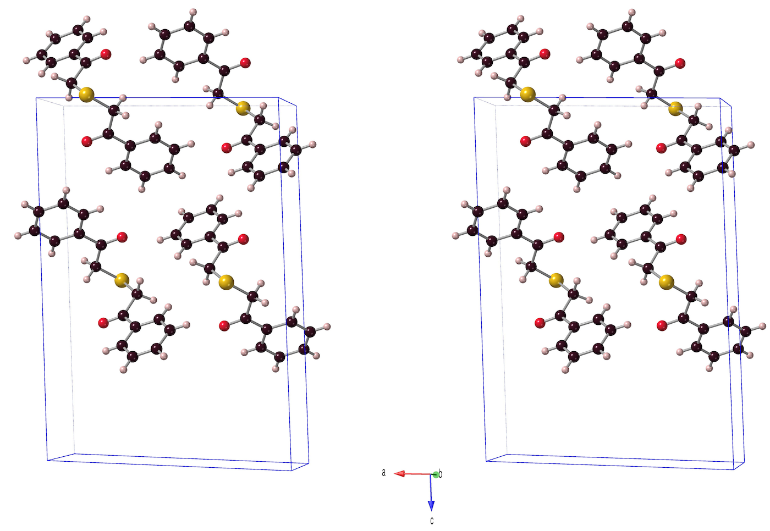
**

**Figure S1:** Molecular cell of Mtdp (ball/stick model, inverse stereoview along the *b*-direction).


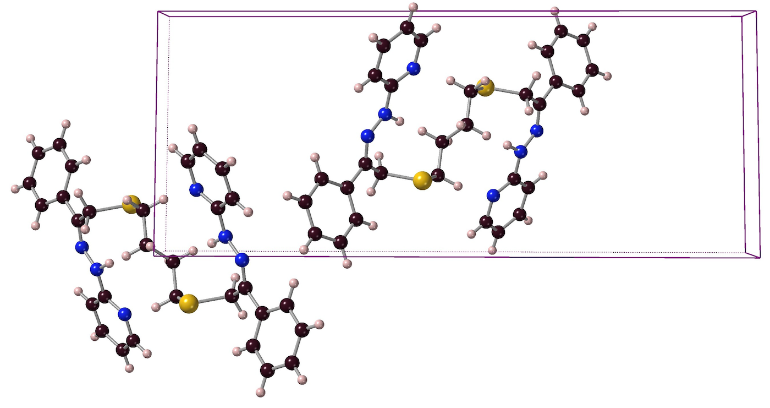


**Figure S2:** Molecular cell of Prpsb, viewed along the *a*-direction, with the *c*-axis vertical (ball & stick model).


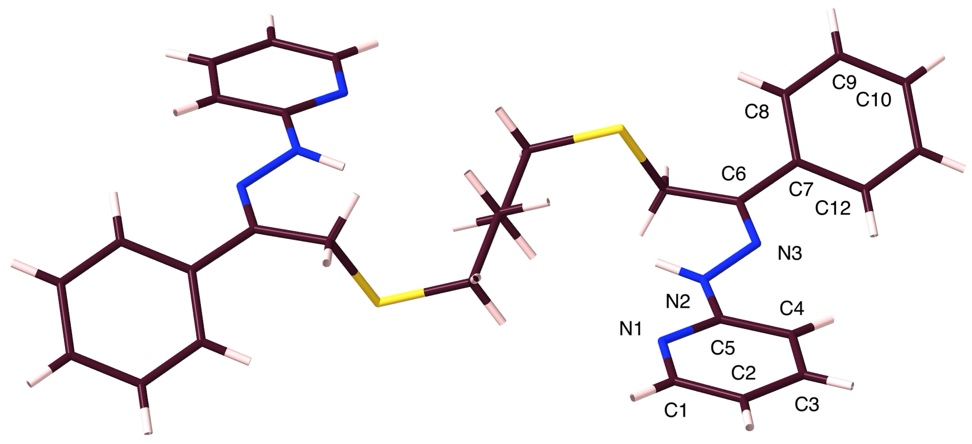


(a) Reference diagram.


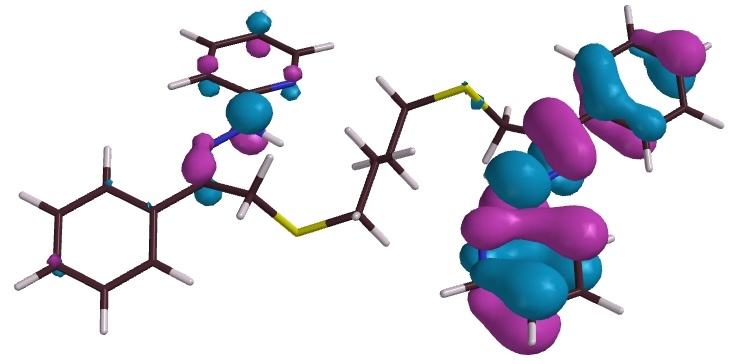


(b) HOMO(-1)


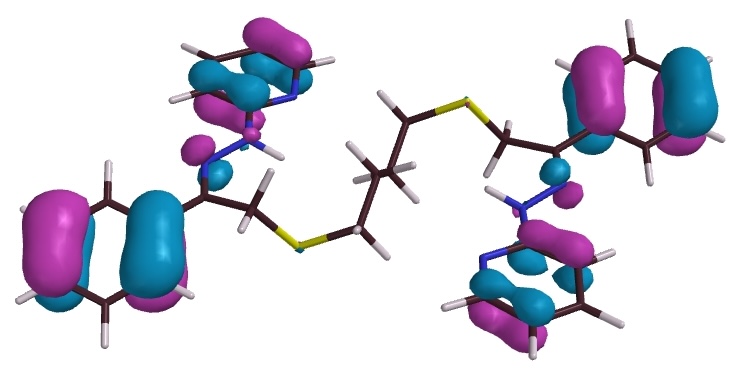


(c) HOMO(-6)


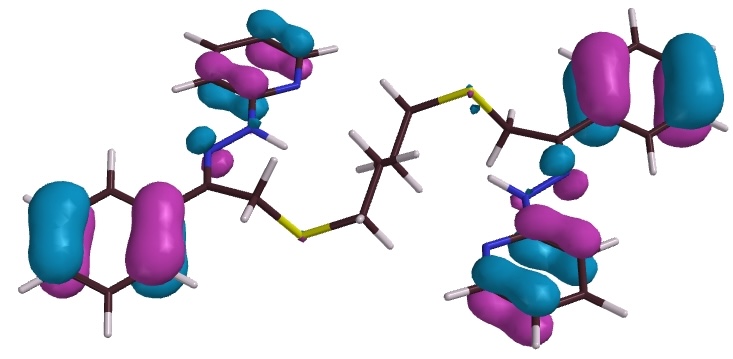


(d) HOMO(-7)

**Figure** **S3:** Further MO surfaces for Prpsb, illustrating components of the pyridylhydrazones' π-bonding system.


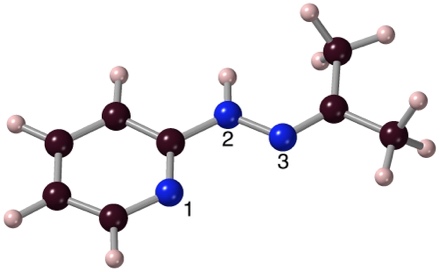

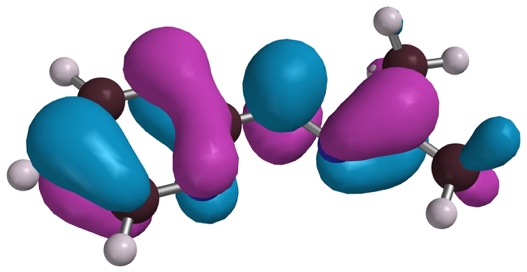


(a) (b)


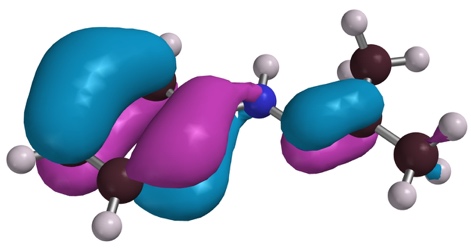

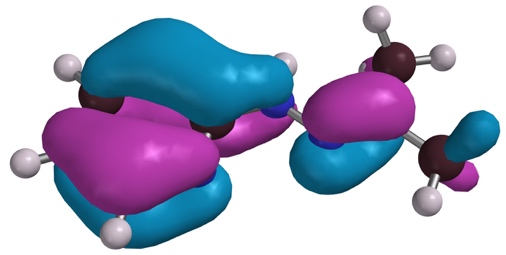


(c) (d)

**Figure S4:** MO surfaces of an acetone-pyridylhydrazone model, showing the through-conjugation in this model system. (a) Reference diagram; (b) the HOMO, which π-bonds the acetone-imine-C to the imine-N3; (c) the HOMO[–2], which lacks any π-bond between the imine-N3 and the hydrazine-N2; (d) the HOMO[–4], which π-bonds the hydrazine-N2 to the pyridine-N1 through the pyridine C.

**
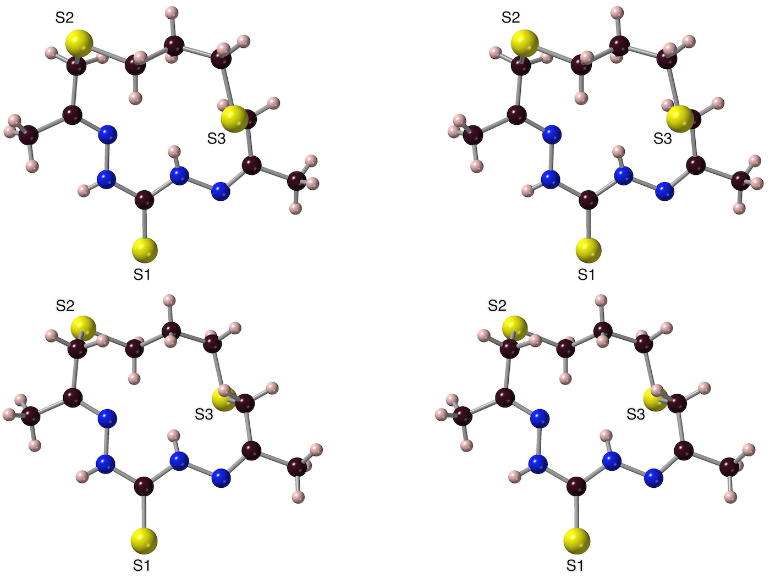
**

**Figure S5:** Inverse stereoviews of the two enantiomers (upper & lower pairs) of Ctrsp , viewed along the lattice *c*-direction. With the C–S1 bond pointing downward and the *exo* N–H bond on the left, then in the upper part of Fig. S5, their trimethylene linkages are oriented *toward* the viewer, while in the other conformer (the lower diagram), they are *behind* the remainder of the molecule.

**
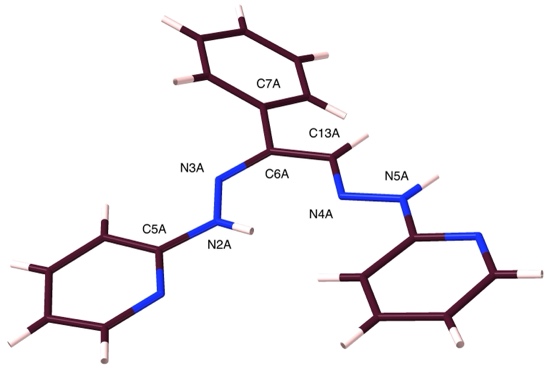
**

(a) Reference framework


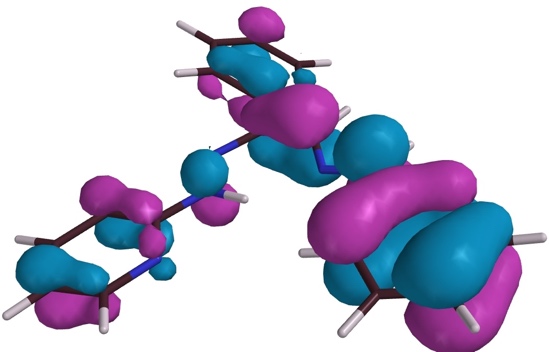

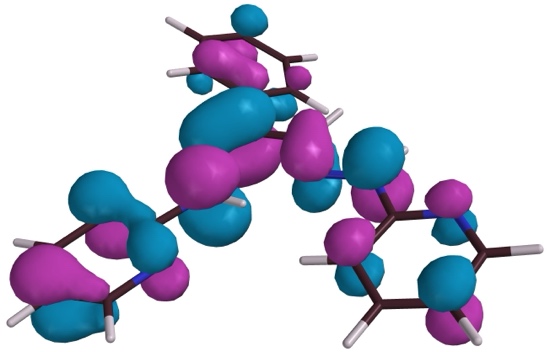


HOMO HOMO(-1)


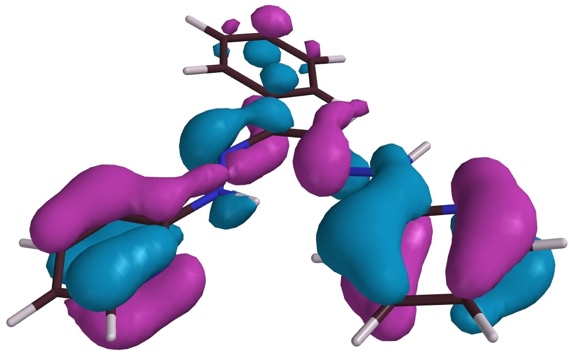

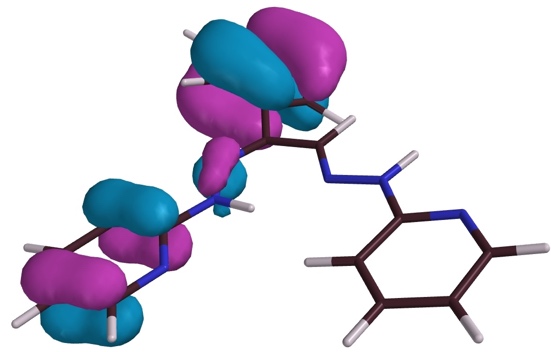


HOMO(–2) HOMO(–6)


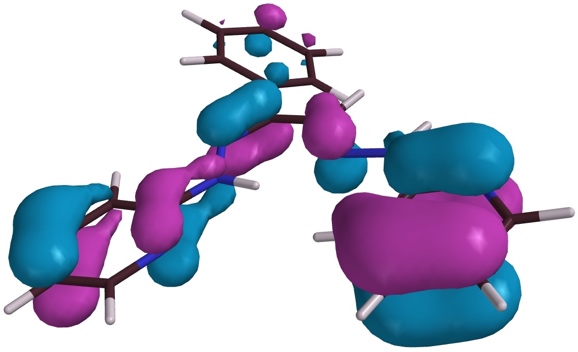

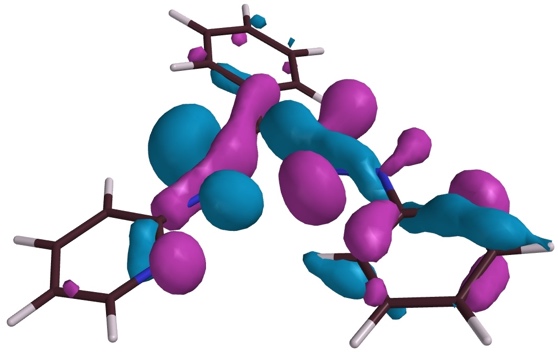


HOMO(–7) HOMO(–9)

**Figure S6:** MO surfaces for Dhpk: Note the π-bonding contributions N3–C6, N4–C13 for the HOMO; C6–C13 for HOMO(–1); N2–N3 for HOMO(–2); N3–C6, N4–C13, N5–C14, N2–C5 for HOMO(–6); C7–C6–N3–N2–C5 for HOMO(–7); N3–C6, N2–C5, N4–C13 for HOMO(–9). The HOMO's (–4 & –5) are substantially of σ character.


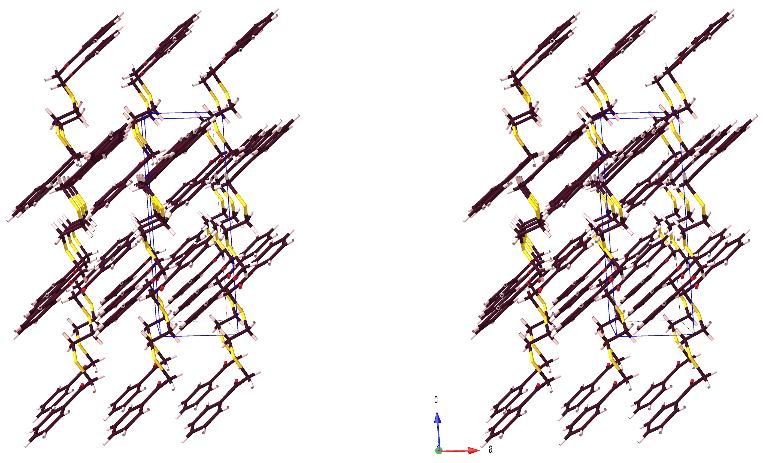


**Figure S7:** A view along the *b*-direction of a Dtdpe lattice fragment, showing the stepped structure of each molecule, and the parallelism of the phenyl groups (stick structure, inverse stereo).


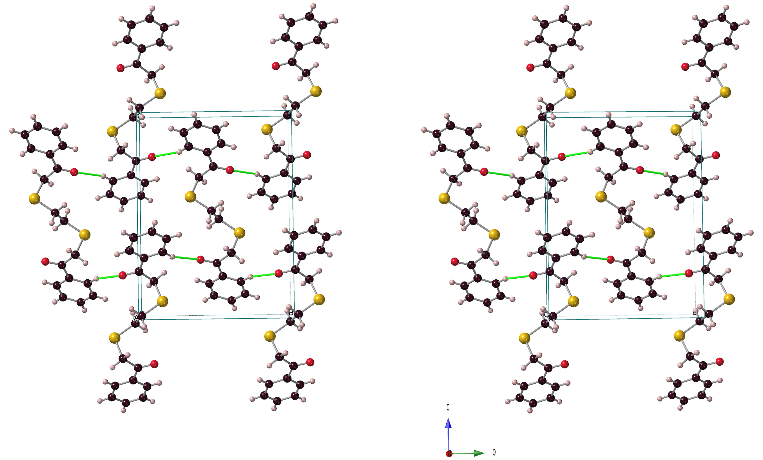


**Figure S8.** A lattice fragment of Dtdpe (ball/stick model, inverse stereoview), viewed along the *a*-direction and showing how weak intermolecular (C)H2---O1 interactions (green) network the lattice.


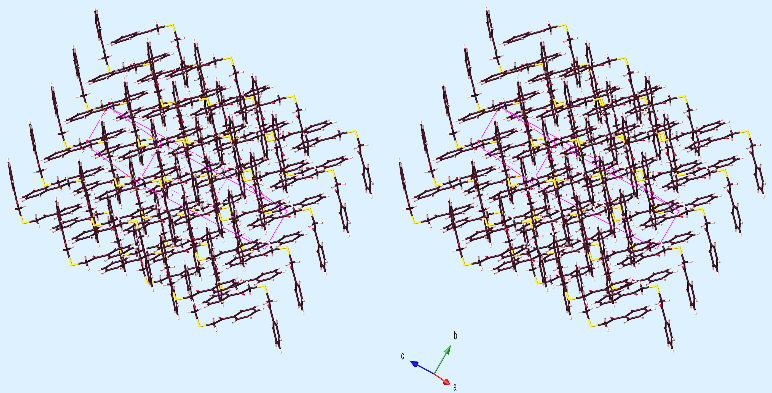


**Figure S9:** A view of the Mtdp lattice showing the close to rectilinear orientations of benzene rings (stick structure, inverse stereo).


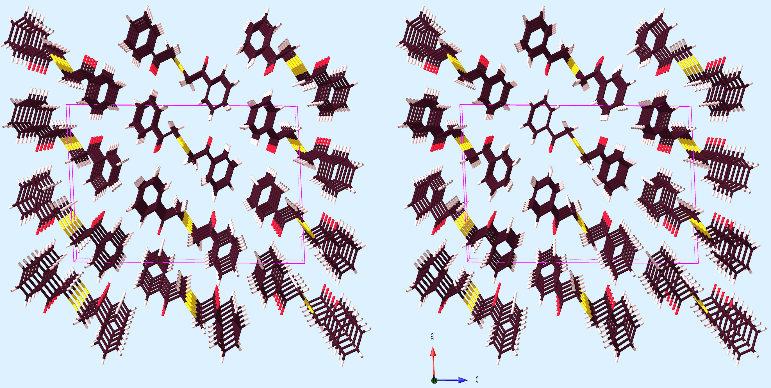


**Figure S10:** A view along the lattice *b*-direction, depicting the arraying of the Mtdp molecules thus (stick structure, inverse stereo).

**
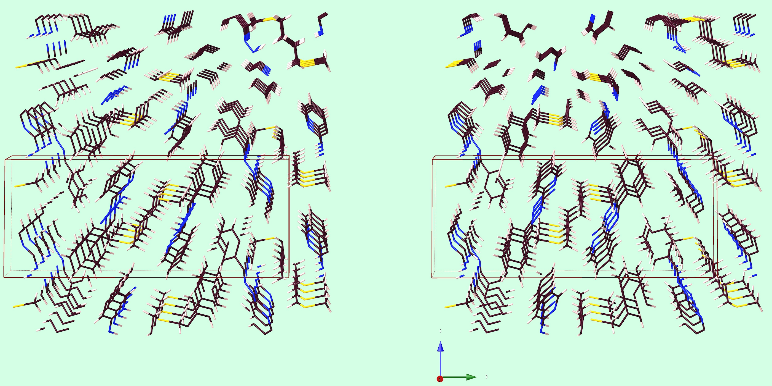
**

**Figure S11:** A Prpsb lattice fragment (stick model, inverse stereoview), viewed along, and showing the arraying of the molecules in the *a*-direction.


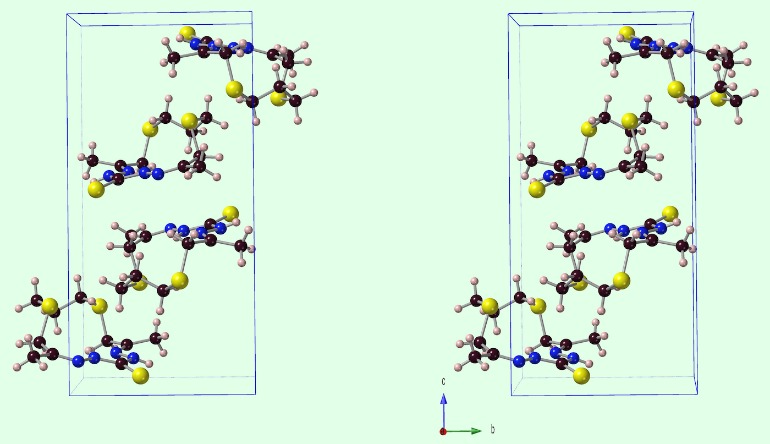


**Figure S12:** The *Z*= 4 molecular cell of Ctrsp viewed along the *a*-direction (ball/stick model, inverse stereo)


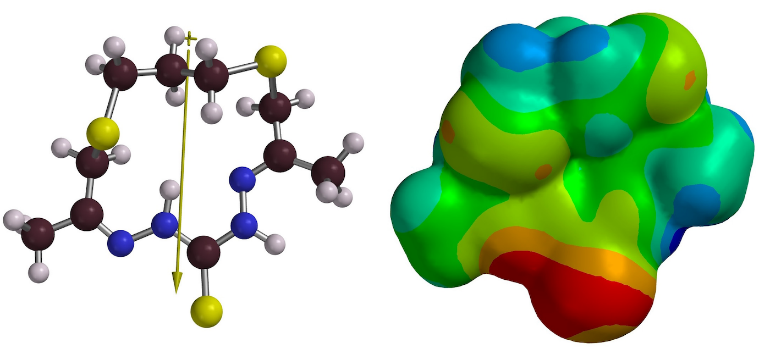


1. b)

**Figure S13** a) the dipole moment vector and b) the corresponding electrostatic potential map of Ctrsp (*Spartan*-20/24), with red indicating negative relative charge.


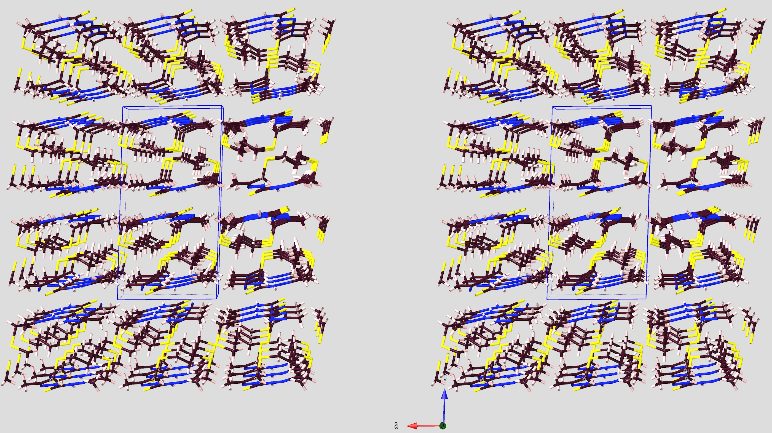


**Figure S14:** A view along the *b*-direction, showing the stacking of the Ctrsp *ab* double layers along the *c*-direction of the lattice (stick model, inverse stereo).


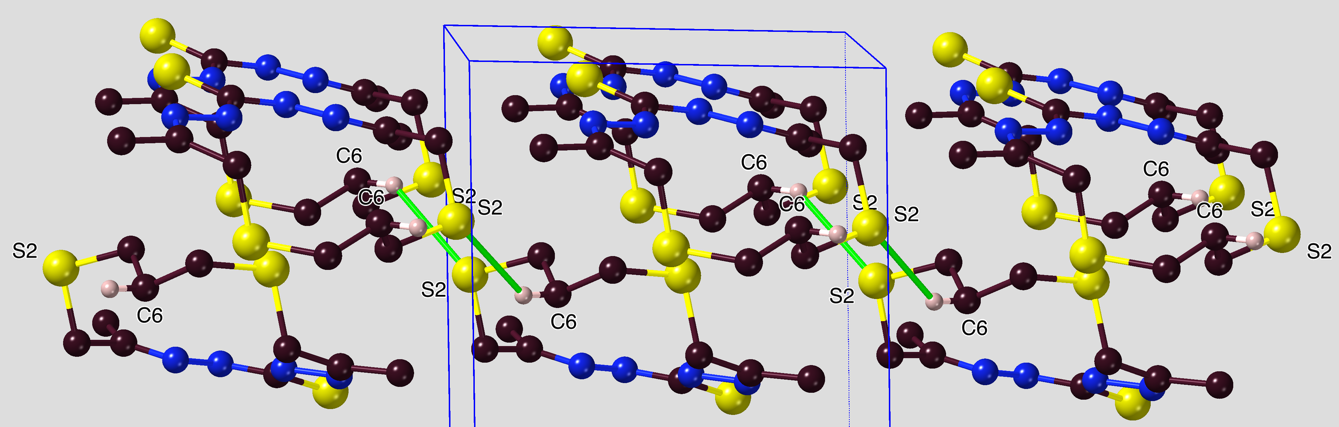


**Figure S15:** A view along the *b*-direction, showing the contacting of the *ab* layers by the (C6)H6A----S2 interactions (ball/stick model, inverse stereo), other H-atoms concealed.


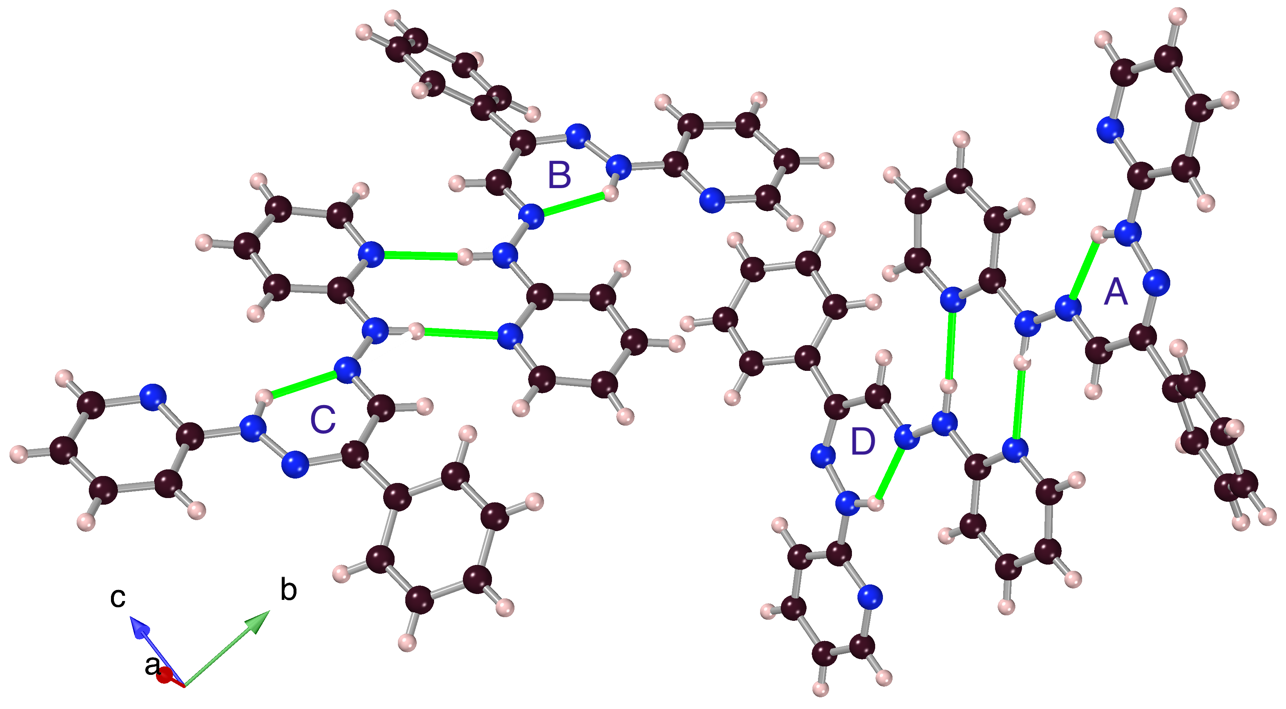


**Figure S16:** View approximately along the *a*-direction for the mutual arrangement of *A-D* and *B-C* dimers in the crystal lattice of Dhpk (ball & stick model).


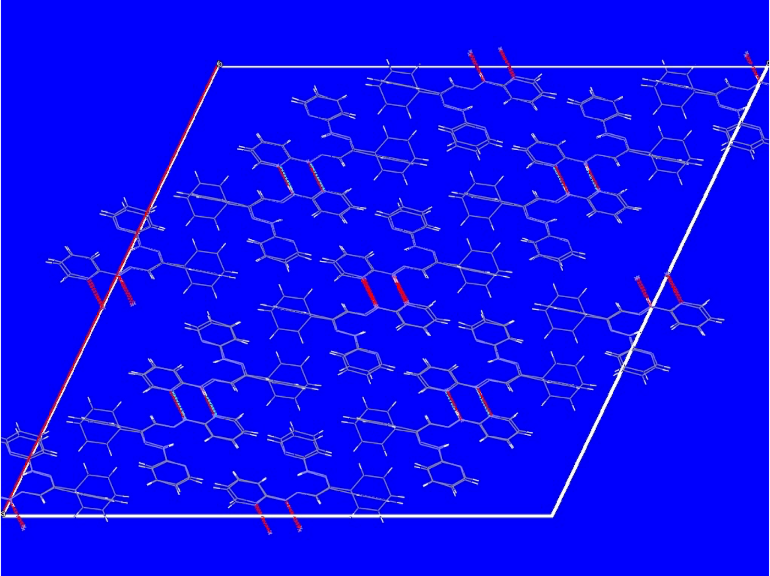


**Figure S17.** Stick model view along the *b*-direction for the dimers' strata in the unit cell of Dhpk.

**Table S1:** DFT calculations for intermolecular H-bonds.

|  |  |  |  |  |  |  | **Dimer** | **H--A** |  |  |  |  |
| --- | --- | --- | --- | --- | --- | --- | --- | --- | --- | --- | --- | --- |
|  | **Geometry** |  | **Basis** | **Energy** | **Energy** | **Dimer** | **kJ Energy** | **Bond** | **D–H** | **H---A** | **D---A** | **DĤA** |
|  | **Minmn. File^a^** | **Method** | **Set** | **Minmn. File** | **Value (E_h_)** | **Stabln. (E_h_)** | ***Advantage*** | **Order** | **(Å)** | **( Å)** | **( Å)** | **(Deg.)** |
| **Ctrsp** | NHS Dimer Gmin L | B3LYP | 6-311+G** | Dimer Emin M | -3611.363181 | 0.014711 | 38.6 | 0.3 | 1.03 | 1.40 | 3.41 | 167.9 |
| **Ctrsp** | Monomer | B3LYP | 6-311+G** | Monomer without | -1805.674235 | 0.014711 | 38.6 |  | 1.01 |  |  |  |
| **Ctrsp** | C6-S Dimer D4 Geom | ωB97X-D | 6-31G* | C6-S Dimer D5cEnerg | -3611.336746 | -0.011724 | -30.8 | *b* | 1.10 | 3.58 | 4.19 | 116.4 |
| **Dtdpe** | C2-O-Dimer S1 Geom | ωB97X-D | 6-31G* | C2ODimer U1c Energ | -3,287.732002 |  |  | 0.03 | 1.08 | 2.41 | 3.49 | 146.6 |
| **Dtdpe** | Monomer T1 | B3LYP | 6-311+G** | Monomer T1d Energ | -1,643.864709 | 0.002584 | +6.8 |  | 1.08 |  |  |  |
| **Mtdp** | Monomer-M1 Geom | B3LYP | 6-311+G** | Monomer-M2 Geom | -1167.000642 |  |  |  | 1.09 |  |  |  |
| **Mtdp** | O-H3-Dimer-N1 Geom | B3LYP | 6-311+G** | OH3Dimer-N 2 Energ | -2334.005015 | 0.003731 | +9.8 | *b* | 1.22 | 2.76*^c^* | 3.48*^c^* | 133.6*^c^* |
| **Prpsb** | Monomer Geom A1 | ωB97X-D | 6-31G* | Monomer Energy B3 | –2286.795468 |  |  |  | 1.08 |  |  |  |
| **Prpsb** | Monomer Geom J1 | B3LYP | 6-311+G** | Monomer Energy J2 | -2287.747455 |  |  |  | 1.08 |  |  |  |
| **Prpsb** | Dimer Gmin A1 | ωB97X-D | 6-31G* | Emin A1E | -4573.605938 | -0.015002 | 39.4 | 0.04 | 1.09 | 2.74*^c^* | 3.82 | 170.0*^c^* |
| **Dhpk** | Gmin Dimer N | B3LYP | 6-311+G** | Emin P5 | -2048.565734 | 0.016116 | 42.3 | 0.04 | 1.03 | 2.05*^c^* | 3.08*^c^* | 177.3 |
| **Dhpk** | Monomer, -intra T3 | B3LYP | 6-311+G** | Monomer Energy T4 | -1024.274809 | 0.016116 | 42.3 |  | 1.02 |  |  |  |

a: Intramolecular H-bonds are not specified for the calculations, and E_h_ values are quoted without any ESD estimate; b: Not calculated; c: Mean of the two values.


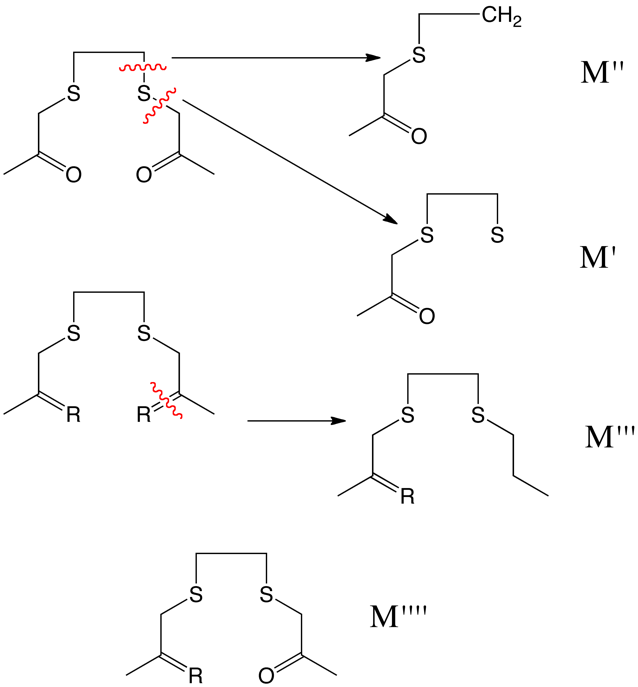


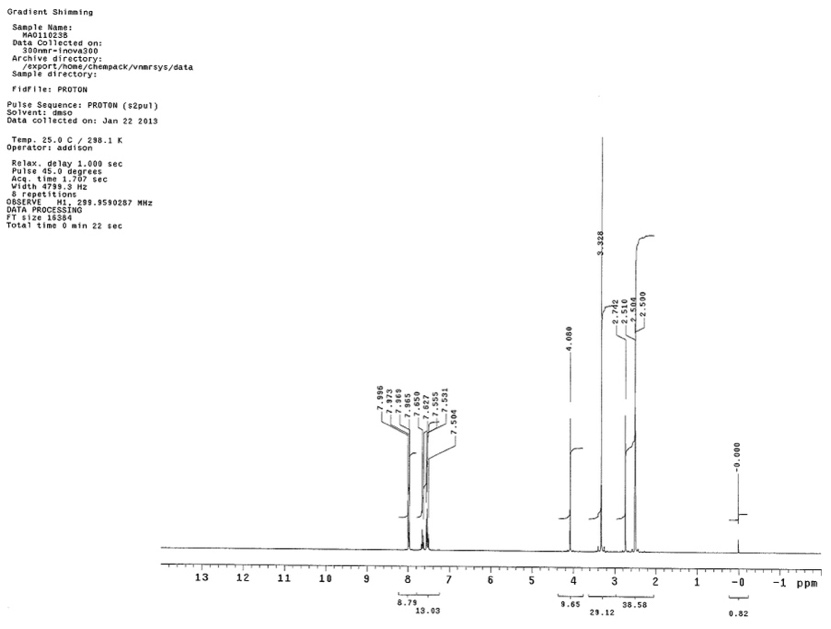
**Figure S18:** Fragment notations for mass spectrometry results.

**Figure S19:** ^1^H-NMR of Dtdpe.


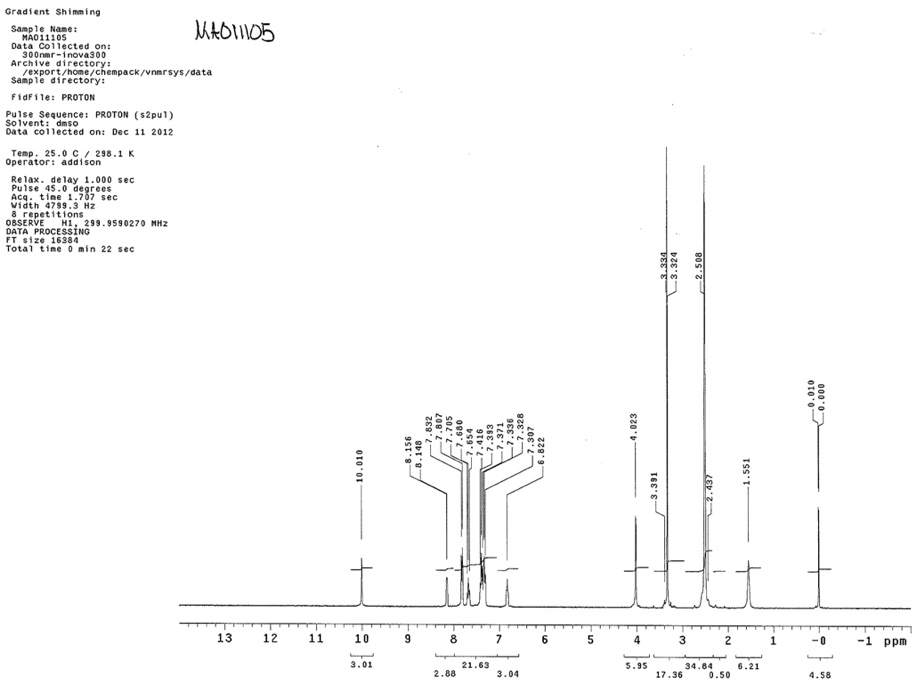


**Figure S20:** ^1^H-NMR of Prpsb


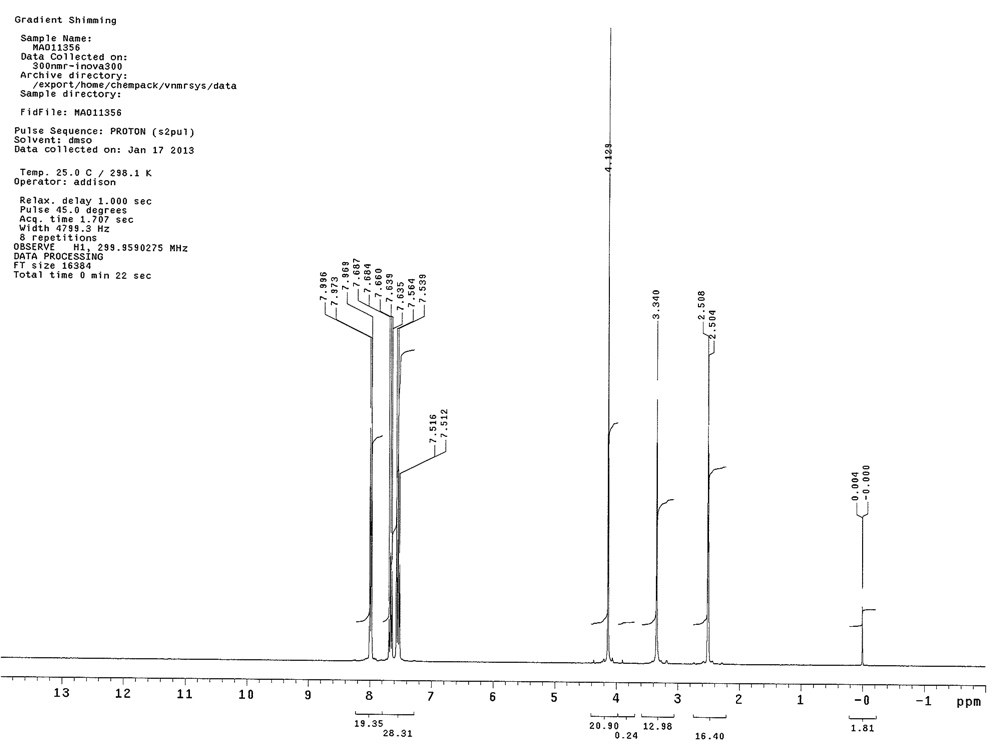


**Figure S21:** ^1^H-NMR of Mtdp.


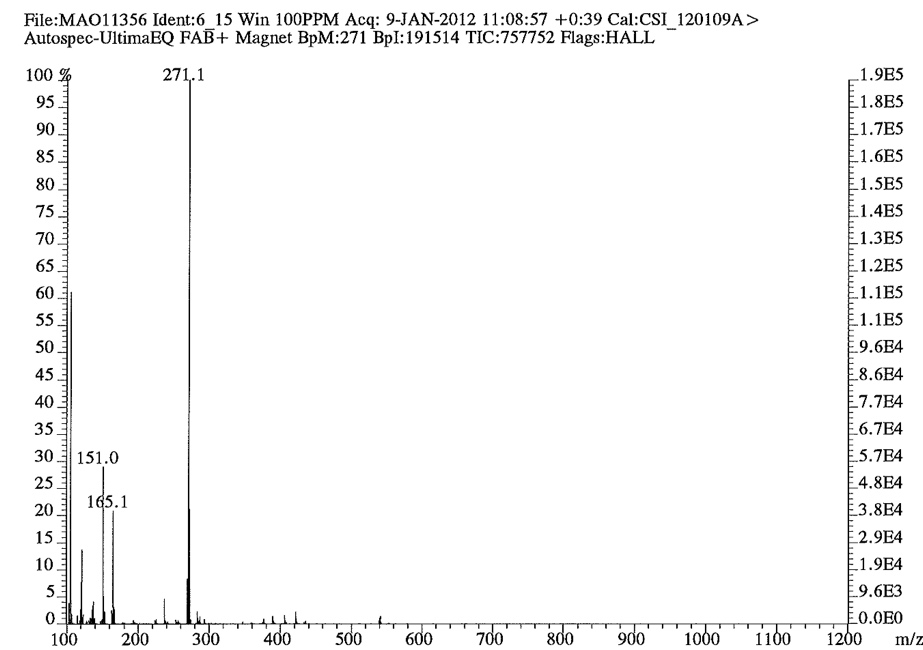


**Figure S22:** Mass spectrum of Mtdp: Calcd. for [C_16_H_14_O_2_S+H]^+^, 271.3


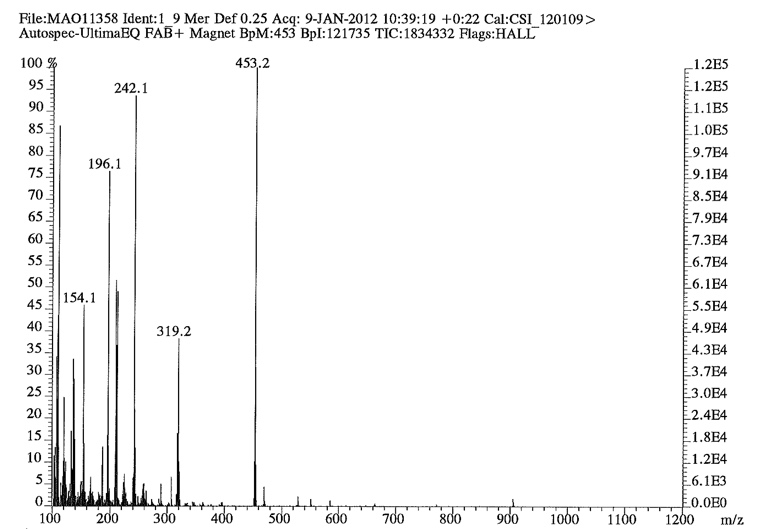


**Figure S23:** Mass spectrum from Mtph synthesis. For Mtph, [C_26_H_24_N_6_S+H]^+^= 453.6; for Dhpk, [C_18_H_16_N_6_+H]^+^= 317.1 The compound isolated by thermal recrystallisation from DMA or NMP was the latter.


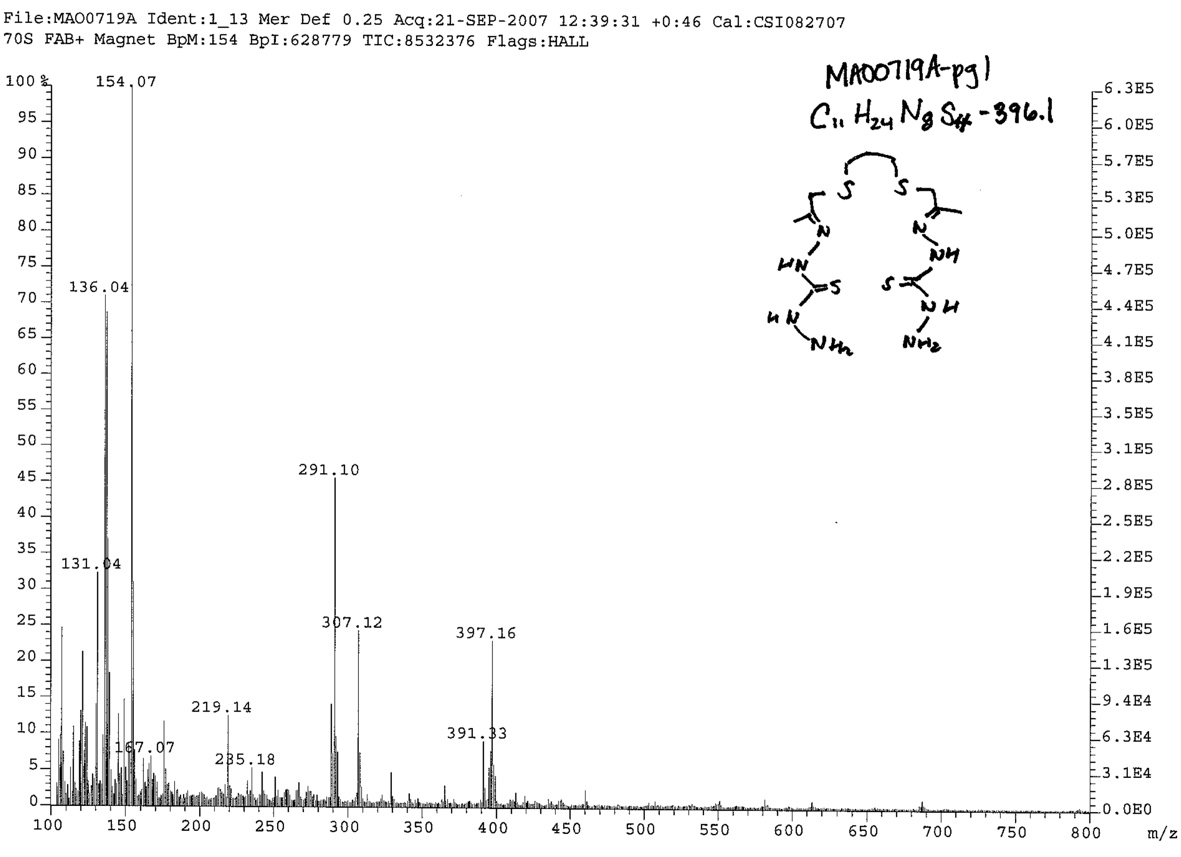


**Figure S24:** Mass spectrum from the synthesis of the thiocarbohydrazone derivative of Mtdp. Although the bis(thiocarbohydrazone) Tspm ([C_11_H_24_N_8_S_4_+H]^+^= 397.1) is indeed formed, it is dominated by the 1:1 macrocyclic product Ctrsp, with [C_10_H_18_N_4_S_3_+H]^+^= 291.1

**Abbreviations used , with matching prep. codes.**

Indexed below by the intersulfur carbons, the peripheral substituents and the derivative form.

Mtdk= Monothio dimethyl diketone: MAO11114, MAO11129, MAO11136

Mtdp= Monothio diphenyl diketone: MAO11356, MAO11138, MAO11158, MAO11294

Mtmh= Monothio dimethyl pyridylhydrazone: MAO11192

Mrq= Monothio, dimethyl, quinolylhydrazone: MAO11193

Mtph= Monothio diphenyl pyridylhydrazone: MAO11119, MAO11127, MAO11299, MAO11310, MAO11358

Dhpk= *Athio*-dihydrazone from crude Mtph: MAO11358

Mrpq= Monothio diphenyl quinolylhydrazone: MAO11128

Dtdke = Ethylene dimethyl diketone: MAO11111, MAO0930, MAO10130, MAO10166

Dtdpe= Ethylene diphenyl diketone: MAO11009, MAO11017, MAO11023, MAO11062

Prpse= Ethylene diphenyl pyridylhydrazone: MAO11026, MAO11066, MAO11201

Prsp= Propylene dimethyl pyridylhydrazone: MAO10117, MAO10139,

Dtdkp =Propylene dimethyl diketone: MAO0602, MAO0612, MAO0859, MAO10128, MAO10167, MAO11096

Prse= Ethylene dimethyl pyridylhydrazone: MAO0914, MAO11104, MAO11116,

Tspm= Propylene dimethyl thiocarbohydrazone: MAO0719, MAO0727, MAO0728

Ctrsp= Cyclic mono-thiocarbohydrazone of propylene dimethyl diketone: MAO0719A

Qrsp= Propylene dimethyl quinolylhydrazone: MAO10169

Dtdpp= Propylene diphenyl diketone: MAO0918, MAO11113

Prpsp= Propylene diphenyl pyridylhydrazone: MAO11116

Prpsq= Propylene diphenyl quinolylhydrazone: MAO11144

Dtdkb= Butylene dimethyl diketone: MAO10168, MAO10129, MAO11097

Prsb= Butylene dimethyl pyridylhydrazone: MAO10129, MAO10168, MAO11083

Dtdpb= Butylene diphenyl diketone: MAO0858, MAO11019, MAO11021, MAO10129, MAO10181, MAO11063

Prpsb= Butylene diphenyl pyridylhydrazone: MAO11067, MAO11105, MAO11123

Dtdkx = Xylylene dimethyl diketone: MAO11013, MAO11131

Prsx= Xylylene dimethyl pyridylhydrazone: MAO11014

Dtdpx= Xylylene diphenyl diketone: MAO11132

Prpsx= Xylylene diphenyl pyridylhydrazone: MAO11145

Rsp = Propylene dimethyl dihydrazone: MAO0908

Rsb= Butylene dimethyl dihydrazone: MAO11084

Rspb= Butylene diphenyl dihydrazone: MAO11083
